# Targeting Replication Fork Processing Synergizes with PARP Inhibition to Potentiate Lethality in Homologous Recombination Proficient Ovarian Cancers

**DOI:** 10.1002/advs.202410718

**Published:** 2025-03-16

**Authors:** Ganesh Pai Bellare, Kshama Kundu, Papiya Dey, Krupa Thankam Philip, Nitish Chauhan, Muskan Sharma, Sankarsingh Kesharsingh Rajput, Birija Sankar Patro

**Affiliations:** ^1^ Bio‐Organic Division Bhabha Atomic Research Centre Mumbai 400085 India; ^2^ Homi Bhabha National Institute Anushaktinagar Mumbai 400094 India

**Keywords:** *trans*‐4,4′‐dihydroxystilbene (DHS), DNA replication, PARP inhibitor, ssDNA gaps, talazoparib

## Abstract

Synthetic lethality in homologous recombination (HR)‐deficient cancers caused by Poly (ADP‐ribose) polymerase inhibitors (PARPi) has been classically attributed to its role in DNA repair. The mode of action of PARPi and resistance thereof are now believed to be predominantly replication associated. Therefore, effective combinatorial approaches of targeting replication fork processing along with HR‐downregulation to target HR‐proficient and possibly PARPi‐resistant tumors are warranted. Stilbenes are a privileged class of molecules, which include resveratrol, pterostilbene, piceatannol, *etc*, that modulate both replication processes and RAD51‐expression. In this investigation, by screening a small library of stilbenes, including in‐house synthesized molecules, *trans*‐4,4′‐dihydroxystilbene (DHS) was discovered as a potent natural agent, which downregulates RAD51 expression and HR repair (GFP‐reporter assay). DHS induces extensive synergistic cell death in ovarian cancers when combined with talazoparib (PARPi). Mechanistically, DHS elicits replication‐stress through severely impeding replication fork progress, speed, and inducing fork‐asymmetry. This leads to robust induction of single stranded DNA (ssDNA) gaps and poly‐ADP‐ribosylation (PARylation) in S‐phase cells, signifying issues related to lagging (Okazaki) strand synthesis. PARPi, which abrogates PARylation, potentiates DHS induced ssDNA gaps, and their conversion into lethal double strand breaks through MRE11 action. Furthermore, the combination is highly effective in mitigating ovarian tumor xenograft growth in SCID mice and exhibited a good therapeutic‐index with no/minimal tissue‐toxicity.

## Graphical abstract

This study demonstrates that the resveratrol analogue, *trans*‐4,4′‐dihydroxystilbene (DHS), exhibits synergistic anti‐tumor activity with the PARP inhibitor talazoparib, inducing significant ovarian cancer cell death. The synergistic effect of this combination arises from the concurrent downregulation of homologous recombination factors and the induction of acute replicative stress, culminating in the collapse of stalled replication forks through MRE11‐mediated fork processing.

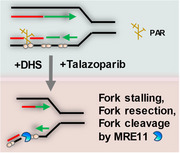

## 1 Introduction

Poly‐(ADP‐ribose) polymerase inhibitors (PARPi) have shown impressive efficacy and are now considered a standard of care for homologous recombination deficient (HRD) breast, ovarian, prostate, and pancreatic cancers.^[^ ^1^, ^2^, ^3^ ^]^ However, in view of the incidences of resistance to PARPi therapy in HRD cancers under experimental and clinical scenario,^[^ ^4^ ^]^ their mode of action earlier purported on the lines of synthetic lethality are now either being added to with more intricate roles for PARPs, and/or are being revisited.^[^ ^5^, ^6^, ^7^ ^]^ Moreover, patients with homologous recombination proficient (HRP) tumors are also now recommended the usage of PARP inhibitors for maintenance therapy considering the efficacy of the new generation PARP inhibitors.^[^ ^8^, ^9^ ^]^ Besides, recent reports have demonstrated significant anti‐cancer efficacy of PARPi in certain cancer sub‐types, irrespective of the homologous recombination status.^[^ ^10^ ^]^

Ovarian cancer is a dreaded gynecological malignancy with one of the lowest 5‐year survival rates. Among the different ovarian cancers, epithelial ovarian cancers account for ∼90% of the diagnoses.^[^ ^11^ ^]^ Chemotherapy and PARPi maintenance therapy are favored in patients with HRD, which significantly enhance the overall survival (OS). However, major clinical challenge lies in treating ∼50% HRP patients who show poor prognosis associated with primary platinum and PARPi resistance, and consequently, shorter OS. Moreover, relapsed platinum‐ and PARPi‐resistant cases progress from HRD to HRP, due to additional genetic/epigenetic changes.^[^ ^12^ ^]^ Hence, HRP ovarian cancers pose a major hurdle for efficient treatment and clinical management. In this regard, several recent reports have advocated for the application of rational combinatorial regimen of PARPi with adjuvants to impair homologous recombination (HR) to sensitize HRP cancers in clinical and preclinical settings. In this context, targeting HR through inhibition of CARM1‐EZH2, RECQL5, TOPBP1, and autophagy by small molecules, led to sensitization of HRP cancers to PARPi.^[^ ^13^, ^14^, ^15^, ^16^ ^]^

PARP1 plays a pivotal role in the processing and ligation of Okazaki fragments (OF), during lagging strand synthesis, ensuring fork stability.^[^ ^17^, ^18^ ^]^ PARP inhibition along with the targeting of replication dynamics enhances replication‐associated single strand DNA (ssDNA) gaps, fork degradation, fork collapse, leading to sensitization of HRP cancers.^[^ ^19^, ^20^, ^21^, ^22^ ^]^ However, toxicity associated with simultaneous inhibition of PARP and DNA replication associated/ regulating targets such as ATR, CHK1, and WEE1 pose special challenge in identifying clinically tolerable dosing schedule.^[^ ^22^, ^23^ ^]^ With this backdrop, we hypothesized that combining PARPi with a natural molecule, which can simultaneously downregulate HR and inflict replication stress may lead to increased DNA double‐strand breaks (DSBs) and consequently, enhanced cell death in HRP ovarian cancers. Resveratrol and its analogues are privileged natural molecules which possess the ability to downregulate HR repair (HRR) while simultaneously perturbing replication dynamics.^[^ ^24^, ^25^ ^]^ Inhibition of DNA polymerase activity by resveratrol and its analogues has also been reported, which implies an eventual DNA replication suppression.^[^ ^26^ ^]^ Inspired by these attributes of resveratrol, which has a stilbene moiety at its core structure, our research aimed at identifying stilbene congeners with potent RAD51 downregulating properties. From a small library of 20 stilbene molecules, which differ in the side chains attached to the stilbene backbone, we screened‐in *trans*‐4,4′‐dihydroxystilbene (DHS) for further investigation. DHS is a natural stilbene molecule, available abundantly in the *Yucca periculosa* plant. Our investigation showed that DHS is superior to resveratrol in downregulating of RAD51 expression and inducing replication stress in ovarian cancers. Moreover, we and others have previously reported the anti‐cancer effects of DHS alone treatment on melanoma, neuroblastoma and other cancers.^[^ ^27^, ^28^, ^29^ ^]^

In the present work, we asked whether co‐treatment of DHS for simultaneous targeting of DNA replication and HRR could enhance ovarian cancer cell death induced by PARP inhibitors. Our data shows that replicative stress mediated by DHS efficiently synergizes with PARP inhibitors in inducing cell death in HRP ovarian cancer. Mechanistically, DHS induces acute suppression of replication fork progression, enhances poly‐ADP‐ribosylation (PARylation) and ssDNA gaps behind the fork, possibly at the lagging strands, eventually leading to MRE11‐mediated fork collapse and sensitization of ovarian and other cancers to PARPi (talazoparib). Moreover, the combination of DHS *plus* talazoparib was non‐toxic, well‐tolerated in animal models and effectively reduced SK‐OV‐3 xenograft tumor burden in vivo immunodeficient SCID mice model with minimal/no normal tissue toxicity.

## 2 Results

### 2.1 Screening of Resveratrol Analogues for the Downregulation of HR Proteins in Cancer Cells

**Figure 1:**
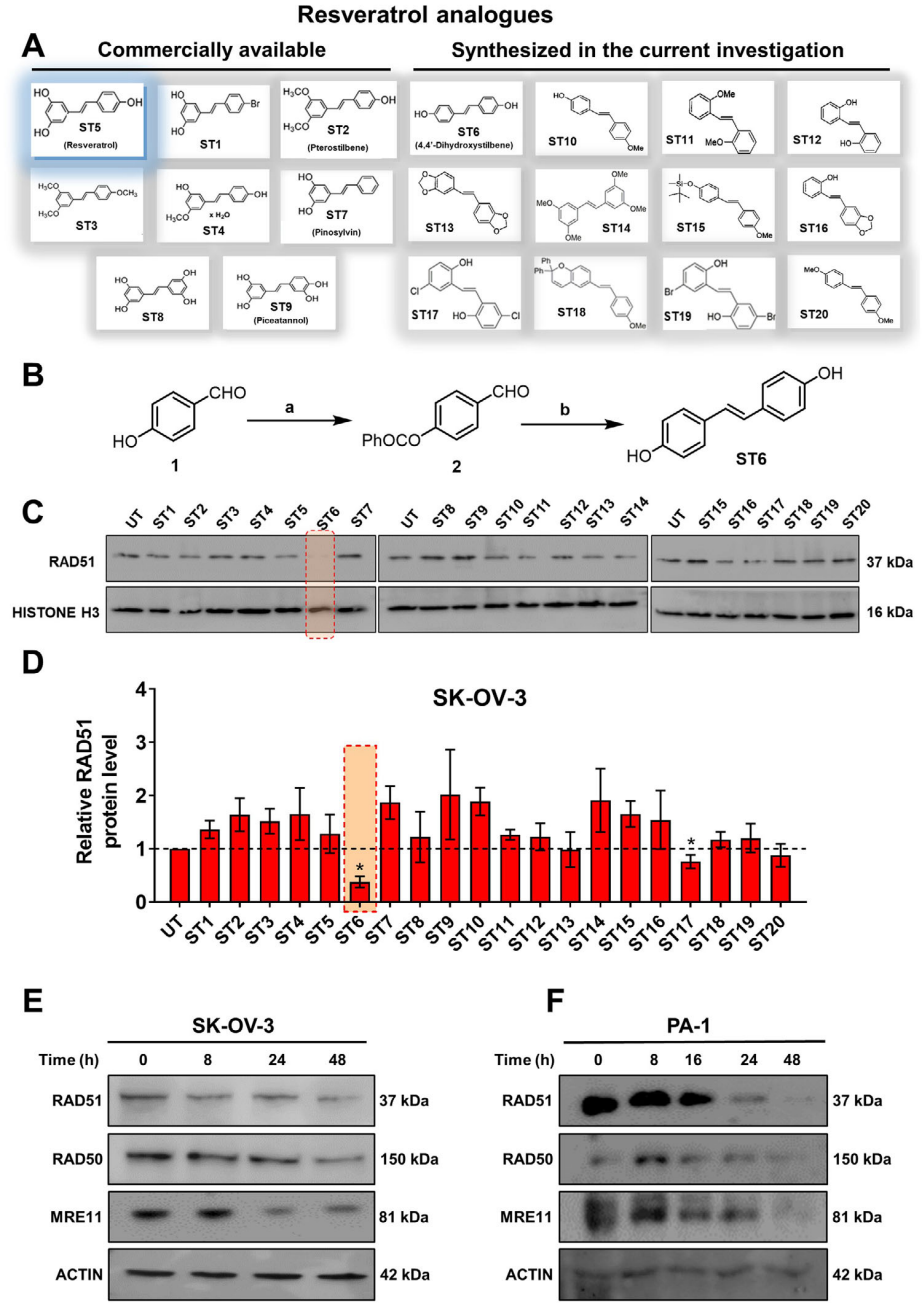
Screening of a library of small stilbene molecules for downregulation of HR factors in cancer. A) Chemical structures of the 20 stilbene molecules (**ST1‐ST20**), including resveratrol (**ST5**) used in the screen. B) Synthesis scheme for DHS (**ST6**). Reagents and conditions: a) BzCl/ NEt_3_/ DCM/ 0 °C‐RT/ 2.5 h; b) TiCl_4_/ Zn/ THF/0 °C‐reflux/ 3 h. C, D) RAD51 downregulation by stilbenes: SK‐OV‐3 ovarian cancer cells were treated with vehicle or **ST1‐ST20** (10 µM each) for 24 h and the expression of RAD51 was evaluated by Western blotting. Image of the western blotting of one of the replicates is shown in C. RAD51 protein level intensity was normalized to the band intensity of the untreated (UT) sample. Quantification of the western blotting is indicated in D. (N = 3 biological replicates) Values indicated are mean ± S.E.M. *p < 0.05 compared to the respective untreated group (ANOVA). E, F) Downregulation of HR factors by DHS: SK‐OV‐3 and PA‐1 ovarian cancer cells were treated with vehicle or DHS (SK‐OV‐3: 10 µM, PA‐1: 100 nM) for the indicated time periods and expression of HR factors were assessed by western blotting.

In order to identify a small molecule targeting HR, we screened a small library of stilbene/ resveratrol analogues. Studies have demonstrated that subtle structural modifications, especially changes in the number, position, and functionalization of hydroxyl (‐OH) groups, significantly impact resveratrol's binding affinity to its target and biological activity.^[^ ^30^ ^]^ With this understanding, we employed a rational design approach, preserving the core stilbene structure while modifying the number, position, and functionalization of ‐OH groups in resveratrol. In this regard, we have chosen 8 commercially available resveratrol analogues and another 12 in‐house synthesized molecules (**Figure** **1**A). These resveratrol analogues have stilbene moiety as their common core structure, hence named as **ST1** to **ST20** (Figure 1A; **ST5**: Resveratrol, **ST2**: Pterostilbene, **ST9**: Piceatannol). Details of synthesis and characterization of **ST6** and **ST10‐ST20** are included in the methods and Figures S1–S25 (Supporting Information). For synthesis of **ST6** in gram scale, we used modified version of reported protocols for low‐valent titanium mediated reactions with 4‐formylphenyl benzoate (Figure 1B; Figures S1–S4, Supporting Information).^[^ ^26^, ^31^ ^]^ Of note, these stilbene molecules are structural variants of resveratrol molecule with respect to (i) number of ‐OH groups, (ii) position of ‐OH groups and (iii) modifications on ‐OH groups. We and others have previously reported that resveratrol downregulates RAD51 expression and inhibits HR.^[^ ^24^, ^32^ ^]^ We therefore screened the 20 stilbene molecules (**ST1‐ST20**) to identify a potent RAD51‐downregulating small molecule in ovarian cancer cells. Our results revealed that treatment of SK‐OV‐3 cells, epithelial human ovarian carcinoma, with these resveratrol analogues (10 µM, 24 h) led to differential expression of RAD51 (Figure 1C,D). Amongst the screened stilbenes, **ST6, ST17**, and **ST20** showed relative downregulation of RAD51 expression while other stilbenes showed either no significant effect or upregulated RAD51 protein expression. Interestingly, **ST6** (*trans*‐4,4′‐dihydroxystilbene; DHS) could potently downregulate the expression of RAD51 in SK‐OV‐3 cells. Similar results were also observed in another ovarian carcinoma cells (PA‐1) (data not shown). We also observed that the DHS could downregulate MRE11, RAD50 of the MRN complex of proteins as well as RAD51 in SK‐OV‐3 and PA‐1 cells in a time dependent manner (Figure 1E,F). However, BRCA1 and BRCA2 levels were not significantly altered in the presence of the DHS treatment (Figure S26, Supporting Information), suggesting the ability of DHS to potently downregulate some of the key proteins of HR machinery. Considering the aforementioned points and the impressive HR‐downregulating activity, DHS was chosen for our further investigation.

### 2.2 The Combination of PARPi (Talazoparib) and DHS Induces Synergistic Cell Death of Cancer Cells

**Figure 2:**
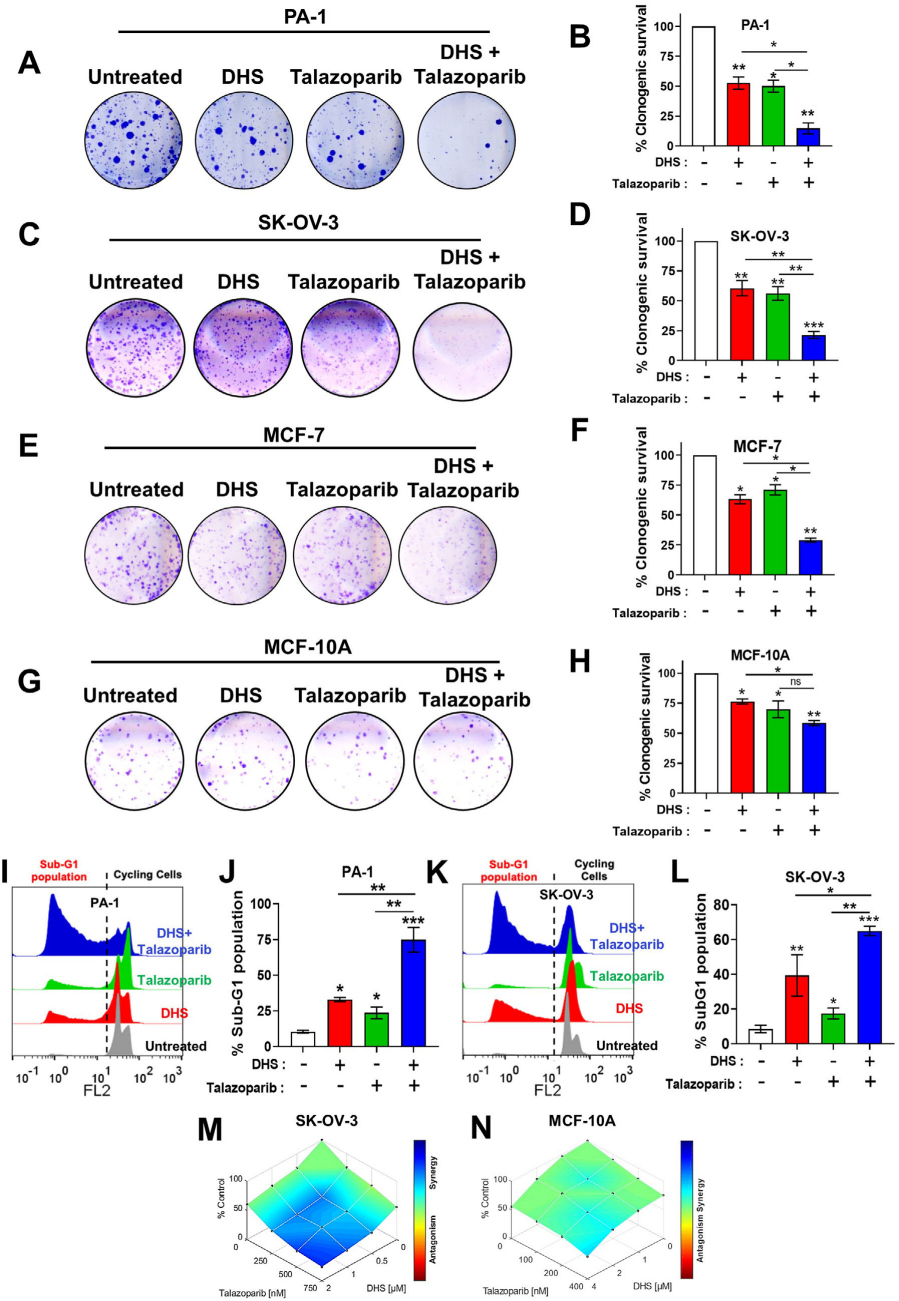
Assessment of colony formation in cancer cells following DHS and talazoparib treatments. A‐D) PA‐1 and SK‐OV‐3 ovarian cancer cells were treated with vehicle control or DHS, talazoparib alone or combination of talazoparib *plus* DHS for 7–10 days, and colony formation was assessed by clonogenic assay. Quantifications of the clonogenic survival data are shown. Concentrations of DHS were 100 nM and 2 µM for PA‐1 and SK‐OV‐3, respectively. Concentrations of talazoparib were 100 nM and 750 nM for PA‐1 cells and SK‐OV‐3, respectively. E‐H) MCF‐7 and MCF‐10A cells were treated with DHS, talazoparib or their combination for 7–10 days, and colony formation was assessed by clonogenic assay. Quantifications of the clonogenic survival data are shown. Concentrations of DHS were 2 µM while concentrations of talazoparib were 6.25 nM and 200 nM for MCF‐7 and MCF‐10A cells, respectively. I‐L) PA‐1 cells were treated with DHS (625 nM), talazoparib (200 nM) or their combination for 48 h, and the apoptotic population was assessed by sub‐G1 assay. Similarly, SK‐OV‐3 cells were treated with DHS (10 µM), talazoparib (200 nM) or their combination for 72 h, and the apoptotic population was assessed by sub‐G1 assay. Quantifications of the apoptotic cell death are shown. M, N) SK‐OV‐3 and MCF‐10A cells were treated with different concentrations of DHS and talazoparib and their combination for 7–10 days. Clonogenic assay data were analysed by Combenefit and plots for the interaction of drugs in inducing cell death is shown. (*N* = >3 biological replicates) Values indicated are mean ± S.E.M. *p < 0.05, **p < 0.01 and ***p < 0.001 compared to the untreated group or between the groups being compared (ANOVA with Tukey post‐hoc analysis). Intergroup comparisons are indicated.

Since DHS downregulated HR machinery, we sought to know whether DHS can enhance the effects of talazoparib, an efficient new‐generation PARPi, in HR proficient ovarian cancers. There was a dose‐dependent decrease in the clonogenic survival of both PA‐1 and SK‐OV‐3 ovarian cancer cells with increasing concentrations of DHS and talazoparib (Figure S27A–H, Supporting Information). In the clonogenic assay, the IC_50_ values for DHS and talazoparib were found to be ∼125 nM and ∼140 nM in PA‐1 cells, and ∼3.3 µM and ∼0.7 µM in SK‐OV‐3 cells, respectively (Figure S27A–H, Supporting Information). SK‐OV‐3 was relatively resistant to talazoparib and DHS. Interestingly, combination of talazoparib *plus* DHS, at their near IC50 concentrations, was significantly effective in reducing clonogenic potential of both the ovarian cancer cells (**Figure** **2**A–D). Further, the combination treatment also showed synergistic activity in two other BRCA1/2 wild‐type ovarian cancer cell lines, OAW42 and A2780 (Figure S28A–D, Supporting Information). Moreover, our results revealed that the higher efficacy of combination of talazoparib *plus* DHS was not limited to ovarian cancer cells, as similar enhanced effects were also observed in breast cancer cells (MCF‐7; Figure 2E,F). Of note, the individual treatment of talazoparib and DHS or their combination had lesser impact on the clonogenic survival of normal mammary epithelial cells (MCF‐10A; Figure 2G,H), suggesting that combination treatment of talazoparib *plus* DHS is effective against HR‐proficient cancers.

Elimination of cancer cells by apoptosis/programmed cell death is the mainstay of clinical anti‐cancer therapy. Our flow cytometry data for sub‐G1 population (apoptosis) analysis revealed that the combination treatment of talazoparib *plus* DHS induced robust apoptosis in both PA‐1 and SK‐OV‐3 ovarian cancer cells *vis‐à‐vis* individual treatments (Figure 2I–L), suggesting a role for DHS in promoting talazoparib induced cell death. In comparison to resveratrol, DHS, alone and in combination with talazoparib, induced significantly higher apoptosis, indicating superior efficacy of DHS over resveratrol (Figure S29A–C, Supporting Information). Considering the efficacy of combination of talazoparib *plus* DHS in the induction of cell death, we assessed the interaction between talazoparib and DHS, employing Combenefit analysis.^[^ ^33^ ^]^ Combenefit analysis indicated that the molecules acted synergistically in reducing the clonogenic survival of SK‐OV‐3 cancer cells, while this effect was majorly additive in non‐malignant MCF‐10A cells (Figure 2M,N).

Our data showed that PA‐1 was sensitive while SK‐OV‐3 was relatively resistant to talazoparib alone (IC_50_: ∼728 nM). In patients and preclinical studies, the maximum plasma concentration of talazoparib was observed in the range of 20–80 nM, after single/multiple oral administrations of this drug.^[^ ^15^, ^34^ ^]^ In order to evaluate whether DHS is able to promote talazoparib induced reduction of clonogenic survival at physiologically relevant concentration, a combination treatment of talazoparib at 25 and 50 nM with DHS in SK‐OV‐3 cells was assessed. Strikingly, DHS synergistically reduced clonogenic survival of SK‐OV‐3 cells even in response to lower dose of talazoparib (25 and 50 nM, i.e., at least ≈15 times lower than the IC_50_ value) (Figure S29D–F, Supporting Information). Collectively, the combination of DHS and talazoparib was highly effective and synergistic against different HR‐proficient cancer cells. For further investigation on the mechanistic evaluation of synergistic effect of combination treatment, the concentration of talazoparib and DHS were chosen as 50 nM and 10 µM, respectively, to effectively understand the mechanistic details leading to cell death. However, certain crucial experiments were also performed at lower concentrations of DHS to validate these findings.

### 2.3 DHS *Plus* Talazoparib Treatment Suppresses Homologous Recombination in Cancers

**Figure 3:**
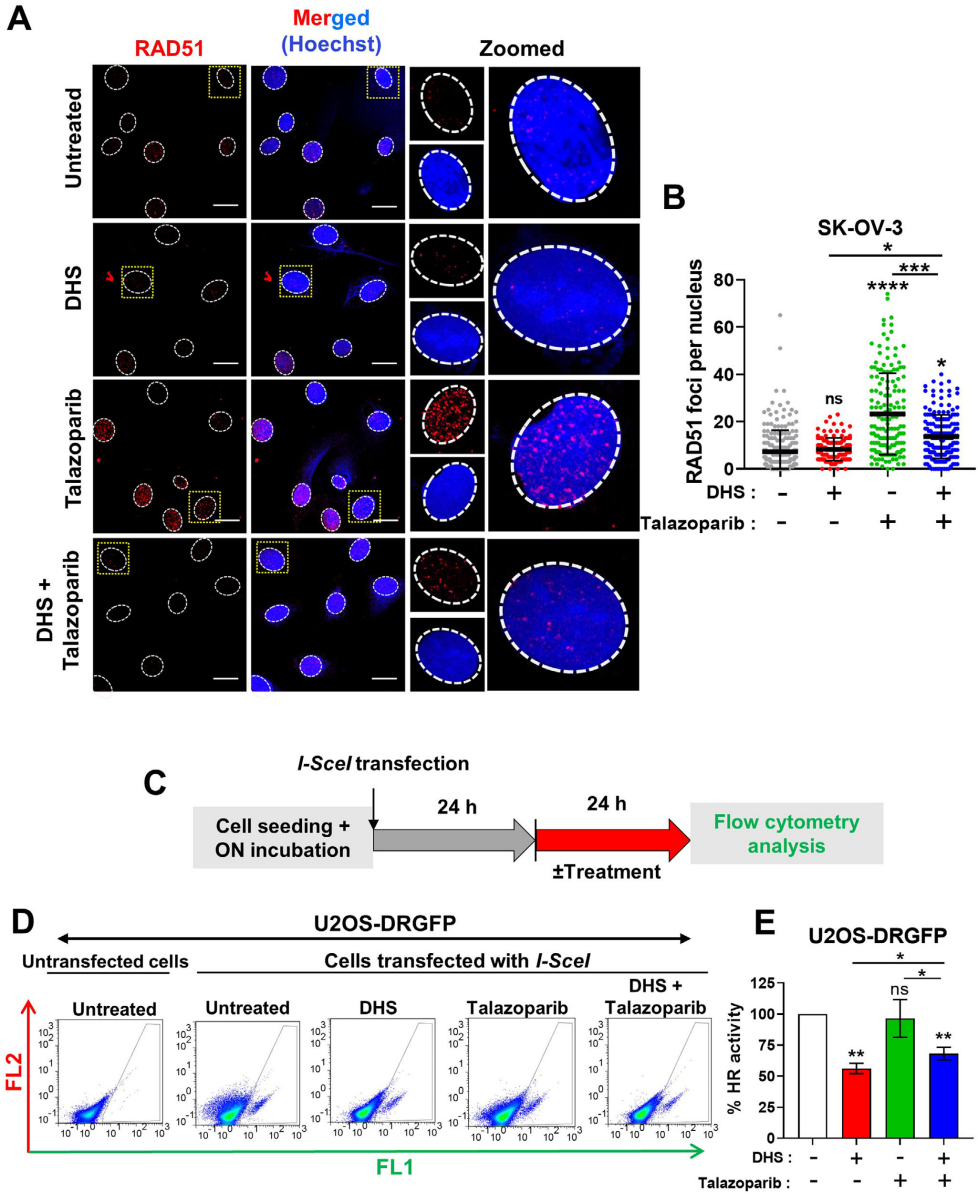
The effects of DHS and talazoparib on homologous recombination repair in cancer cells. A, B) SK‐OV‐3 cells were treated with DHS (10 µM), talazoparib (50 nM), or the combination for 24 h. Immunofluorescence analysis of RAD51 foci was carried out, RAD51 foci per nucleus were counted and plotted. Values indicated are Mean ± S.D. (N = 3 biological replicates) Scale bar: 20 µm. C) Scheme for the DR‐GFP HR reporter assay. D, E) U2OS cells stably expressing DR‐GFP HR reporter construct were transfected with *pCBA‐ISceI* plasmid construct. Post 24 h, these cells were treated with DHS (10 µM), talazoparib (50 nM), or the combination of the two agents. Flow cytometry was used to assess GFP^+^ cells 24 h post treatment following appropriate application of population gating. % HR activity was calculated with respect to the GFP^+^ cells in the gated population of the untreated sample. Mean ± S.E.M. is indicated (N = 3 biological replicates). ns: non‐significant, *p < 0.05, **p < 0.01, ***p < 0.001 and ****p < 0.0001 compared to the respective untreated group or between the groups being compared (ANOVA with Tukey post‐hoc analysis). Inter‐group comparisons are indicated.

PARPi induces synthetic lethality in HR deficient cancers while HR proficient cancers are sensitive to PARPi in the presence of HR downregulating agents. Since DHS downregulated the expression of key proteins of HR machinery, we sought to know whether DHS is able to modulate talazoparib‐induced HR repair in cancer. Immunofluorescence detection of RAD51 foci formation is an important surrogate for the assessment of HR in cells.^[^ ^35^, ^36^ ^]^ As shown in **Figure** **3**A,B, RAD51 foci formation was not reduced in DHS alone treated cells, suggesting that although RAD51 expression is reduced by DHS (Figure 1C,D), residual proteins may still be able to form RAD51 foci. Interestingly, however, talazoparib elicited extensive RAD51 foci formation in SK‐OV‐3 cells, which was significantly reduced by DHS in the combination treatment. Although RAD51 foci are surrogate indicators of ongoing HR repair in cells, they may not truly reflect the efficient completion of HR repair of DNA damage. In order to assess the execution and completion of HR repair, a DR‐GFP plasmid‐based HR reporter assay^[^ ^37^ ^]^ was used (Figure 3C). Level of GFP fluorescence directly corresponds to the activity of HR in repairing the DSB generated by *I‐SceI* restriction endonuclease at the DR‐GFP break site.^[^ ^38^ ^]^ In this context, we observed a significant downregulation of HR activity‐induced expression of DR‐GFP in cancer cells in response to both DHS alone and talazoparib *plus* DHS treatment (Figure 3C–E). Together, DHS can efficiently abrogate talazoparib‐induced HR repair process, which may contribute to its ability to elicit synergistic lethality in ovarian cancers in response to talazoparib.

### 2.4 DHS *Plus* Talazoparib Combination Treatment Results in Impaired Cell Cycle Progression, S‐Phase Arrest, and Defective Replication Dynamics

**Figure 4:**
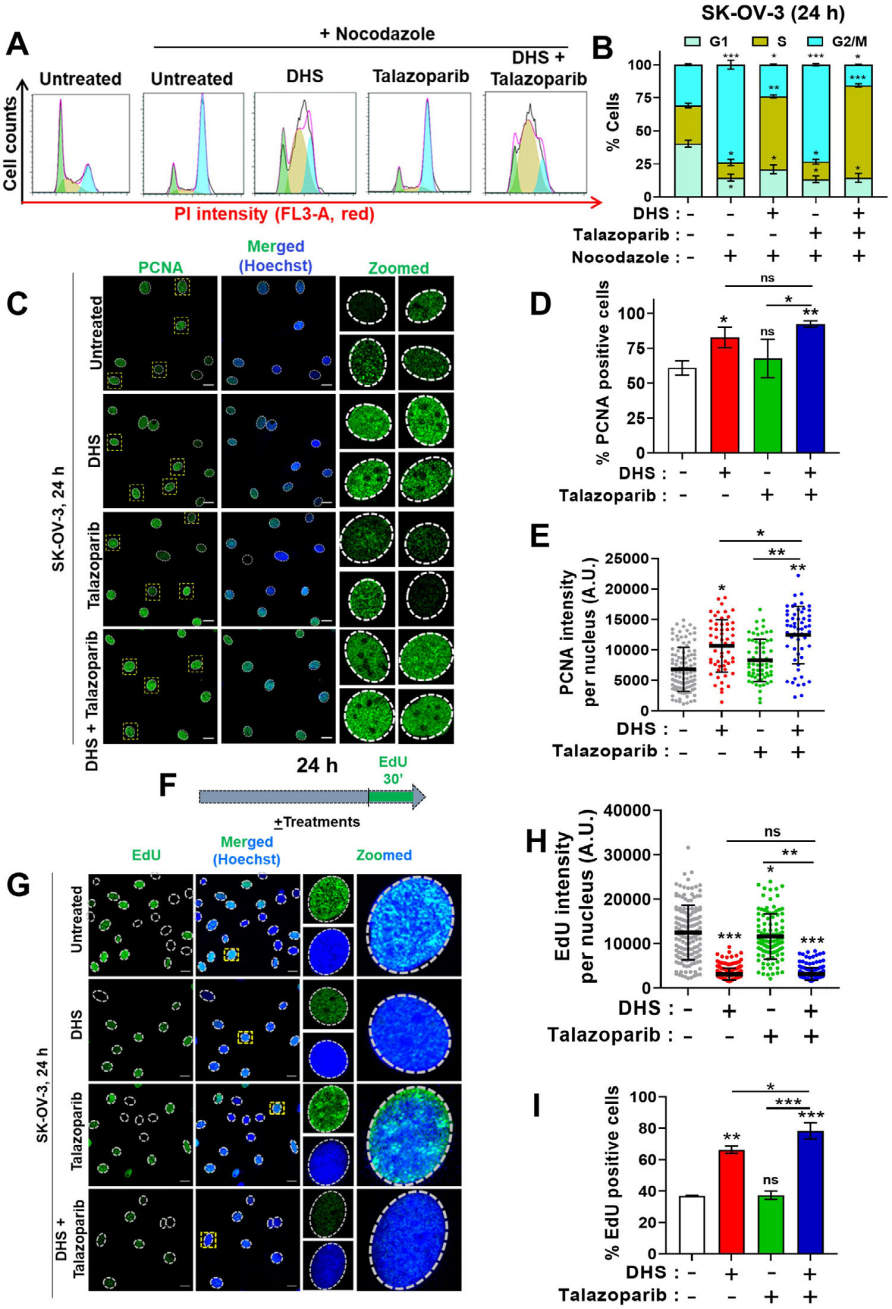
The effects of DHS and talazoparib on cell cycle and replication foci. A, B) SK‐OV‐3 cells were treated with DHS (10 µM), talazoparib (50 nM), or their combination for 24 h in the presence of nocodazole (175 ng mL^−1^) and cell cycle analysis was performed by flow cytometry. Distribution of cells in cell cycle was quantified by Flow‐Jo software and shown in B. Only the profile of the gated population of cycling cells (G1, S, or G2/M phase of the cell cycle) is shown in B. Sub‐G1 cells are gated out from the analysis. Values indicated are Mean ± S.E.M. (N = 3 biological replicates) C–E) SK‐OV‐3 cells were treated with DHS (10 µM), talazoparib (50 nM), or their combination for 24 h. PCNA foci formation was assessed by immunofluorescence microscopy. Four representative nuclei with PCNA nuclear foci in each condition were zoomed and shown in C. PCNA positive cells are indicated and the intensity of PCNA per nucleus is shown in D and E, respectively. Scale bar: 20 µm. (N = 2 biological replicates) F) Scheme of the treatment and EdU incorporation. G‐I) SK‐OV‐3 cells were treated with vehicle or DHS (10 µM) and talazoparib (50 nM) alone or their combination for 24 h. EdU was added in the last 30 min, cells were fixed and stained for EdU using click‐iT reaction kit. Scale bar: 20 µm. (*N* = 2 biological replicates). Values indicated are Mean ± S.D. ns: non‐significant, *p < 0.05, **p < 0.01, and ***p < 0.001 compared to the respective untreated group or between the groups being compared (ANOVA with Tukey post‐hoc analysis). Inter‐group comparisons are indicated.

Recently, multiple reports have suggested a role for PARP in DNA replication‐associated events and further, for DNA replication in modulating the sensitivities of cancers to PARPi treatment.^[^ ^5^, ^39^ ^]^ Apart from its role in regulating HR, stilbenes including resveratrol and DHS are known to impede DNA replication.^[^ ^25^, ^29^ ^]^ DHS inhibits RRM2 (ribonucleotide reductase regulatory subunit M2) mediated synthesis of dNTPs to affect global replication.^[^ ^29^ ^]^ However, a precise role for DHS in regulating replication machinery and its association with PARPi mediated action is not known. Flow cytometer‐based cell cycle analysis revealed that DHS treatment led to enhanced cell cycle arrest in G1/S and S phases in a concentration dependent manner (Figure S30A,B, Supporting Information). In order to further assess the impact of DHS and combination treatment on the replication driven progress through S‐phase, cell cycle analysis was carried out in the presence of nocodazole by flow cytometry. Nocodazole, a mitotic spindle poison, arrests cells in M‐phase and hence allows the analysis of cell cycle phases in a single cell cycle.^[^ ^37^ ^]^ In the presence of nocodazole, untreated control and talazoparib (50 nM) treated cells were accumulated in G2/M phase (24 h, **Figure** **4**A,B), suggesting no significant impact of talazoparib on the overall progression of cells though G1 and S‐phases of cell cycle (Figure 4A,B). Interestingly, DHS treatment led to significant accumulation of cells in S‐phase, which was further synergistically enhanced in the combination treatment (Figure 4A,B). Proliferating cell nuclear antigen (PCNA) is distributed in the cell nucleus differently at different stages, accumulates during the S phase and forms foci, and dissociates at other stages of the cell cycle.^[^ ^40^ ^]^ As shown in Figure 4C, D, talazoparib alone treatment did not change the PCNA foci patterns and % PCNA positive cells. Further, DHS alone (5 or 10 µM) and DHS *plus* talazoparib treatment led to significant increase in % PCNA positive cells (Figure 4C,D; Figure S30C–E, Supporting Information), indicating an extensive arrest in the S‐phase. Moreover, PCNA foci were extensive and higher in number in response to DHS treatment (5 or 10 µM), which were further enhanced in the combination treatment (Figure 4C,E; Figure S30D,F, Supporting Information), reflecting stabilization of more replication factories under these conditions.

In contrast, we observed that DHS (5 or 10 µM) alone induced a substantial reduction in the incorporation of ethynyl‐deoxyuridine (EdU) per nucleus as well as the size and intensity of EdU foci, suggesting severe impact of DHS on the optimal functioning of replication factories (Figure 4F–H; Figure S30G,H, Supporting Information). In combination treatment, severe impact on these parameters was not further affected at higher concentration (10 µM; Figure 4F–H) but significantly pronounced at lower concentration of DHS (5 µM; Figure S30G,H, Supporting Information). Talazoparib alone treatment had no or marginal effect on EdU incorporation, foci size and numbers per nucleus (Figure 4F–H). Strikingly, although EdU incorporation was reduced, there was a robust increase in the number of EdU positive (EdU^+^) cells after DHS alone and combination treatment (Figure 4F,G,I), suggesting that DHS may be pushing a greater number of cells into S‐phase, making them vulnerable to PARPi treatment. Of note, the effect of the combination was more prominently observed at lower DHS concentration (5 µM) in both PCNA foci formation and EdU incorporation analysis indicating an acute effect of DHS alone at higher concentrations (Figure S30D–I, Supporting Information).

Further, EdU incorporation was assessed post 24 h of DHS and/or talazoparib treatments, followed by recovery in drug‐free condition for 30 or 60 min (Figure S31A, Supporting Information). EdU incorporation was acutely reduced in response to 24 h DHS alone treatment, which was readily rescued and significantly enhanced during recovery conditions (30‐60 min) (Figure S31A–E, Supporting Information). This result suggests a transient/reversible impact of DHS alone on replication process. Talazoparib treatment and recovery conditions had no major impact on EdU incorporation. Interestingly, EdU incorporation was severely reduced in talazoparib *plus* DHS treatment, which was not rescued even after 30–60 min recovery period (Figure S31A–E, Supporting Information), indicating a severe and long‐term effect of the combination treatment on replication dynamics. The latter result could not be explained merely based on dNTP depleting effects of DHS. Of note, classical replication stress inducer, hydroxyurea (HU) is also known to reduce dNTP pools through potent inhibition of RNR enzyme. Notably, HU is reported to reduce both EdU incorporation and PCNA foci dynamics (foci intensity and numbers) significantly in the nucleus.^[^ ^41^, ^42^ ^]^ Contrarily, DHS enhances PCNA dynamics (foci intensity and numbers) while reducing EdU incorporation per nucleus (Figure 4C vs 4G). This suggests differences in the molecular mechanism of action of the two agents (HU and DHS), and that molecular mechanism of DHS go beyond RRM2 inhibition in regulating replication dynamics. In order to assess the cellular localization of DHS, we have synthesized DHS conjugated with alkyne moiety on its 4′‐OH group (Propargyl‐DHS, (E)‐4‐(4‐(prop‐2‐yn‐1‐yloxy)styryl)phenol) and characterized (Figures S32 and S33, Supporting Information). This design enables the molecule to undergo a click reaction with Alexa dye‐conjugated azide (Figure S34A, Supporting Information), analogous to the established click reaction between EdU and azide dye for localizing EdU in cells (Figure 4G). Our results demonstrate that propargyl‐DHS is efficiently internalized by cells and localized in both the cytoplasm and nucleus (Figure S34B, Supporting Information), indicating its accessibility to nuclear proteins. Together, cell cycle analysis, endogenous PCNA staining pattern, and EdU incorporation analyses indicated extensive DNA replication stress in DHS alone and combination treatment, which may be attributable to substantial accumulation of DHS in nucleus.

### 2.5 DHS *Plus* Talazoparib Severely Impedes Progress of Replication Forks

**Figure 5:**
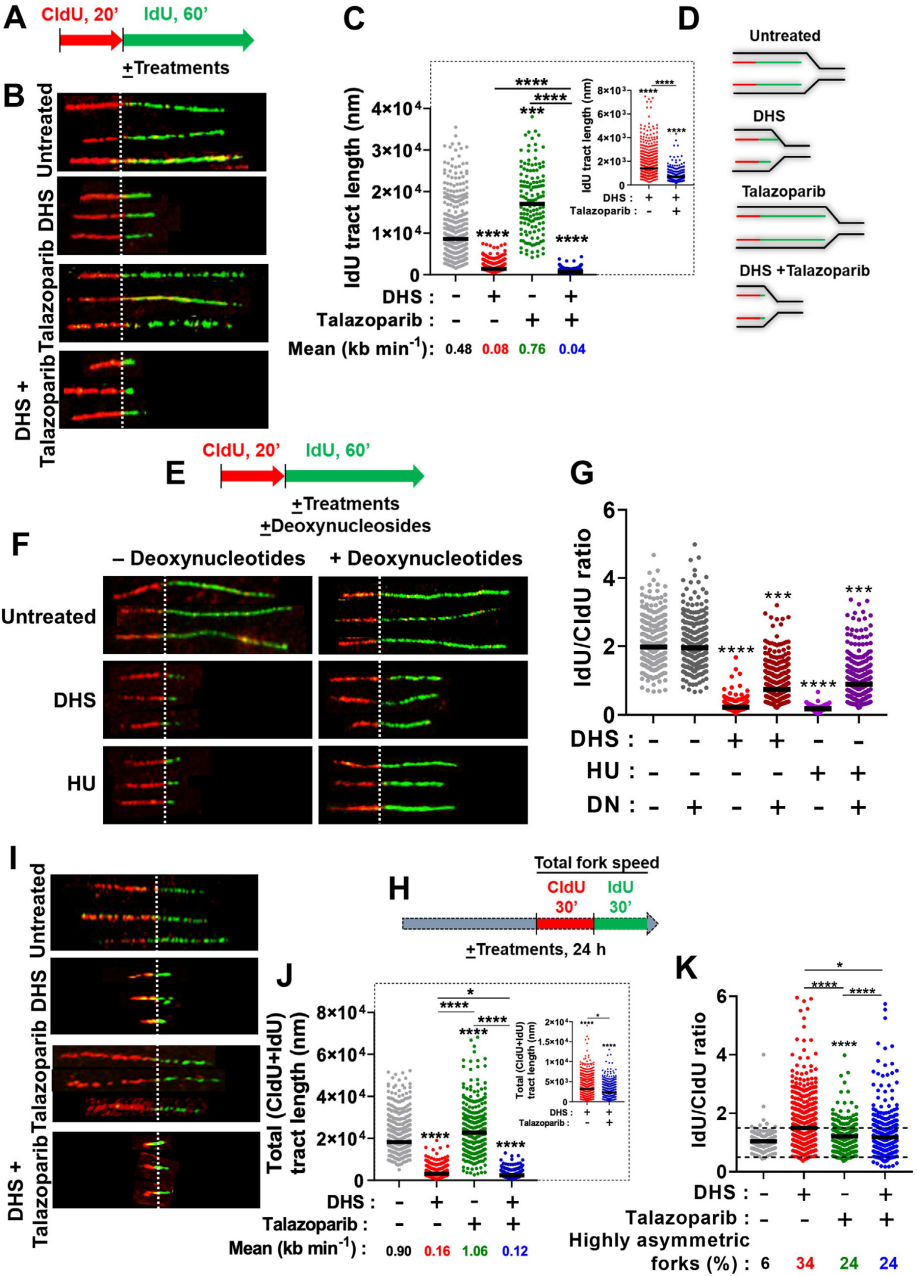
The Effects of DHS and talazoparib on replication fork progress at single molecule DNA fiber level A) Scheme of DNA fiber experiment: CldU and IdU incorporation in the absence/presence of DHS and talazoparib. SK‐OV‐3 cells were treated with CldU for 20 min, washed, treated with IdU for 60 min in the absence or presence of DHS (10 µM), talazoparib (50 nM), or their combination. B‐E) Cells after treatment, as mentioned in A, were subjected to DNA fiber analysis. Representative images of DNA fibers of different treatments are shown in B. Quantification of IdU tract length and mean fork speed in IdU fibers in fibers with both the labels (CldU and IdU) are shown in C. For inter‐group comparison between DHS vs combination treatment, an expanded Y‐axis plot has been shown in the inset. Schematic representation of replication fork progress, under different treatment conditions, are shown in D. Bar represents median. (N = 2 biological replicates). Fibers scored: Untreated (*n* = 343), DHS (*n* = 473), Talazoparib (*n* = 142), Combination (*n* = 223). ***p < 0.001, ****p < 0.0001 compared to the respective treatment in the control group or for intergroup comparisons (Kruskal‐Wallis test with Dunn's post‐hoc analysis). Fork speed was calculated by using conversion factor 2.59 kb µm^−1^ for stretched fiber as per previous report.^[^ ^61^ ^]^ (E) Scheme of DNA fiber experiment: CldU and IdU incorporation in the absence/presence of DHS, HU and deoxynucleosides (DN). SK‐OV‐3 cells were treated with CldU for 20 min, washed, treated with IdU for 60 min in the absence or presence of DHS (10 µM) or HU (500 µM) in one set. In a parallel set, a cocktail of all four deoxynucleosides were included during IdU and other treatments as shown. F, G) Cells after treatment, as mentioned in E, were subjected to DNA fiber analysis. Representative images of DNA fibers of different treatments are shown in F. Quantification of IdU/CldU tract length in fibers with both the labels (CldU and IdU) are shown in G. Bar represents median. (*N* = 2 biological replicates). Fibers scored: Untreated (*n* = 242), Untreated + DN (*n* = 285), DHS (n = 413), DHS + DN (*n* = 607), HU (n = 106), HU + DN (*n* = 291). ***p < 0.001, ****p < 0.0001 compared to the respective treatment in the control group (Kruskal‐Wallis test with Dunn's post‐hoc analysis). H) Scheme of DNA fiber experiment: CldU and IdU incorporation in the absence/presence of DHS and talazoparib. SK‐OV‐3 cells were treated with DHS (10 µM), talazoparib (50 nM), or their combination for 24 h. For last 1 h of treatment, CldU was added for 30 min, cells were washed and treated with IdU for 30 min in the absence or presence of respective treatment. I‐K) Cells after treatments, as mentioned in H, were subjected to DNA fiber analysis. Representative images of DNA fibers of different treatments are shown in I. Quantification of total tract length and fork speed in fibers with both the labels (CldU and IdU) are shown in J. For inter‐group comparison between DHS vs combination treatment, an expanded Y axis plot has been shown in the inset. Asymmetry analysis (IdU/CldU) of the replicating fibers were also carried out and shown in K. Bar represents median. (*N* = 3 biological replicates). Fibers scored: Untreated (*n* = 484), DHS (*n* = 792), Talazoparib (*n* = 272), Combination (*n* = 605). *p < 0.05, ****p < 0.0001 compared to the respective treatment in the control group or for intergroup comparisons (Kruskal‐Wallis test with Dunn's post‐hoc analysis). Fork speed was calculated by using conversion factor 2.59 kb µm^−1^ for stretched fiber.

Recent reports have unraveled modulation of replication fork dynamics as prominent mechanistic aspects of PARPi action.^[^ ^5^, ^39^ ^]^ Hence, efficient targeting of the latter process may enhance the therapeutic efficacy of PARPis against HR‐proficient cancers. In order to assess the precise role of DHS and/or talazoparib in replication dynamics, single molecule DNA fibers labelled with CldU and IdU were analyzed.^[^ ^43^ ^]^ Initially, we found that DHS severely reduced replication fork progress, and fork speed in a concentration dependent manner (1‐10 µM; Figure S35A–D, Supporting Information). Interestingly, the effect of DHS is quite severe and several folds higher than resveratrol, the latter is known to inhibit replication fork progress (Figure S35A–D, Supporting Information).^[^ ^25^ ^]^ Of note, IdU treatment was extended for relatively longer time (60 min) in the above experiments as the IdU‐tracts were barely detectable at shorter time point (30 min; data not shown). Further, cells were treated with CldU (20 min) and IdU (60 min) in the absence or presence of talazoparib and/or DHS (**Figure** **5**A). In corroboration with previous reports with PARPi,^[^ ^43^, ^44^ ^]^ DNA fiber lengths were significantly enhanced in the presence of PARPi, talazoparib (Figure 5A–C; See the inset for DHS vs combination treatment). Interestingly, DHS treatment led to severe impediment on fork progress and reduced fork speed, which was further affected severely in combination treatment (Figure 5A–D). The impact of talazoparib and/or DHS on IdU labelled (green) forks is as depicted in Figure 5D. Intriguingly, external supplementation of deoxynucleosides cocktail (DN) significantly rescued fork progress, albeit partially, in response to DHS and HU treatment (Figure 5E–G), suggesting that both DHS and HU majorly target depletion of dNTPs pool for reducing fork progress. Strikingly, fork progress was not rescued upon external supplementation of nucleosides in response to combination treatment of talazoparib *plus* DHS (data not shown), suggesting a severe damage of replication forks by combination treatment. Moreover, a similar impact of DHS, talazoparib and combination treatment on fork progress, speed and fork symmetry was observed following long duration exposure (24 h), when DNA fibers were analyzed after labeling with CldU and IdU for 30 min each (Figure 5H–K). We also labelled the forks with the CldU and IdU for 60 min each post 22 h exposure to the treatment conditions to check if the fork progression is maintained/steady under different treatment conditions. Interestingly, we found that under untreated or talazoparib treatment, the forks elongated proportionately as with 30 min labelling experiment. But the DHS alone or DHS *plus* talazoparib treated cells did not show a proportional increase in the tract lengths suggesting a protracted fork progression dysfunction (Figure S35E–G, Supporting Information).

### 2.6 DHS Enhances Replication Gaps in Response to PARP Inhibition

**Figure 6:**
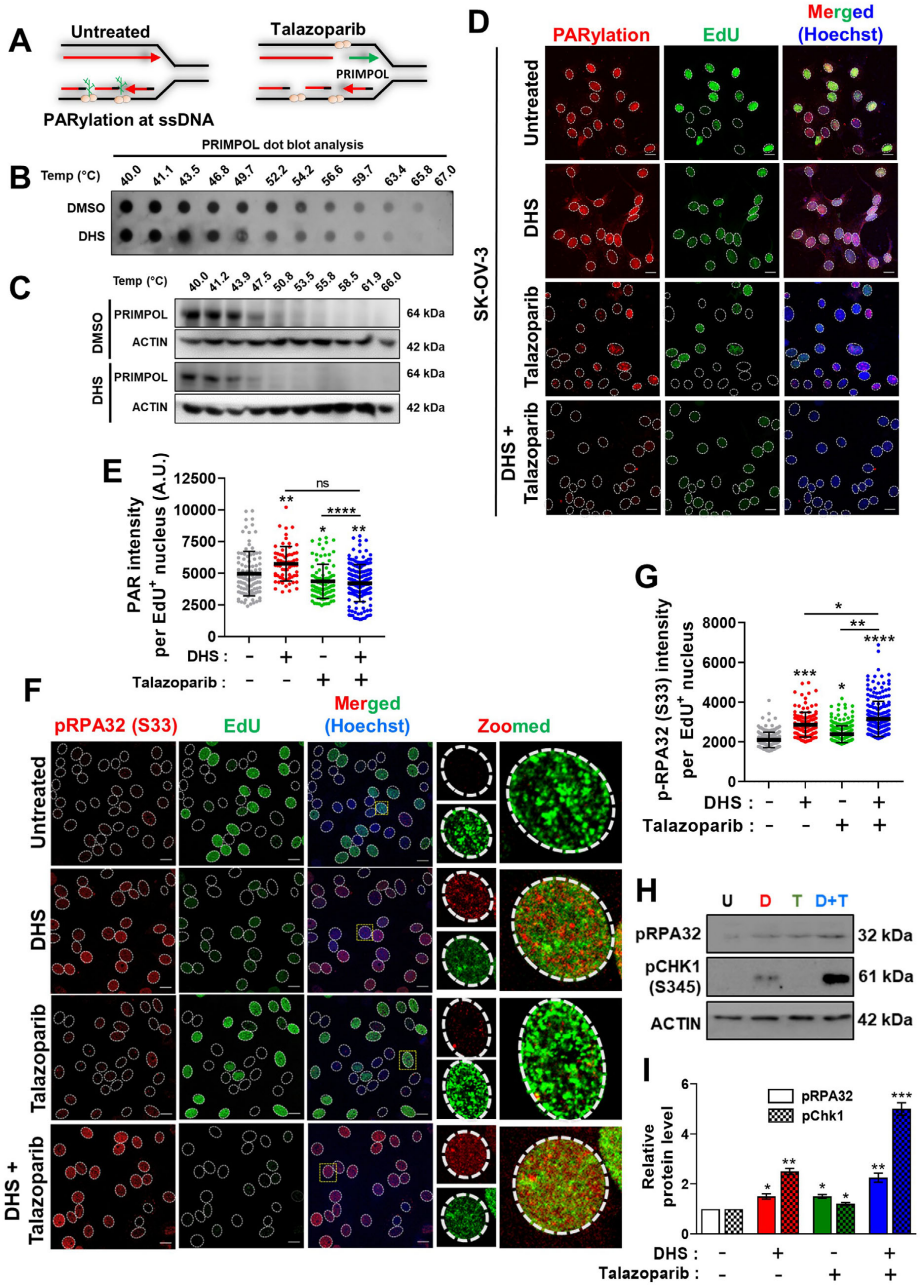
The effect of DHS and talazoparib on PRIMPOL, PARylation and ssDNA gaps in replicating cells. A) Scheme showing the role of PRIMPOL, PARylation and ssDNA gaps in untreated and talazoparib treated cells. B, C) Cells were treated with DHS (10 µM) or DMSO for 2 h. and subjected to thermal denaturation (See Experimental section). PRIMPOL was assessed by dot blot and western blotting. D) PARylation analysis: SK‐OV‐3 cells were treated with vehicle, DHS (10 µM) and talazoparib (50 nM) alone or their combination for 24 h. EdU was added for last 30 min of the treatments. PARylation (immunofluorescence) and EdU (by click reaction) were analyzed and shown in D. Scale bar: 20 µm. Quantification for intensity of PARylation in EdU^+^ cells is shown in E. F, G) Analysis for RPA32 phosphorylation. SK‐OV‐3 cells were treated with vehicle, DHS (10 µM) and talazoparib (50 nM) alone or their combination for 24 h. EdU was added for last 30 min of the treatment duration. RPA2 phosphorylation (S33) (immunofluorescence) and EdU (click reaction) were analyzed and shown in F. Quantifications for intensity of RPA2 phosphorylation in replicating cells (EdU^+^ cells) is shown in G. H, I) Analysis for RPA32 and CHK1 phosphorylation. SK‐OV‐3 cells were treated with vehicle (U), DHS (D, 10 µM) and talazoparib (T, 50 nM) alone or their combination (D+T) for 24 h and cell extract were prepared to analyze pRPA32(S33) and pCHK1(S345) phosphorylation by western blotting. Quantification of western blotting was shown in I. N = 3 biological replicates. Values indicated are Mean ± S.D. ns: non‐significant, *p < 0.05, **p < 0.01, ***p < 0.001 and ****p < 0.0001 compared to the respective untreated group or between the groups being compared (ANOVA with Tukey post‐hoc analysis). Inter‐group comparisons are indicated.

Upon PARPi treatment, both BRCA1‐WT and mutant cells show molecular responses to replication stress. To inhibit pathological reversed fork degradation by MRE11, repriming of replication forks is initiated by PRIMPOL^[^ ^44^ ^]^ (**Figure** **6**A). Also, it is to be noted that PARP1 is essential for unligated Okazaki fragments processing (OFP) through PARylation (poly‐ADP‐ribosylation)‐mediated recruitment of XRCC1‐LIG3^[^ ^17^, ^18^ ^]^ (Figure 6A), which otherwise leads to pathological genome breakage in response to PARPi.^[^ ^39^ ^]^ Both the above responses generate ssDNA gaps behind the forks (Figure 6A). Since, DHS impedes replication fork progress when combined with PARPi, we sought to know whether DHS inhibits PRIMPOL and/or OFP mediated fork progress, apart from its conventional role of depleting dNTP pool. Previously, it has been shown that resveratrol and other stilbene molecules have the propensity to inhibit different DNA polymerases.^[^ ^45^ ^]^ In order to assess if DHS can bind directly to PRIMPOL, cellular thermal shift assay (CETSA) was performed.^[^ ^29^, ^46^ ^]^ In this assay, protein bound with ligands are relatively thermally stable and recover in soluble fraction of cell lysate in response to temperatures higher than ambient temperature.^[^ ^46^ ^]^ The stability of the specific ligand‐bound‐protein in cell lysate can be assessed by dot/Western blotting. Our results revealed that treatment of SK‐OV‐3 cells with DHS, followed by exposure to a gradient of temperatures (40‐67 °C), a marginal/no increase was observed in the thermal stability related release of PRIMPOL in soluble fraction of cell lysates, as assessed by both dot and Western blotting (Figure 6B,C). This result suggested that DHS may not be directly targeting PRIMPOL to impede fork progress under replication stress (Figure 6A–C). Further, the effect of DHS on the generation of ssDNA gaps and OFP was assessed. Previously, it has been shown that OFP defect is associated with enhanced PARylation in S‐phase/replicating cells, consistent with the notion that lagging strand synthesis/processing requires PARP1‐mediated PARylation.^[^ ^5^ ^]^ Here, we found that DHS alone treatment led to significant increase in PARylation in replicating (EdU^+^) cells (Figure 6D,E), which may be attributable to requirement of PARP1‐mediated PARylation at the stressed lagging strands in response to DHS. Moreover, as expected, PARylation was significantly reduced in EdU^+^ cells of talazoparib and combination treatments (Figure 6D,E). Since both DHS treatment and PARPi potentially impact OFP, combination treatment may have a synergistic impact on the OFP, leading to enhanced unrepaired replication gaps (ssDNA). In corroboration with this hypothesis, DHS treatment led to significant enhancement in RPA32 phosphorylation (pRPA32 (S33)), a marker of ssDNA, in EdU^+^ cells, which was further enhanced in talazoparib *plus* DHS treated cells (Figure 6F,G). A similar result for phosphorylation of RPA32 and CHK1, as markers of ssDNA/replication gaps, was also observed in Western blotting analysis (Figure 6H,I). A synergistic enhancement in pRPA32 phosphorylation in replicating cells (EdU^+^ cells) upon treatment with DHS and talazoparib was even observed in lower concentration of DHS in SK‐OV‐3 cells (Figure S36A,B, Supporting Information) and other BRCA1/2 wild‐type ovarian cancer cell lines PA‐1 (Figure S37A–C, Supporting Information), OAW42(Figure S38A–C, Supporting Information) and A2780 (Figure S39A–C, Supporting Information). Previous studies have demonstrated that talazoparib can able to trap PARP on chromatin, leading to induction of replication stress in cancer cells.^[^ ^47^, ^48^ ^]^ In this regard, our result showed that talazoparib induced PARP trapping was further enhanced in the presence of DHS (Figure S40, Supporting Information), suggesting higher replication stress (Figure 6F–I) in combination treatment may be partly attributable to enhanced PARP trapping on chromatin. Collectively, our results suggested a role for DHS in enhancing PARylation at lagging strand synthesis, trapping of talazoparib induced PARP on chromatin and ssDNA gaps in replicating cells.

### 2.7 Inhibition of PARylation at Nascent DNA leads to Fork Collapse in Response to DHS

**Figure 7:**
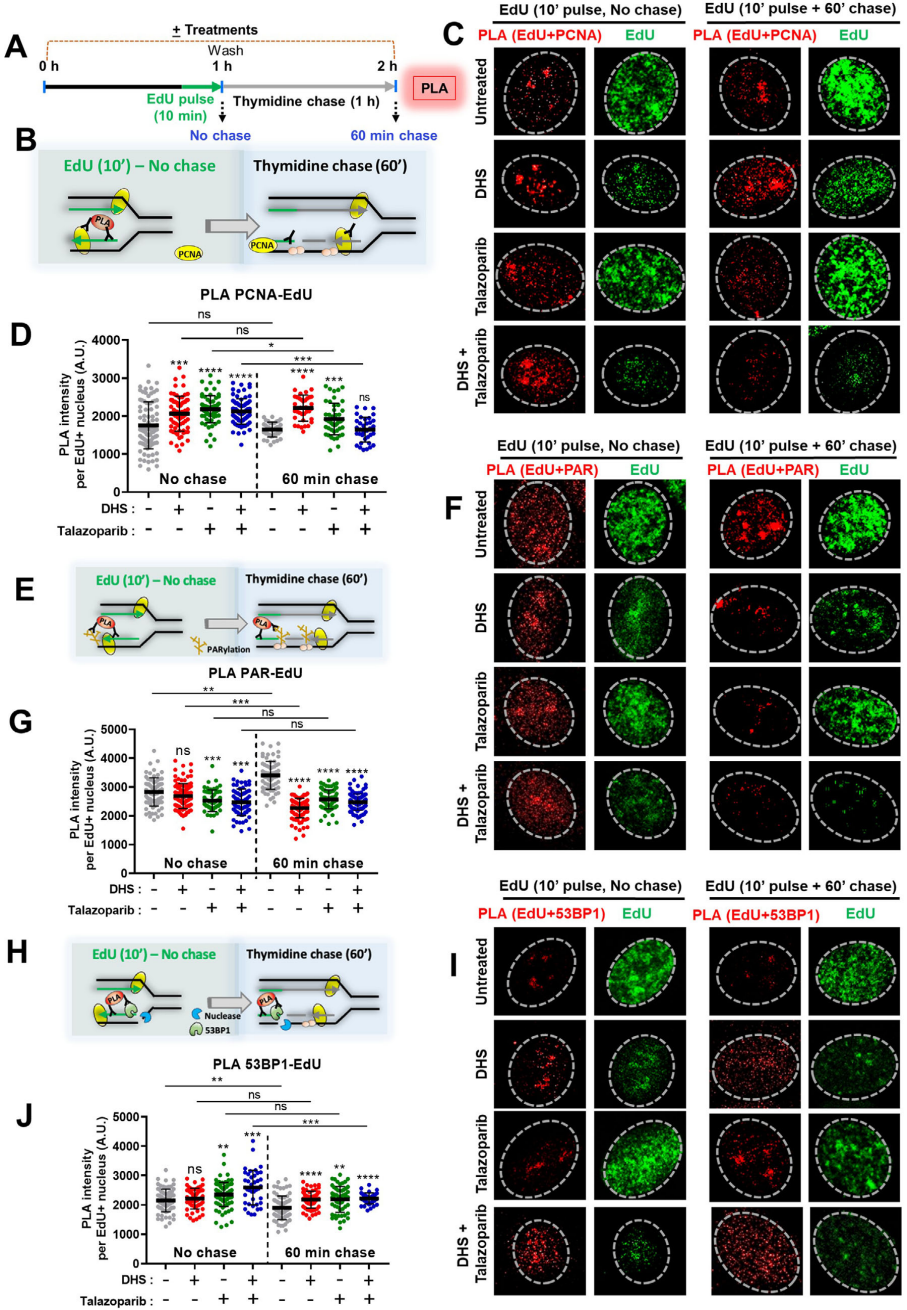
Interaction of nascent DNA (EdU) tracts with PCNA, PARylation and 53BP1 behind the forks in response to DHS and talazoparib. A) Scheme of treatment for PLA analysis. One set of SK‐OV‐3 cells were treated with DHS and talazoparib alone or their combination for 60 min. For last 10 min EdU was added to label the nascent forks, washed and fixed to carry out PLA. Second set of cells were treated as above, washed to remove EdU and chased for 60 min in the presence of thymidine and the respective treatments. After chase, the cells were washed and fixed to perform PLA. B) Schematic to show the interaction of nascent DNA tracts (EdU) with PCNA during ongoing replication and after a 60 min chase. C, D) Cells were treated as mentioned above, microscopy analysis of PLA signals for interaction of nascent DNA tracts (EdU) with PCNA during ongoing replication and after a 60 min chase were performed. Representative images for PLA in EdU^+^ cells are shown in C. Quantifications of the PLA signals are shown in D. E‐G) SK‐OV‐3 cell were treated as mentioned above in A. Schematic to show the interaction of nascent DNA tracts (EdU) with PARylation during ongoing replication and after a 60 min chase is shown in E. Microscopy analysis of PLA signals for the interaction of nascent DNA tracts (EdU) with PARylation during ongoing replication and after a 60 min chase were performed and representative images shown in F. Quantifications of the PLA signals are shown in G. H‐J) SK‐OV‐3 cell were treated as mentioned above in A. Schematic to show the interaction of nascent DNA tracts (EdU) with 53BP1 during ongoing replication and after a 60 min chase is shown in H. Microscopy analysis of PLA signals for interaction of nascent DNA tracts (EdU) with 53BP1 during ongoing replication and after a 60 min chase were performed and representative images shown in I. Quantifications of the PLA signals are shown in J. (N = 2 biological replicates) Values indicated are mean ± S.D. ns: non‐significant, *p < 0.05, **p < 0.01, *p < 0.001, ****p < 0.0001 compared to the respective treatment in the control group or for intergroup comparisons (ANOVA with Tukey post‐hoc analysis).

To confirm that these events (ssDNA gaps and PARylation), plausibly localized behind the replication forks, the proximity of EdU‐labelled tracts with PCNA (replication machinery on ongoing replication fork) was assessed by proximity ligation assay (PLA), soon after EdU pulse (10 min) and follow up thymidine chase for 60 min (**Figure** **7**A,B). Previously, the PLA‐based assay has been employed to detect protein binding to EdU‐labeled nascent DNA.^[^ ^39^, ^49^ ^]^ With 10 min EdU pulse, PLA signals were significantly enhanced in DHS and talazoparib alone and combination treatments compared to untreated control cells, suggesting higher interaction of PCNA machinery with nascent forks (Figure 7B–D). With 60 min chase after 10 min EdU pulse, the PLA signals for PCNA‐EdU were further enhanced in DHS while it was reduced in talazoparib treated cells (compare chase *vs* no chase, Figure 7D). This result suggests that, replication machinery moved away from the nascent EdU‐tracts with time in talazoparib treatment while the residence time of the PCNA machinery at nascent EdU‐tract is longer for DHS treatment during 60 min chase. Unexpectedly, we found that PLA signal was drastically reduced in combination treatment during chase, indicating parting away of PCNA machinery from the EdU‐nascent tract (Compare chase vs no chase, Figure 7D). Considering a slower progress of replication forks in combination treatment (Figure 5A–C), it is plausible that low PLA signals might stem from a higher frequency of replication forks collapsing in the combination treatment.

Next, proximity of nascent EdU tract with PAR was assessed (Figure 7A,E). In untreated cells, PLA signals were enhanced during 60 min chase (Figure 7A,F,G, compare untreated in chase *vs* no chase), suggesting that synthesized nascent tracts were continuously PARylated behind the forks even in the unperturbed cells. PLA signals, during chase, were reduced in DHS treated cells, while these were unaffected in talazoparib alone and combination treatments (Figure 7A,E–G, no chase vs chase), indicating that Okazaki fragments were not accessible to PARylation behind the forks in DHS treatment while PARylation was reduced in talazoparib and combination treatment at later time point (Figure 7E–G). From these results it appears that DHS enhances the residence time of PCNA (replication machinery) with nascent DNA (Figure 7D,E), leading to reduced rate of PARylation at nascent forks (Figure 7F,G), which may trigger PARylation at later time points for OFP. This may eventually enhance PARylation‐mediated OFP in S‐phase cells in DHS treatment (Figure 6D,E). In contrast, suppression of PARylation in combination treatment may have severe impact on OFP, which may undergo fork collapse. In this respect, our PLA analysis with nascent EdU tracts with 53BP1, a marker for fork collapse^[^ ^50^ ^]^ showed a rapid rise in PLA signal within 10 min EdU pulse in cells with combination treatment than untreated control cells (Figure 7A,H–J). During 60 min chase, PLA signals were slowly enhanced in individual DHS or talazoparib treatment and combination treatment than untreated control (Figure 7A,H–J). It might be plausible that fork cleavage was far apart from the EdU labeled nascent DNA and hence not amenable for PLA interaction, leading to no further enhancement of PLA signals in combination treatment during chase period (Figure 7A,H–J). Hence, analysis of total DSBs in replicating cells (S‐phase, EdU^+^ cells) may provide better insights into the synergistic mechanism of combination treatment.

### 2.8 MRE11 Converts Stalled Forks into Lethal Collapsed Forks in Response to Talazoparib *Plus* DHS Treatment

**Figure 8:**
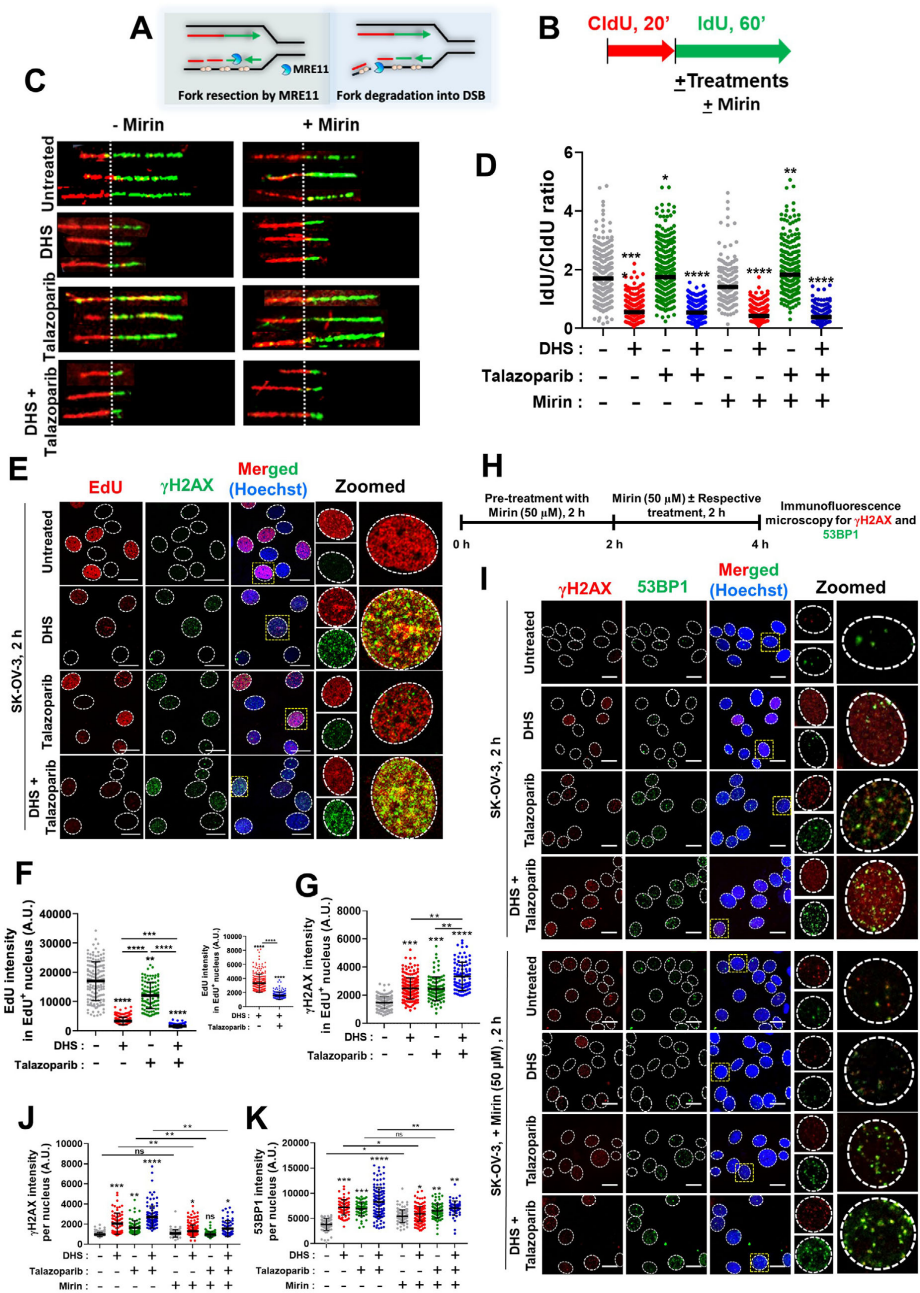
Role of MRE11 in replication fork processing and generation of DSBs in response to DHS and talazoparib. A) Scheme showing the role of MRE11 in OFP and converting ssDNA gaps into DSBs B) Scheme of DNA fiber experiment: SK‐OV‐3 cells were treated with CldU for 20 min, washed, treated with IdU for 60 min in the absence or presence of DHS (10 µM), talazoparib (50 nM), or their combination. In a parallel experiment, IdU and other treatments were carried out in the presence of mirin (50 µM, MRE11 inhibitor) C, D) Cells after treatments, as mentioned in B, were subjected to DNA fiber analysis. Representative images of DNA fibers of different treatments are shown in C. Quantification of IdU/CldU ratios in fibers with both the labels (CldU+IdU) are shown in D. Bar represents median. (*N* = 3 biological replicates). Fibers scored: Untreated (*n* = 264), DHS (*n* = 462), Talazoparib (*n* = 349), Combination (*n* = 382). Untreated + Mirin (*n* = 153), DHS + Mirin (*n* = 372), Talazoparib + Mirin (*n* = 239), Combination + Mirin (*n* = 355). E–G) SK‐OV‐3 cells were treated with DHS (10 µM), talazoparib (50 nM), or their combination for 2 h. During last 30 min of the treatment duration, EdU was added to label the ongoing replication foci, washed, fixed and analyzed for EdU (click reaction) and γH2AX (immunofluorescence) and representative images were shown in E. Note that the Intensity of the zoomed image of EdU stained nucleus in E in DHS and combination treatment conditions have been enhanced to show colocalization more clearly. Intensities of EdU and γH2AX per replicating EdU^+^ nucleus were measured and shown in F and G. For inter comparison between DHS vs combination treatment, an expanded Y axis plot has been shown in the inset in F (*N* = 3 biological replicates). H–K) Effect of MRE11 on γH2AX and 53BP1 formation. SK‐OV‐3 cells were treated with DHS (10 µM), talazoparib (50 nM), or their combination for 2 h in the absence or presence of mirin (50 µM) as per the scheme in H. Cells were fixed and analyzed for γH2AX and 53BP1 signals by immunofluorescence microscopy and representative images were shown in I. Intensities of γH2AX and 53BP1 per nucleus were measured and shown in J and K respectively. (*N* = 3 biological replicates). Values indicated are mean ± S.D. ns: non‐significant, *p < 0.05, **p < 0.01, ***p < 0.001, ****p < 0.0001 compared to the respective treatment in the control group or for intergroup comparisons (ANOVA with Tukey post‐hoc analysis).

Recent reports advocate (i) Fork degradation by MRE11,^[^ ^51^ ^]^ (ii) an inhibitory role of MRE11 on fork progression even in BRCA‐proficient cancers^[^ ^44^ ^]^ and (iii) uncontrolled exonuclease activity of MRE11 expands replication gaps at unprocessed OF, which eventually lead to lethal genomic breaks by the endonuclease activity of MRE11^[^ ^52^ ^]^ (**Figure** **8**A). We sought to know if MRE11 has any impact on nascent DNA, leading to shorter tract length with extensive replication gaps in DHS *plus* talazoparib treatments (Figure 8A). In this regard, our DNA fiber analysis showed that pharmacological inhibition of MRE11 by mirin had no significant impact on replication tract length in respective untreated, DHS and talazoparib alone and combination treatments (Figure 8B–D), suggesting that degradation/inhibitory property of MRE11 may not be associated significantly with nascent forks in these treatments. Intriguingly, treatment of talazoparib *plus* DHS for 2 h led to formation of γH2AX (DSBs) extensively, a substantial fraction of which colocalize with nascent replication foci, labelled with EdU for last 30 min of treatment (Figure 8E–G). The amount of γH2AX was significantly lower in individual treatment of talazoparib or DHS alone (Figure 8E–G), suggesting a synergistic effect of talazoparib and DHS in inducing the collapse of stressed forks. In corroboration with this result, we observed a similar enhancement of 53BP1 foci, another marker of DSBs, and its colocalization with γH2AX foci in response to combination treatment *vis‐à‐vis* individual treatment of talazoparib or DHS (Figure 8H–K). Interestingly, pharmacological inhibition of MRE11, by mirin, significantly reduced fork collapse‐associated γH2AX and 53BP1 in individual and combination treatments of talazoparib and DHS, suggesting a critical role for MRE11 in converting stressed forks into lethal collapsed forks (Figure 8H–K). This phenomenon was also evident in combination of talazoparib with lower DHS concentration (5 µM; Figure S41A–C, Supporting Information). Together, our results indicated that DHS impedes fork progression majorly by reducing dNTP pools and enhancing PARylation behind the forks among other plausible mechanisms. Inhibition of PARylation events, intuitively at the nascent Okazaki fragments owing to the enhanced accumulation of cells in S‐phase, triggers MRE11‐dependent processing and fork collapse.

In addition to above effects at early time point (2 h), we also observed significantly higher accumulation of DSBs (γH2AX and 53BP1 foci) in replicating (EdU^+^) cells in response to combination treatments at 24 h than talazoparib and DHS treatment alone Figures S42A,B; S43A,B; S44A–C, Supporting Information). Further, neutral comet assay results also showed significantly higher DSBs in combination treatments (Figure S44D,E, Supporting Information), confirming synergistic effects of converting stalled replication forks into lethal DSBs. The synergistic effects of combination treatment in inducing DSBs in replicating cells were also observed in multiple ovarian cancer cell lines such as PA‐1 (Figure S45A,B, Supporting Information), OAW42 (Figure S46A,B, Supporting Information) and A2780 (Figure S47A,B, Supporting Information) cells. Of note, this synergistic effect of talazoparib *plus* DHS was not limited to ovarian cancers, as similar enhancement in DSBs while EdU incorporation is simultaneously suppressed in replicating cells were also observed in MCF‐7 (breast carcinoma; Figure S48A–C, Supporting Information), IMR32 (neuroblastoma; Figure S49A–C, Supporting Information) and MIA‐PaCa‐2 (pancreatic ductal adenocarcinoma; Figure S50A–C, Supporting Information), suggesting this combination treatment may also be extended to other cancer types, where PARP inhibitor therapy is extensively pursued.

### 2.9 DHS *plus* Talazoparib Treatment Reduces Tumor Burden in Pre‐Clinical Mice Model

**Figure 9:**
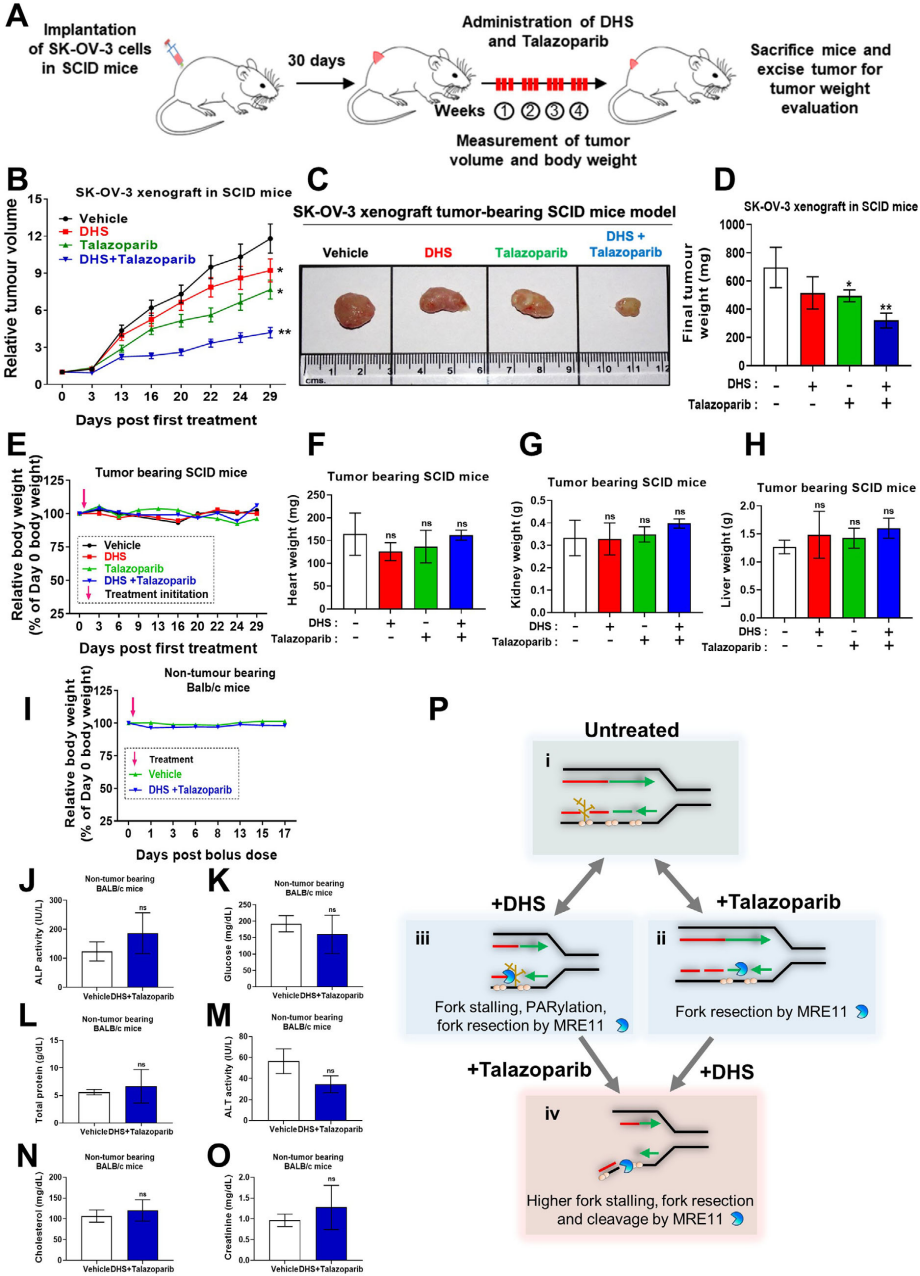
Combination of DHS and talazoparib reduces tumor volume with no apparent toxicities in pre‐clinical mouse models A) Scheme showing treatment regimen in mice: Immunodeficient SCID mice bearing SK‐OV‐3 tumor xenografts were administered vehicle, DHS alone (50 mg/kg), talazoparib alone (1 mg kg^−1^), or their combination by oral gavage on every alternate day for 4 weeks. (*n* = 5 mice per group) B) During the treatments, dimensions of the tumor were measured on the indicated days and the tumor volumes assessed and shown in B. C, D) After 4 weeks of respective treatment, mice were sacrificed and tumors were excised and weighed. Representative images of tumors are shown in C, and the final tumor weight quantification is shown in D. Values indicated are mean ± S.E.M. E–H) Relative body weights of the animals and the weights of vital organs of the tumor bearing SCID mice were noted and plotted. (*n* = 5 mice per group) I) Non‐tumor bearing mice were treated with the single bolus dose of the combination of DHS and talazoparib dosage (5x of therapeutic dose, i.e., 250 mg kg^−1^ DHS and 5 mg kg^−1^ talazoparib). Relative body weights of the animals treated with vehicle and DHS *plus* talazoparib is shown. (*n* = 5 mice per group). J‐O) Non‐tumor bearing mice were treated as shown in I and the serum biochemical parameters for the liver and kidney functions and pathologies were assessed post 24 h and shown. (*n* = 5 mice per group) Values indicated are mean ± S.D. ns: non‐significant, **p < 0.01 and ***p < 0.001 compared to vehicle treated group. P) Model depicting the synergistic role of PARPi and DHS in converting ssDNA gaps into lethal DSBs. (i) PARP1 mediated PARylation is necessary for recruitment of downstream single‐strand break repair factors to support backup OF processing under compromised BRCA‐RAD51 regulated canonical pathway.^[^ ^5^, ^55^ ^]^ (ii) Inhibition of PARP1 compromises OF processing, which contributes to enhanced ssDNA gaps. (iii) DHS potently downregulates dNTP pools and RAD51 expression, leading to enhancement in ssDNA gaps and PARylation. This in turn causes acute inhibition of replication fork progress, speed and fork symmetry. (iv) In combination treatment of talazoparib *plus* DHS, inhibition of PARylation‐mediated OF processing leads to robust enhancement of ssDNA gaps and fork impediment. Subsequently, ssDNA replication gaps are converted to lethal DSBs by MRE11.

After establishing the synergistic efficacy of DHS *plus* talazoparib in ovarian and other cancer cell lines, the synergistic efficacy of combination treatment was assessed in vivo preclinical mouse models. Therapeutic doses of DHS and talazoparib used in our study were based on previously established reports.^[^ ^15^, ^27^, ^28^ ^]^ SCID mice bearing subcutaneous ovarian tumor (SK‐OV‐3) were given vehicle, DHS, talazoparib and combination regimen through oral gavage (12 doses in 4 weeks; see Experimental section) (**Figure** **9**A). Our results revealed that combination treatment of talazoparib *plus* DHS was significantly effective in reducing ovarian tumor growth, even after initial few doses to final dose, compared to individual treatments (Figure 9A–D). Furthermore, the analysis of relative weight of whole body and vital internal organs of tumor bearing SCID mice showed that the combination of talazoparib *plus* DHS was well‐tolerated and comparable to the control and individual treatment groups (Figure 9E–H). Moreover, our preclinical mouse model analysis showed that a single bolus dose, 5 times higher than therapeutic dose, of talazoparib and DHS was also well tolerated (Figure 9I). In corroboration with this result, our biochemical parameters for specific organ toxicity *viz*. ALP (alkaline phosphatase), ALT (alanine aminotransferase), glucose, total proteins in blood, cholesterol, creatinine showed no organ toxicity in response to a bolus dose of combination treatment (Figure 9J–O). Together, we found that the combination of DHS and talazoparib was effective in reducing ovarian tumor burden in xenograft SCID mice models. It showed high tolerability with low/no toxicity in the tumor bearing mouse model as well as in the non‐tumor bearing Balb/c mice.

## 3 Discussion

Irrespective of BRCA mutation status, PARP inhibitors are effective against all tumors, although therapeutic efficacy is higher in patients with BRCA mutations/HRD, as observed in clinical trials.^[^ ^12^ ^]^ Recently, survival benefit was observed in patients with epithelial ovarian carcinoma treated with PARPi, veliparib, and paclitaxel and carboplatin, followed by veliparib compared with chemotherapy alone in the Phase III VELIA trial.^[^ ^53^ ^]^ Hence, there is a need of understanding the molecular mechanism of PARP inhibitors to enhance its efficacy against HRP cancers through combination therapy.

Considering key role of PARP in maintaining replication fork dynamics, combination treatment of PARPi with ATRi and CHK1i is reported to be effective against chemo‐resistant HRP ovarian cancers.^[^ ^19^, ^20^, ^21^ ^]^ Kim *et al.* have shown that ATRi‐PARPi synergistically enhances replication fork stalling, generation of asymmetric replication forks and fork collapse, leading to sensitivity of PARPi resistant ovarian cancers, including *BRCA1/2* reversions and CCNE1‐amplified cancers.^[^ ^20^, ^21^ ^]^ In another study, Parmar *et al* have shown that CHK1i (Prexasertib) monotherapy effectively sensitized PARPi‐resistant HRP ovarian cancers in preclinical PDX models, especially through restoring replication fork degradation.^[^ ^19^ ^]^ In this regard, our investigation showed that DHS, a small natural stilbene molecule from *Yucca periculosa* plant, synergistically reduced clonogenic growth and enhanced apoptosis in two different HRP ovarian cancers, and breast cancer in response to PARPi treatment (Figure 2). Mechanistically, DHS synergistically impeded the progress of S‐phase cells, induced early S‐phase arrest, and severely affected EdU incorporation and replication dynamics in response to PARPi treatment (Figure 4). Moreover, the combination treatment led to acute fork stalling with extensively reduced fork speed compared to individual treatments of DHS and talazoparib. Chen *et al* reported the effect of DHS‐mediated inhibition of RRM2 leading to depletion of dNTP pool.^[^ ^29^ ^]^ Hence, DHS mediated acute stalling of replication forks may be majorly ascribed to reduced dNTP pool. Interestingly, our further experiments revealed that (i) external supplementation of deoxynucleosides only partially rescued fork stalling (Figure 5E–G) and (ii) fork progress was highly asymmetric in DHS treatment (Figure 5K), suggesting forks are frequently encountering other replication‐associated issues, in addition to depleted dNTP pool in response to DHS treatment.

During replication, in each S‐phase of human cell, ∼50 million Okazaki fragments (OF) are formed. PARP1 ensures efficient Okazaki fragment processing (OFP) and ligation to maintain nascent fork integrity.^[^ ^17^, ^18^ ^]^ Failure of even a small fraction of OFs leads to accumulation of single‐strand gaps and SSBs in S‐phase. Recently, several reports have shown that accumulation of single‐strand gaps and their eventual conversion to DSBs behind the replication forks determine PARPi sensitivity in preclinical clinical scenario.^[^ ^54^ ^]^ Saldanha *et al* have shown that inadequate PARP1 activity and the loss of TIM induces uncoupling of synthesis of two nascent strands, leading to generation of fork asymmetry, frequent fork stalling and ssDNA gaps behind the forks.^[^ ^55^ ^]^ Extensive lagging strand‐associated ssDNA gaps have been linked to cytotoxicity and sensitivity to PARPi.^[^ ^54^, ^55^ ^]^ In the current investigation, DHS treatment induces robust accumulation of PARylation and ssDNA‐associated phosphorylation of RPA and CHK1 in EdU^+^ (replicating) cells, the latter process was further enhanced in combination treatment (Figure 6F–I), indicating a role of DHS in enhancing ssDNA gaps and PARylation associated with lagging strands processing. BRCA‐RAD51 is involved in the canonical OF processing while BRCA‐deficient cells heavily rely on PARP‐mediated backup pathway for OF processing.^[^ ^5^, ^55^ ^]^ Hence, downregulation of RAD51 along with enhanced fork pausing may possibly elicit PARylation‐mediated backup pathway for OF processing in response to DHS treatment. Mechanistically, no direct interaction of DHS was observed with PRIMPOL, which ruled out the generation of extensive ssDNA gaps through replication repriming (Figure 6A–C). Further, our extensive PLA study revealed a longer interaction time for PCNA replication machinery with nascent EdU‐tracts in DHS treated cells (Figure 7A–D). In combination treatment of DHS and PARPi, initial interactions of nascent EdU‐tracts with PCNA replication machinery were rapidly lost due to progressive fork collapse with time, as evident from the result showing enhanced PLA signals for nascent EdU‐tracts with 53BP1 (DSBs) (Figure 7D,J). Interestingly, although we observed enhanced PARylation in replicating cells (Figure 6D,E), interaction of nascent EdU‐tracts with PARylation was severely limited in DHS treatment (Figure 7F,G). This suggests a role of DHS in abrogating the interaction of stressed EdU tracts with PARylation behind the forks and OFP, leading to accumulation of ssDNA gaps, which were further enhanced in combination treatment due to insufficient PARylation (Figure 6D,E, Figure 7F,G, Figure 9P). Further, accumulation of ssDNA gaps resulted into MRE11 processing and cleavage of forks into DSBs (Figure 8E–K, Figure 9P). Many reports support the notion that DSBs arising from ssDNA gaps are repaired by RAD51 in HRP cells.^[^ ^14^, ^16^, ^56^ ^]^ DHS induced downregulation of RAD51 expression and HRR (Figures 1 and 3), which may further assist in accumulating collapsed/damaged replication forks (Figure 8J,K; Figures S42 and S43, Supporting Information), leading to accumulation of DNA damage and sensitization of ovarian cancers (Figures S42–S47, Supporting Information) and other types of cancers (Figures S48–S50, Supporting Information).

Finally, our in vivo results strongly support high efficacy of combination treatment of DHS and talazoparib in ovarian tumor xenograft model (Figure 9). The data for low toxicity of combination treatment, in normal mammary epithelial cells and animal experiments, further supports the tolerability of this combination treatment in clinical settings (Figure 9). Therefore, evaluation of combination of PARP inhibitor and DHS may be initiated in clinical settings for targeting homologous recombination proficient ovarian cancers.

## 4 Experimental Section

### Reagents and Plasmids

Talazoparib (BMN673) was purchased from ApexBio (Houston, TX, USA). 4,4′‐trans‐dihydroxystilbene (DHS) and other analogues were synthesized in‐house, as per the previous reports. Resveratrol (#R5010), 5‐Iodo‐2′‐Deoxyuridine (IdU), 5‐Chloro‐2′‐Deoxyuridine, Hoechst 33258, and antibodies for RAD51 (#PC130, RRID: AB_2238184), Phospho Histone H2AX (S139) (γ‐H2AX, #H5912, RRID: AB_477058 and #05‐636, RRID: AB_309864), 53BP1(#MAB3802, RRID: AB_2206767), β‐actin (#A5316, RRID: AB_476743) and all the commercially available stilbenes mentioned in the study were purchased from Sigma‐Aldrich (St. Louis, MO, USA). Antibodies for p‐Chk1 (CST #2348, RRID: AB_331212), PARP (CST #9542, RRID: AB_2160739), Rabbit IgG‐HRP conjugated (#7074, RRID: AB_2099233), Mouse IgG‐HRP conjugated (#7076, RRID: AB_330924), MRE11 (#4847, RRID: AB_10693469), Vinculin (#13901, RRID: AB_2728768) were from Cell signalling technology (MA, USA). Histone H3 (#ab1791, RRID: AB_302613) and CldU/BrdU antibody (#ab6326, RRID: AB_305426) was from Abcam, IdU/BrdU antibody (#347580, RRID: AB_10015219) was from BD Pharmingen (CA, USA) and pRPA32 (A300‐246A, RRID: AB_2180847) was from Bethyl Laboratories (TX, USA). PCNA (F‐2 clone, # SC‐25280, RRID: AB_628109), BRCA1 (#SC6954, RRID: AB_626761), BRCA2(#SC‐293185) antibodies were from Santa Cruz biotechnology (TX, USA). Click‐iT EdU Cell Proliferation Kit for Imaging‐Alexa Fluor 488 dye and 594 (# C10337 and C10339), PCNA (#13‐3900, RRID AB_2533016), RAD51 (#PA527195, RRID: AB_2544671) antibody was from Invitrogen, Thermo Fisher Scientific (Waltham, MA, USA). MRE11 antibody (#NB100‐473) was from Novus Bio (CO, USA), and RAD50 (#GTX70228) was from Genetex (USA). Secondary antibodies for Mouse IgG‐ Alexa flour 488 linked (#115‐546‐146, RRID: AB_2338868), Rabbit IgG‐ Alexa flour 594 linked (#115‐546‐144, RRID: AB_2338057), (Cy3 AffiniPure Donkey Anti‐Rat IgG (H+L), #712‐165‐153, RRID: AB_2340667) and Alexa Fluor 488 AffiniPure F(ab′)₂ fragment donkey anti‐mouse IgG (H+L), #715‐546‐151, RRID: AB_2340850) were from Jackson Laboratories (Bar Harbor, ME, USA). PRIMPOL antibody (# 29824‐1‐AP, RRID, AB_2918349) was procured from (ChromoTek GmbH, Germany). Wherever not specified, the other fine chemicals were procured from Sigma‐Aldrich (St. Louis, MO, USA). pDRGFP (#26475, RRID: Addgene_26475) homologous recombination reporter plasmid, pCBA‐ISceI (#26477, RRID: Addgene_26477) were procured from Addgene (Watertown, MA, USA).

### Cell Lines and Cell Culture

SK‐OV‐3 RRID: CVCL_0532, A2780 (RRID: CVCL_0134), OAW42 (RRID: CVCL_1615), MCF‐7 (RRID: CVCL_0031), U2‐OS (RRID: CVCL_0042), IMR32 (RRID: CVCL_0346), MIA‐PaCa‐2 (RRID: CVCL_0428) cell lines were procured from European Culture for Cell culture (ECACC). These cells were maintained in Dulbecco's Modified Eagle's medium with 10% foetal bovine serum with antibiotic and antimycotic solution (A002, Himedia laboratories, India) in an incubator (95% relative humidity; 5% CO_2_; 37 °C) for routine culturing. PA‐1 (RRID: CVCL_0479), MCF‐10A (RRID: CVCL_0598) cell lines were procured from American Type Culture Collection (ATCC, Virginia, USA). MCF‐10A cell line was cultured as per the instructions provided by ATCC, while PA‐1 cells were cultured similar to the cell lines above. Experiments were performed with cells not more than 6 passages old after thawing from the freeze vial stored in liquid nitrogen. Cells were certified, tested and authenticated by DNA profiling for polymorphic short tandem repeat markers by the supplier and were negative for mycoplasma throughout the current study.

### Clonogenic Assays

Clonogenic assay was performed as per previous report.^[^ ^32^ ^]^ Briefly, cells were seeded at a density of 500/1000 cells per well in a 12/6‐well plate. After overnight incubation, the cells were treated with vehicle (untreated control) or indicated concentrations of DHS, talazoparib or their combination, and were allowed to grow for a period of 7–10 days. Upon colonies reaching optimal counts in the well, they were washed with PBS and fixed with methanol, stained with 0.1% crystal violet for a duration of 15 mins. The stained colonies were counted by eye or with the help of a microscope. The data obtained was plotted to evaluate clonogenic survival.

### Cell Cycle and Apoptosis Analysis by Flow Cytometry

Cells were seeded at a density of 1.5 × 10^5^ per well in a 6‐well plate, left to attach overnight, treated, and flow cytometry analysis was carried out (Partec/Sysmex CyFlow® Space flow cytometer) after different durations as mentioned in the Figure legend to assess cell cycle and for sub‐G1 population analysis. Flow cytometry data analyses post acquisition was carried out using FlowJo software. Protocols were followed from the previous report.^[^ ^32^ ^]^

### Analysis of the Interaction of Drugs

Assessment of interaction of compounds was carried out using the Combenefit software (www.cruk.cam.ac.uk/research‐groups/jodrell‐group/combenefit).^[^ ^33^ ^]^ The mean of the data obtained in the individual clonogenic survival assays was tabulated in an.xls file as per the software requirements and subjected to Combenefit analysis. A graphical output of the drug‐interaction analysis was obtained and visualized as synergism (blue and cyan), additive effects (green) or antagonism (red).

### Immunoblotting/Western Blotting Analysis

Immunoblotting was carried out using our previously reported protocol.^[^ ^32^ ^]^ Briefly, cells were treated with talazoparib and DHS or DHS *plus* talazoparib for 24 h or as indicated in the Figure legends, followed by preparation of whole cell lysate, poly‐acrylamide gel electrophoresis, blotting transfer and the detection of the desired protein on the membrane. Densitometry analysis was performed for the protein bands on the membrane and normalized to that of the respective loading control. The intensity of the untreated sample was considered 1.

### Isolation of Chromatin Bound Protein Fraction and Immunoblotting

In a 90 mm dish, 1.5 × 10^5^ cells were seeded and treated post overnight incubation. Post 24 h of treatment, the cells were scraped off the culture dish and pelleted. Solution A (150 µL, 10 mM HEPES (pH‐ 7.4), 10 mM KCl, 1.5 mM MgCl_2_, 0.35 M Sucrose, 80% Glycerol, 1 M Dithiothreitol (DTT), 1% Triton X100, Protease inhibitor and phosphatase inhibitor cocktail) was added to the pellet and kept for 15 min with intermittent vortexing, the solution was then centrifuged at 1500 RPM for 5 min. The supernatant was taken out and stored as the cytoplasmic fraction. To the pellet, 150 µL of solution B (3 mM EDTA, 0.2 mM EGTA, 1 mM DTT, Protease inhibitor and phosphatase inhibitor cocktail) was added, kept for 20 min with intermittent vortexing, then centrifuged at 6000 RPM for 10 min. The supernatant was taken out and stored as the nuclear fraction. The pellet was washed with 300 µL solution C (1 mM DTT, Protease inhibitor and phosphatase inhibitor cocktail) twice by centrifugation at 10 000 RPM for 3 min. After the washes, the supernatant was taken out and 180 µL of solution D (Solution A components + 2 mM CaCl_2_, 2 mM MgCl_2_, DNase, and MNase) was added. Then the pellet was incubated for 30 min, with vortexing. 60 µL of 4X Laemmlli buffer was added to each of the tubes, then cooked at 95 °C for 10 min and used for immunoblotting.

### Immunofluorescence (IF) Microscopy

The protocol used was as described earlier with minor modifications.^[^ ^57^, ^58^ ^]^ Briefly, 1.5 × 10^5^ cells per well were seeded on glass cover slips in a 6‐well plate. Post overnight incubation, cells were treated as indicated in the Figure legends. After 24 h or indicated treatment duration, cells were fixed with 4% paraformaldehyde (PFA), permeabilized using phosphate buffered saline (PBS) containing 0.1% triton X‐100 for 10 min each and blocked with bovine serum albumin (BSA; 5% w/v in PBST, Tween 20). Antibodies for RAD51, 53BP1, γ‐H2AX, PCNA and p‐RPA32 were added at 1:6000 dilution and incubated overnight at 4 °C. An additional step of pre‐extraction was carried out prior to permeabilization in the case of RAD51 and pRPA32 foci formation assays (Pre‐extraction buffer‐50 mM HEPES, pH 7.4, 150 mM NaCl, 1 mM EDTA, 0.5% NP40, 100 µg mL^−1^ RNase). This was followed by 3X PBST washes and incubation with secondary antibody conjugated with Alexa Fluor 488/594 in BSA in PBST (2.5% w/v) for 3 h. Later, the samples were washed with PBST, and mounted onto glass slides with 80% glycerol containing Hoechst 33258 (25 µM). Image acquisition was carried out on a confocal microscope (LSM 780, Carl Zeiss, Oberkochen, Germany. All the parameters were kept constant between imaging of different samples of the same experiment. Image intensity and other analyses were performed using Zeiss Zen software.

### EdU Incorporation Assay

The protocol was followed from an earlier report with minor modifications.^[^ ^59^ ^]^ Cells were seeded as per the protocol in the previous section. During treatments, EdU (10 µM) was added to the cells for a time periods, as described in respective Figure legends. After treatments, the cells were fixed with 4% PFA and permeabilized. EdU staining was carried out according to the manufacturer's protocol. For experiments with IF analysis and EdU staining, the IF protocol was carried out first. This was followed by a mild fixation with 2% PFA for 10 min, washing and then the EdU staining.

### DNA Fiber Analysis

Analysis was performed as per previously published protocol with certain minor modifications.^[^ ^60^ ^]^ Briefly, SK‐OV‐3 cells were seeded at a density of 1.5 × 10^5^ cells per well in a 6‐well plate. After overnight incubation, the used medium was removed and pre‐warmed fresh DMEM medium was supplemented to the cells. CldU (20 µM), IdU (200 µM) and the appropriate treatments were supplemented in the medium as per the schemes provided in the respective Figures. After incubation duration, cells were trypsinized and a cell suspension of 1 × 10^6^ cells mL^−1^ was prepared for each sample. 2 µL of the cell suspension was carefully spotted on a clean grease free glass slide and was allowed to stand. To this, 10 µL of lysis buffer (50 mM EDTA, 0.5% SDS, 200 mM Tris pH 7.4) was added and the slides were incubated for 5 min at RT. The slide was then tilted at 10° to 25° angle and the drop was allowed to run along the length of the slide, air dried and then fixed using methanol: acetic acid solution (3:1). DNA fibers were then denatured using 2.5 M HCl for 1 h at RT and neutralized with 400 mM Tris‐HCl for 10 min. Slides were then blocked using 5% BSA for 1 h at RT. This was followed by the addition of primary antibodies for labeling the incorporated CldU and IdU for 3 h at RT. Following stringent washes, Alexa Fluor 594 and Alexa Fluor 488 conjugated secondary antibodies for detecting CldU and IdU primary antibodies respectively were added and incubated in dark condition for 1 h at RT. A drop of 80% glycerol was added and a coverslip was placed over the slide with DN fiber. Slides were then visualized and acquired under oil immersion at 40x magnification using LSM 780 laser scanning confocal microscope. DNA fibers were identified, lengths of the fibers were measured using the Zeiss Zen software. Fork speed was calculated by using conversion factor 2.59 kb µm^−1^ for stretched fiber as per previous report.^[^ ^61^ ^]^

### HR Reporter Assay

HR reporter plasmid (pDRGFP) was used to measure the HR activity in cells. In a 6‐well plate, 1.5 × 10^5^ per well stably expressing DRGFP reporter cells were seeded and after overnight incubation, transfected with pCBA‐ISceI using Lipofectamine 3000 (Thermo Fisher Scientific, MA, USA) as per the manufacturer's protocol. Post 24 h of transfection, cells were treated for 24 h and assayed for GFP**^+^** cells by flow cytometry. The protocol for this assay was followed from earlier reports.^[^ ^37^, ^58^ ^]^

### Proximity Ligation Assay (PLA)

PLA analysis was carried out between EdU and PCNA, EdU and PAR, and EdU and 53BP1 as per previous reports.^[^ ^39^, ^49^ ^]^ Briefly, SK‐OV‐3 cells were seeded on 15‐well micro‐angiogenesis slides (Ibidi GmbH, Germany) and incubated overnight. The cells were left untreated or treated with respective treatments and 50 µM EdU pulse was given for 10 min duration and washed off. Further, the medium with respective treatments was replenished during chase in the presence of 100 µM thymidine. Cells were further fixed with PFA after the chase duration and washed with PBS and incubated at room temperature with 10 µM biotin‐azide or Alexa Fluor‐488 tagged biotin‐azide for click reaction as per the manufacturer's protocol with some modifications (#C10337, Invitrogen). Alexa Fluor‐488 tagged biotin‐azide to biotin‐azide concentrations were used in 1:10 ratio. After the click reaction, cells were washed with PBS and then further procedures were as per the PLA kit manufacturer's protocol (Duolink® Proximity Ligation Assay kit, Sigma, St Louis, MO, USA).

### Neutral Comet Assay

Neutral comet assay was carried out as per previously reported protocol.^[^ ^62^ ^]^ The sample slides prepared were visualized on the Zeiss LSM780 confocal microscope and the images were analyzed using Cell Profiler software.

### Cellular Thermal Shift Assay (CETSA)

CETSA was performed as per the previous report with modifications.^[^ ^29^, ^46^ ^]^ Briefly, cells were seeded at 1.5 × 10^5^/well in a 6‐well plate and post overnight incubation, treated with DHS, or DMSO for 2 h. Cells were washed with ice‐cold PBS (with protease inhibitor cocktail), trypsinized, and aliquoted into PCR tubes. These tubes were incubated at different concentrations for minutes. Thereafter, the cell suspension was frozen and thawed thrice using liquid nitrogen followed by isolation of proteins from the cells by centrifugation at 20 000 g at 4 °C for 20 min and incubation at 95 °C for 5 min for analysis by Western blotting.

### Animal Studies

Animal studies and toxicity studies were performed as per the previously published report.^[^ ^15^ ^]^ Ethical clearance for experimentation was obtained from the Institutional Animal Ethics Committee, Bhabha Atomic Research Centre (IAEC, BARC; No. BAEC/10/16). SCID mice were purchased from Advanced Centre for Treatment, Research and Education in Cancer (ACTREC, Navi Mumbai, India) and housed in conventional individually ventilated polycarbonate shoebox cages at BARC animal house and fed and cared for according to regular IAEC recommendations in aseptic conditions. Exponentially growing SK‐OV‐3 cells (8 × 10^6^ per mouse) in 100 µL diluted DMEM diluted 1:1 in PBS were injected subcutaneously into the right flank of 6–8 weeks old healthy female SCID mice (weighing ≈20‐25 g, 5 mice per group). The mice were administered treatment in a laminar hood according to the indicated protocol after 30 days of tumor implantation following palpable tumor formation and randomization. Talazoparib (1 mg kg^−1^ in 100 µL; 0.5% DMSO in PBS), DHS (50 mg kg^−1^ in 100 µL HPCD) or their combination was administered by oral gavage on every alternate day for a total of 12 doses for 4 weeks. Evaluation of tumor volume (TV) on the indicated days was by measuring the perpendicular diameter axes of the tumor with calipers (Axes labelled ‘a’, long axis, and ‘b′, short axis). TV were calculated using the formula (V) = (a × b^2^)/2 and plotted. The animals were sacrificed at the end of the experiments by asphyxiation in a CO_2_ chamber. Weights of vital organs *viz*. heart, kidney and liver were measured post dissection of the sacrificed animal and plotted. Macroscopic examination of SCID mice was carried out after the sacrifice to evaluate treatment associated organ toxicity.

Toxicity analysis was also carried out in non‐tumor bearing Balb/c mice. Mice, aged between six and ten weeks, were randomly divided into two groups of 5 animals each. To determine the toxicity of the combination of DHS and talazoparib, a bolus dose equivalent to 5X (250 mg kg^−1^ DHS and 5 mg kg^−1^ talazoparib) that of the effective dose of each of the compound was administered. The body weight of these animals was monitored for any unusual signs due to acute toxicity *vis‐à‐vis* the control group.

Additionally, biochemical parameters of liver and kidney such as alkaline phosphatase (ALP), Alanine aminotransferase (ALT), glucose, cholesterol, total protein and creatine for the assessment of organ toxicity were studied. Post 24 h of administration of a bolus dose equivalent to 5X that of the effective dose of each of the compound in Balb/c mice, mice were anaesthetized and blood was drawn by cardiac puncture and serum collected for the assessment of biochemical parameters.

### Reagents, Synthesis, and Characterization of Stilbene Molecules

Commercially available stilbenes (8 nos) were procured from Sigma (St Louis, MO, USA). Synthesis and characterization of the other 12 stilbenes are mentioned in detail below. The resveratrol analogues (**ST6, ST10‐ST20**) were synthesized according to previously reported procedures with modifications.^[^ ^26^, ^31^, ^63^, ^64^ ^]^ Of note, synthesis of DHS (**ST6**) was achieved in one‐step via LVT‐mediated reductive coupling and in‐situ de‐benzoylation of 4‐formylphenyl benzoate (Figures S1 and S2, Supporting Information) in high yield (92%), which otherwise was not reported earlier. Synthesis of DHS analogue, (propargyl‐DHS; (E)‐4‐(4‐(prop‐2‐yn‐1‐yloxy)styryl)phenol), where one of its 4′‐OH is propargylated, is mentioned below. All the chemicals and solvents were commercially available (Aldrich) and used without further purification, unless specified. DCM was distilled over CaH_2_ whereas THF was dried over sodium. Infrared spectra were recorded on a Bruker Tensor II FTIR spectrophotometer and reported in wave numbers (cm^−1^). The ^1^H and ^13^C NMR spectra were recorded with 500 MHz Varian/200 MHz Bruker spectrometer in CDCl_3_/acetone‐*d6* and processed using MNova/Bruker TOPSPIN software. Chemical shifts are reported in ppm and calibrated relative to CDCl_3_ (δ_H_ = 7.26, δ_C_ = 77.00), acetone‐*d6* (δ_H_ = 2.05, δ_C_ = 29.92). Peak multiplicities are abbreviated as s, singlet; d, doublet; t, triplet; m, multiplet. The coupling constant *J* is reported in Hertz (Hz). Melting points were determined using Buchi M‐560 melting point apparatus and are uncorrected. Microanalyses were performed using a Vario Micro elemental analyzer.

*Synthesis of DHS (**ST6**)*: Earlier **ST6** was synthesized by Perkin condensation between 4‐hydroxybenzaldehyde and 4‐hydroxyphenylacetic acid but yield was very poor (25%).^[^ ^26^ ^]^ Also, another report^[^ ^31^ ^]^ mentioned McMurry coupling of unprotected 4‐hydroxybenzaldehyde for the synthesis of **ST6**. However, our attempt to synthesize **ST6** following this method gave poor yield with many unwarranted side products which were difficult to separate. Hence, we have modified our synthesis by using the benzoylated derivative of 4‐hydroxybenzaldehyde for McMurry coupling. This process gave our desired **ST6** in a single step in high yield (92%) without the need of further deprotection. Our synthetic scheme for 4,4′‐dihydroxystilbene (DHS) **ST6** was outlined in Figure 1B. The synthesis was started from 4‐hydroxybenzaldehyde **1**. To a solution of 4‐hydroxybenzaldehyde **1** (5.0 g, 40.94 mmol) in DCM (50 mL) was added triethylamine (11.4 mL, 81.88 mmol) and stirred for half an hour. Then benzoyl chloride (5.7 mL, 49.13 mmol) was added to it slowly at 0°C and stirred for 2.5 h at an ambient temperature. After completion (*cf*. by TLC) the reaction mixture was diluted with DCM (50 mL), washed with water (2 × 30 mL), brine (2 × 20 mL) and the combined organic extract was dried over anhydrous Na_2_SO_4_. The organic layer was concentrated in vacuum and the residue was purified by column chromatography (silica gel 100–200 mesh, 0–25% ethyl acetate in petroleum ether) to yield compound **2** (4‐formylphenyl benzoate) as a white crystalline solid (7.9 g, 85%). m.p.: 92.1‐92.3 °C; IR (neat) ʋ_max_, cm^−1^: 2722, 1736, 1693, 1590, 1258, 1203, 1054, 704. ^1^H NMR (500 MHz, CDCl_3_) δ 10.02 (s, 1H), 8.21 (d, *J* = 7.5 Hz, 2H), 7.98 (d, *J* = 8.0 Hz, 2H), 7.68‐7.65 (m, 1H), 7.54 (t, *J* = 7.5 Hz, 2H), 7.42 (d, *J* = 8.0 Hz, 2H); ^13^C NMR (125 MHz, CDCl_3_) δ 190.9, 164.5, 155.6, 134.0, 131.2, 130.2, 128.8, 128.7, 122.5. Anal. calculated for C_14_H_10_O_3_ (226.23): C, 74.33; H, 4.46; Found: C, 74.78; H, 4.49 (Figures S1 and S2, Supporting Information).

To a suspension of Zn (20.1 g, 309.4 mmol) in dry THF, TiCl_4_ (16.9 mL, 154.7 mmol) was added slowly at 0 °C under Argon and refluxed for 2 h. After that it was cooled to 0 °C, a solution of aldehyde **2** (5.0 g, 22.10 mmol) in dry THF was added to it slowly and refluxed for 3 h. After completion (*cf*. by TLC) the reaction was diluted with ethyl acetate, 10% K_2_CO_3_ solution was added to it slowly at 0 °C. Then the reaction mixture was filtered through a celite pad, washed with Ethyl acetate and the filtrate was extracted with brine. The organic layer was dried over anhydrous sodium sulphate. It was then concentrated under vacuum, the residue was washed with CHCl_3_ and dried to get pure DHS (**ST6)** as an off‐white solid (2.15 g, 92%). m.p.: 286.5‐286.6 °C; IR (neat) ʋ_max_, cm^−1^: 3352, 1599, 1509, 1446, 1370, 1247, 1103, 959, 829. ^1^H NMR (500 MHz, CDCl_3_) δ 8.55 (s, 2H), 7.39 (d, *J* = 8.5 Hz, 4H), 6.95 (s, 2H), 6.82 (d, *J* = 8.5 Hz, 4H); ^13^C NMR (125 MHz, CDCl_3_) δ 157.9, 130.5, 128.3, 126.5, 116.4. Anal. calculated for C_14_H_12_O_2_ (212.25): C, 79.23; H, 5.70; Found: C, 79.49; H, 5.49 (Figures S3 and S4, Supporting Information).

*Synthesis of (E)‐Tert‐Butyl(4‐(4‐Methoxystyryl)Phenoxy)Dimethylsilane (**ST15**) and (E)‐4‐(4‐Methoxystyryl)Phenol (**ST10**)*: To a stirred suspension of Zn powder (9.97 g, 152.5 mmol) in dry THF (150 mL) at 0 °C under argon, TiCl_4_ (8.4 mL, 76.3 mmol) was added slowly. The suspension was warmed to ambient temperature and then refluxed for 2.5 h. The black mixture was cooled in an ice‐water bath and a solution of 4‐methoxy benzaldehyde (3 g, 12.7 mmol) and 4‐((tert‐butyldimethylsilyl)oxy)benzaldehyde (1.73 g, 12.7 mmol) in THF (50 mL) was added and then the mixture was further refluxed until the starting aldehydes were consumed (monitored by TLC). The reaction mixture was cooled to an ambient temperature diluted with EtOAc and quenched with cold aqueous 10% K_2_CO_3_ solution. The mixture was stirred for 3 h and filtered through celite. The filtrate was washed with water, brine and dried (Na_2_SO_4_). Solvent was removed to get a residue which on purification by column chromatography (silica gel, 1% EtOAc in hexane) yielded the corresponding the corresponding cross‐coupled stilbene (**ST15**) along with two homo‐coupled symmetrical stilbenes.

*Synthesis of **ST10***: To a stirred solution of **ST15** (1.0 g, 2.94 mmol) in dry THF (10 mL) at 0 °C was added TBAF, 1 (M) in THF (3.23 mL, 3.23 mmol). The reaction mixture was then allowed to come at ambient temperature. After, 2 h the reaction mixture was quenched with ice‐water and extracted with ethylacetate. The organic layer was washed with brine and dried (Na_2_SO_4_). The organic solvent was removed and the crude material was purified by column chromatography to give pure **ST10**.


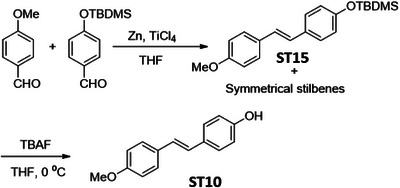


*(E)‐Tert‐Butyl(4‐(4‐Methoxystyryl)Phenoxy)Dimethylsilane (ST15)*: brown solid; yield: 32%; IR (solid): ʋ_max_ 2927, 2856, 1601, 1508, 1468, 1249, 1169, 1103, 1032, 969 cm^−1^; ^1^H NMR (200 MHz, CDCl_3_): δ 7.46‐7.36 (m, 4H), 6.94 (s, 2H), 6.92‐6.81 (m, 4H), 3.84 (s, 3H), 1.02 (s, 9H), 0.22 (s, 6H); ^13^C NMR (50 MHz, CDCl_3_): δ 159.0, 155.1, 131.0, 130.5, 127.4, 127.3, 126.3, 120.3, 114.1, 55.2, 25.7, 18.2, ‐4.4 (Figures S5 and S6, Supporting Information).

*(E)‐4‐(4‐Methoxystyryl)Phenol (**ST10**)*: brown solid; yield: 83%; IR (solid): ʋ_max_ 3411, 2921, 1603, 1510, 1439, 1364, 1301, 1236, 1171, 1103, 1015, 966, 954 cm^−1^; ^1^H NMR (200 MHz, acetone‐*d6*): δ 8.40 (br s, 1H, OH), 7.49‐7.45 (m, 2H), 7.42‐7.36 (m, 2H), 6.99 (s, 2H), 6.95‐88 (m, 2H), 6.82 (d, *J* = 8.6 Hz, 2H), 3.79 (s, 3H); ^13^C NMR (50 MHz, acetone‐*d6*): δ 159.7, 157.6, 131.2, 130.0, 128.1, 127.9, 126.9, 125.8, 116.1, 114.6, 55.2 (Figures S7 and S8, Supporting Information).

*Synthesis of (E)‐1,2‐bis(2′‐Methoxyphenyl)Ethene (**ST11**) and (E)‐1,2‐bis‐(2'‐Hydroxyphenyl)Ethene (**ST12**)*: Following the procedure adopted for synthesis of compound **ST15**, low‐valent titanium mediated reductive de‐oxygenation of 2‐methoxy benzaldehyde (1.5 g, 11.02 mmol) by using Zn powder (4.3 g, 66.1 mmol) and TiCl_4_ (3.63 mL, 33.06 mmol) afforded a crude product which on purification by column chromatography (10‐15% EtOAc/hexane) yielded the corresponding stilbene, **ST11**. For synthesis of **ST12**: To a stirred solution of **ST11** (480 mg, 2.0 mmol) in dry CH_2_Cl_2_ (15 mL) at −40 °C was added BBr_3_, 1.0 (M) in CH_2_Cl_2_ (4.2 mL, 4.2 mmol). The stirring was continued at −20 °C for 2 h and then for 16 h at an ambient temperature, when TLC showed complete consumption of the starting material. The reaction mixture was quenched with cold H_2_O (20 mL), the aqueous layer was extracted with ethylacetate (3 × 15 mL), and the combined organic extracts were washed with brine (2 × 10 mL). The organic layer was dried (Na_2_SO_4_), filtered, and concentrated in vacuo. The crude residue was purified by silica gel column chromatography (5% methanol in chloroform) to afford pure **ST12**.


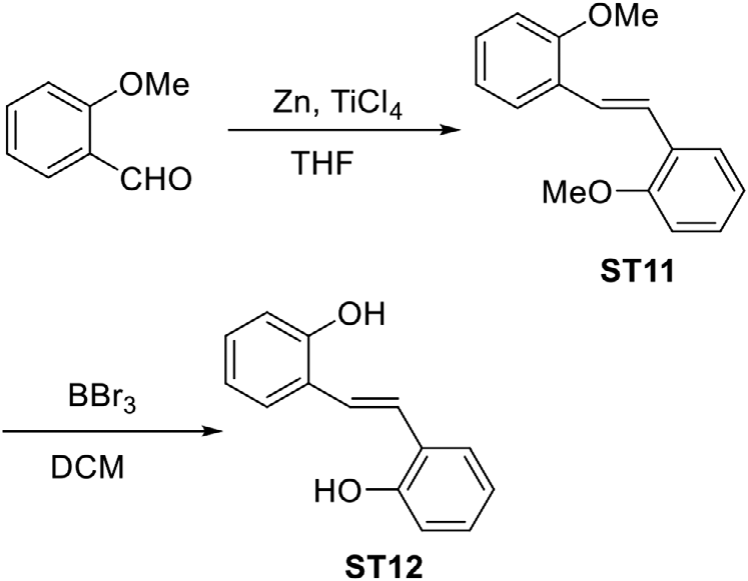


*(E)‐1,2‐bis(2‐Methoxyphenyl)Ethene (**ST11**)*: white solid; yield: 88%; IR (solid): ʋ_max_ 2999, 2922, 2852, 1595, 1578, 1492, 1331, 1246, 1160, 1027 cm^−1^; ^1^H NMR (200 MHz, acetone‐*d6*): δ 7.62 (dd, *J* = 7.6, 1.6 Hz, 2H), 7.48 (s, 2H), 7.28‐7.20 (m, 2H), 7.02‐6.91 (m, 4H), 3.88 (s, 6H); ^13^C NMR (125 MHz, acetone‐*d6*): δ 157.9, 129.5, 127.7, 127.0, 124.3, 121.6, 112.0, 56.0 (Figures S9 and S10, Supporting Information).

*(E)‐1,2‐bis‐(2′‐Hydroxyphenyl)Ethene (**ST12**)*: white solid; yield: 82%; IR (solid): ʋ_max_ 3286, 1596, 1555, 1485, 1383, 1218, 1106, 937, 835 cm^−1^; ^1^H NMR (200 MHz, acetone‐*d6*): δ 8.70 (br s, 2H), 7.58 (dd, *J* = 7.7, 1.6 Hz, 2H), 7.52 (s, 2H), 7.11‐7.03 (m, 2H), 6.90‐6.80 (m, 4H); ^13^C NMR (50 MHz, acetone‐*d6*): δ 154.6, 128.2, 126.2, 125.1, 123.2, 119.8, 115.7 (Figures S11 and S12, Supporting Information).

*Synthesis of (E)‐1,2‐bis(3′,4′‐Methylenedioxyphenyl)Ethene (**ST13**)*: Following the procedure adopted for synthesis of compound **ST15**, low‐valent titanium mediated reductive de‐oxygenation of 3,4‐methylenedioxybenzaldehyde (1.5 g, 10.0 mmol) by using Zn powder (3.9 g, 60.0 mmol) and TiCl_4_ (3.29 mL, 30.0 mmol) afforded a crude product which on purification by column chromatography (15‐20% EtOAc/hexane) yielded the corresponding stilbene, **ST13**.


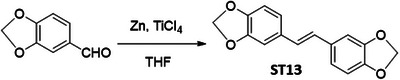


*(E)‐1,2‐bis(3′,4′‐Methylenedioxyphenyl)Ethene (**ST13**)*: brown solid; yield: 72%; IR (solid): ʋ_max_ 2995, 2840, 1585, 1458, 1345, 1273, 1056 cm^−1^; ^1^H NMR (500 MHz, acetone‐*d6*): δ 7.15 (d, *J* = 2.0 Hz, 2H), 7.03 (s, 2H), 7.00 (dd, *J* = 8.0, 2.0 Hz, 2H), 6.82 (d, *J* = 8.0 Hz, 2H), 6.00 (s, 4H); ^13^C NMR (125 MHz, acetone‐*d6*): δ 149.2, 148.1, 133.2, 127.6, 122.2, 109.1, 106.0, 102.1 (Figures S13 and S14, Supporting Information).

*Synthesis of (E)‐1,2‐bis(3′,5′‐Dimethoxyphenyl)Ethene (**ST14**)*: Following the procedure adopted for synthesis of compound **ST15**, low‐valent titanium mediated reductive de‐oxygenation of 3,5‐dimethoxy benzaldehyde (1.0 g, 6.0 mmol) by using Zn powder (2.35 g, 36.0 mmol) and TiCl_4_ (2.0 mL, 18.0 mmol) afforded a crude product which on purification by column chromatography (10‐15% EtOAc/hexane) yielded the corresponding stilbene, **ST14**.


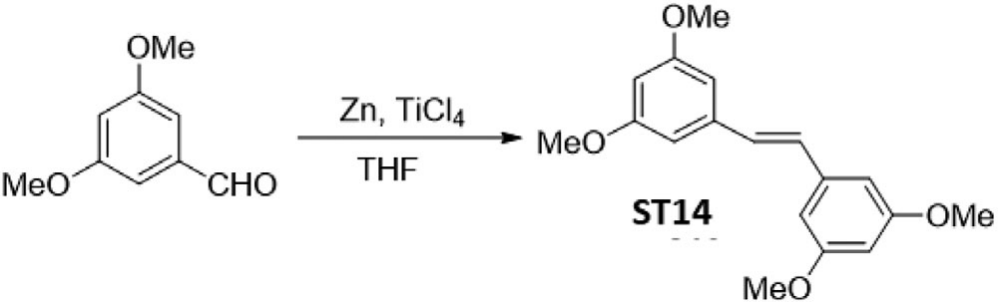


*(E)‐1,2‐bis(3′,5′‐Dimethoxyphenyl)Ethene (**ST14**)*: dark pink solid; yield: 86%; IR (solid): ʋ_max_ 2998, 2920, 2837, 1588, 1456, 1359, 1276, 1148, 1060 cm^−1^; ^1^H NMR (500 MHz, acetone‐*d6*): δ 7.19 (s, 2H), 6.77 (d, *J* = 2.5 Hz, 4H), 6.41 (t, *J* = 2.5 Hz, 2H), 3.81 (s, 12H); ^13^C NMR (125 MHz, acetone‐*d6*): δ 162.1, 140.3, 130.0, 105.4, 100.8, 55.7 (Figures S15 and S16, Supporting Information).

*Synthesis of (E)‐1‐(2′‐Hydroxyphenyl)‐2‐(3″,4″‐Methylenedioxyphenyl)Ethene (**ST16**)*: Following the procedure adopted for synthesis of compound **ST15**, low‐valent titanium mediated reductive de‐oxygenation of 2‐hydroxybenzaldehyde (1.22 g, 10.0 mmol) and 3,4‐methylenedioxybenzaldehyde (1.5 g, 10.0 mmol) by using Zn powder (7.8 g, 120.0 mmol) and TiCl_4_ (6.58 mL, 60.0 mmol) afforded a crude product which on purification by column chromatography (15‐20% EtOAc/hexane) yielded the corresponding stilbene, **ST16**.


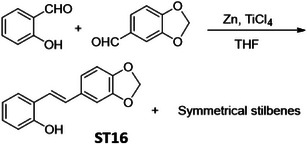


*(E)‐1‐(2′‐Hydroxyphenyl)‐2‐(3′,4''‐Methylenedioxyphenyl)Ethene (**ST16**)*: brown solid; yield: 60%; IR (solid): ʋ_max_ 3366, 3015, 2945, 2833, 1505 cm^−1^; ^1^H NMR (500 MHz, acetone‐*d6*): δ 8.85 (br s, 1H, OH), 7.55 (dd, *J* = 7.5, 1.5 Hz, 1H), 7.36 (d, *J* = 16.5 Hz, 1H), 7.16 (m, 2H), 7.07 (td, *J* = 7.5, 2.0 Hz, 1H), 7.01 (dd, *J* = 8.0, 1.5 Hz, 1H), 6.90 (dd, *J* = 8.0, 1.5 Hz, 1H), 6.85‐6.82 (m, 2H), 6.00 (s, 2H); ^13^C NMR (125 MHz, acetone‐*d6*): δ 155.7, 149.2, 148.1, 133.7, 129.1, 128.9, 127.3, 125.4, 123.0, 122.1, 120.6, 116.7, 109.1, 106.1, 102.1 (Figures S17 and S18, Supporting Information).

*Synthesis of (E)‐1,2‐bis‐(5′‐Chloro‐2′‐Hydroxyphenyl)Ethene (**ST17**)*: Following the procedure adopted for synthesis of compound **ST15**, low‐valent titanium mediated reductive de‐oxygenation of 5‐chloro‐2‐hydroxybenzaldehyde (1.56 g, 10.0 mmol) by using Zn powder (3.9 g, 60.0 mmol) and TiCl_4_ (3.29 mL, 30.0 mmol) afforded a crude product which on purification by column chromatography (15‐20% EtOAc/hexane) yielded the corresponding stilbene, **ST17**.


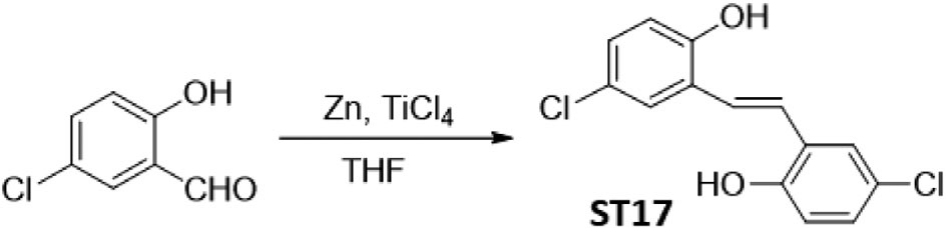


*(E)‐1,2‐bis‐(5′‐Chloro‐2′‐Hydroxyphenyl)Ethene (**ST17**)*: brown solid; yield: 76%; IR (solid): ʋ_max_ 3359, 3019, 1480, 1421 cm^−1^; ^1^H NMR (500 MHz, acetone‐*d6*): δ 9.07 (br s, 2H, OH), 7.59 (d, *J* = 2.5 Hz, 2H), 7.50 (s, 2H), 7.10 (dd, *J* = 8.5, 2.5 Hz, 2H), 6.93 (d, *J* = 8.5 Hz, 2H); ^13^C NMR (125 MHz, acetone‐*d6*): δ 153.6, 128.0, 126.5, 125.9, 124.2, 123.6, 117.2 (Figures S19 and S20, Supporting Information).

*Synthesis of (E)‐6‐(4‐Methoxystyryl)‐2,2‐Diphenyl‐2H‐Chromene (**ST18**)*: To a stirred solution of stilbene, **ST10** (226 mg, 1.0 mmol) and 1,1‐diphenylprop‐2‐yn‐1‐ol (312 mg, 1.5 mmol) in dry chloroform was added pyridinium‐para‐toluene sulfonate (50 mg). After 18 h of reflux, the reaction mixture was quenched with water and the aqueous layer was extracted with ethylacetate. The combined organic extracts were washed with brine, dried (Na_2_SO_4_), filtered, and concentrated in vacuo. The crude residue was purified by silica gel column chromatography (10‐15% EtOAc/hexane) to afford pure **ST18**.


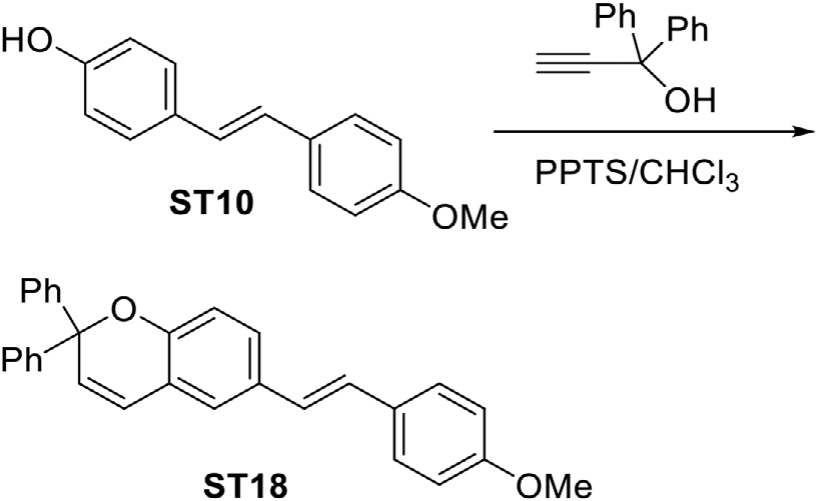


*(E)‐6‐(4‐Methoxystyryl)‐2,2‐Diphenyl‐2H‐Chromene (**ST18)***: white solid; yield: 24%; IR (solid): ʋ_max_ 3058, 2957, 2834, 1599, 1510, 1489, 1252, 1160, 1049, 989 cm^−1^; ^1^H NMR (500 MHz, CDCl_3_): δ 7.44‐7.42 (m, 4H), 7.40 (d, *J* = 8.5 Hz, 2H), 7.32 (t, *J* = 8.5 Hz, 4H), 7.27–7.24 (m, 3H), 7.15‐7.14 (m, 1H), 6.91‐6.86 (m, 5H), 6.64 (d, *J* = 10.0 Hz, 1H), 6.20 (d, *J* = 10.0 Hz, 1H) 3.82 (s, 3H); ^13^C NMR (125 MHz, CDCl_3_): δ 159.0, 152.0, 144.8, 131.0, 130.4, 129.2, 128.1, 127.6, 127.5, 127.4, 127.0, 126.3, 126.0, 124.2, 123.3, 121.1, 116.7, 114.1, 82.8, 55.3 (Figures S21 and S22, Supporting Information).

*Synthesis of (E)‐1,2‐bis‐(5′‐Bromo‐2′‐Hydroxyphenyl)Ethene (**ST19**)*: Following the procedure adopted for synthesis of compound **ST15**, low‐valent titanium mediated reductive de‐oxygenation of 5‐bromo‐2‐hydroxybenzaldehyde (2.0 g, 10.0 mmol) by using Zn powder (3.9 g, 60.0 mmol) and TiCl_4_ (3.29 mL, 30.0 mmol) afforded a crude product which on purification by column chromatography (15‐20% EtOAc/hexane) yielded the corresponding stilbene, **ST19**.


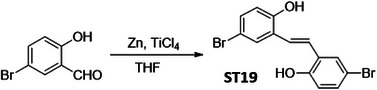


*(E)‐1,2‐bis‐(5′‐Bromo‐2′‐Hydroxyphenyl)Ethene (**ST19)***: brown solid; yield: 62%; IR (solid): ʋ_max_ 3333, 3019, 2400, 1478, 1215 cm^−1^; ^1^H NMR (500 MHz, acetone‐*d6*): δ 9.26 (s, 2H, OH), 7.71 (d, *J* = 2.5 Hz, 2H), 7.47 (s, 2H), 7.22 (dd, *J* = 8.5, 2.5 Hz, 2H), 6.87 (d, *J* = 8.5 Hz, 2H); ^13^C NMR (125 MHz, acetone‐*d6*): δ 155.0, 131.6, 129.6, 127.9, 124.2, 118.5, 112.1 (Figures S23 and S24, Supporting Information).

*Synthesis of (E)‐1,2‐bis‐(4′‐Methoxyphenyl)Ethene (**ST20**)*: Following the procedure adopted for synthesis of compound **ST15**, low‐valent titanium mediated reductive de‐oxygenation of 4‐methoxy benzaldehyde (3.0 g, 0.02 mol) by using Zn powder (8.62 g, 0.12 mol) and TiCl_4_ (7.24 mL, 0.06 mol) afforded a crude product which on crystallization from ethylacetate and hexane afforded pure **ST20**.


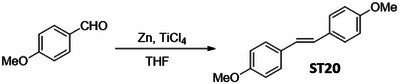


*(E)‐1,2‐bis‐(4‐Methoxyphenyl)Ethene) (ST20)*: white crystalline solid; yield: 84%; IR (solid): ʋ_max_ 3015, 2954, 2838, 1602, 1574, 1509, 1464, 1305, 1244, 1175, 1026, 967 cm^−1^; ^1^H NMR (200 MHz, acetone‐*d6*): δ 7.49 (d, *J* = 8.9 Hz, 4H), 7.03 (s, 2H), 6.92 (d, *J* = 8.9 Hz, 4H), 3.80 (s, 6H) (Figure S25, Supporting Information).

*Synthesis of Propargyl‐DHS [(E)‐4‐(4‐(Prop‐2‐yn‐1‐Yloxy)Styryl)Phenol]*: To a solution of *trans*‐4,4′‐dihydroxystilbene (0.20 g, 0.94 mmol) in dry acetonitrile was added K_2_CO_3_ (0.17 g, 1.22 mmol) and propargyl bromide, 80 wt. % in toluene (0.12 mL, 1.03 mmol). The resulting solution was refluxed until the TLC showed complete disappearance of the starting material. After 24 h the reaction mixture was poured in ice‐cold dilute HCl and extracted with ethylacetate. The organic layer was then washed with brine, dried (anhydrous Na_2_SO_4_) and concentrated in vacuo. The crude solid thus obtained was purified by silica‐gel column chromatography (3% MeOH in CHCl_3_) to afford the desired product.


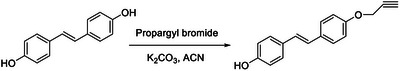


*(E)‐4‐(4‐(Prop‐2‐yn‐1‐Yloxy)Styryl)Phenol*: white solid; 23% yield; IR (solid): *ν* 3287, 2131, 1605, 1512, 1453, 1375, 1304, 1243, 1174, 1105, 1021, 960, 931, 832 cm^−1^; ^1^H NMR (200 MHz, CD_3_COCD_3_): δ 8.52 (bs, 1H), 7.49 (d, *J* = 9.0 Hz, 2H), 7.40 (d, *J* = 8.6 Hz, 2H), 7.00‐6.96 (m, 4H), 6.82 (d, *J* = 8.6 Hz, 2H), 4.79 (d, *J* = 2.4 Hz, 2H), 3.08 (t, *J* = 2.4 Hz, 1H); ^13^C NMR (50 MHz, CD_3_COCD_3_): δ 158.0, 157.9, 132.4, 130.2, 128.5, 128.2, 127.7, 126.0, 116.4, 116.0, 79.8, 77.0, 56.9. (Figures S32 and S33, Supporting Information)

### Statistical Analyses

Parametric and non‐parametric tests performed to evaluate the statistical significance of the data presented is described in the respective Figure legends. For experiments aside from DNA fiber experiments, values are presented as Mean ± SD or SEM and are mentioned in the legends wherever they apply. Two‐tailed ANOVA with Tukey post‐hoc analysis was performed to assess the significance of the data presented. In DNA fiber experiment plots, the bar represents the Median value. Kruskal‐Wallis test with Dunn's post‐hoc analysis was performed in the DNA fiber analysis to assess the significance of the data presented. In most of the experiments, a minimum of three independent biological replicates were performed. The exact number of biological replicates (N) and/or (n) (n: number of entities quantified) are mentioned in the respective Figure legends. A value of *p* < 0.05 was considered significant in all the cases throughout the paper. GraphPad Prism 9.0 software was used for all the statistical analyses and generation of plots.

## Conflict of Interest

The authors declare no conflict of interest.

## Author Contributions

Conceptualization: G.P.B. and B.S.P.; data curation: G.P.B., K.K., and B.S.P.; formal analysis: G.P.B., K.K., and B.S.P.; funding acquisition: B.S.P.; investigation: G.P.B., K.K., P.G., N.C., M.S., S.R., and B.S.P.; methodology: G.P.B., K.K., P.G., B.S.P.; project administration: B.S.P.; resources: G.P.B., K.K., P.G., B.S.P.; supervision: B.S.P.; validation: G.P.B., K.K., P.G., and B.S.P.; visualization: G.P.B., K.K., and B.S.P.; writing – original draft: G.P.B., K.K., and B.S.P.; writing – review & editing: G.P.B. and B.S.P. All authors have read and approved the final manuscript.

## Supporting information



Supporting Information

## Data Availability

The data generated in this study are available within the article and its supplementary data files. All raw data generated in the study are available upon request.
