# Supplementary material for: Targeting Replication Fork Processing Synergizes with PARP Inhibition to Potentiate Lethality in Homologous Recombination Proficient Ovarian Cancers
Source: Adv Sci (Weinh). 2025 Mar 16;12(18):2410718. doi: 10.1002/advs.202410718 (PMC12079468; doi:10.1002/advs.202410718)
Supplement: Supplementary file 1 — Supporting Information [file ADVS-12-2410718-s001.docx]

**Supporting Information**

**Targeting replication fork processing synergizes with PARP inhibition to potentiate lethality in homologous recombination proficient ovarian cancers**

Ganesh Pai Bellare ^1,2^, Kshama Kundu ^1^, Papiya Dey ^1^, Krupa Thankam Philip^1,2^, Nitish Chauhan ^1,2^, Muskan Sharma ^1^, Sankarsingh Kesharsingh Rajput ^1^, Birija Sankar Patro ^1,2*^

^1^ Bio-Organic Division, Bhabha Atomic Research Centre, Mumbai-400085, India.

^2^ Homi Bhabha National Institute, Anushaktinagar, Mumbai-400094, India.


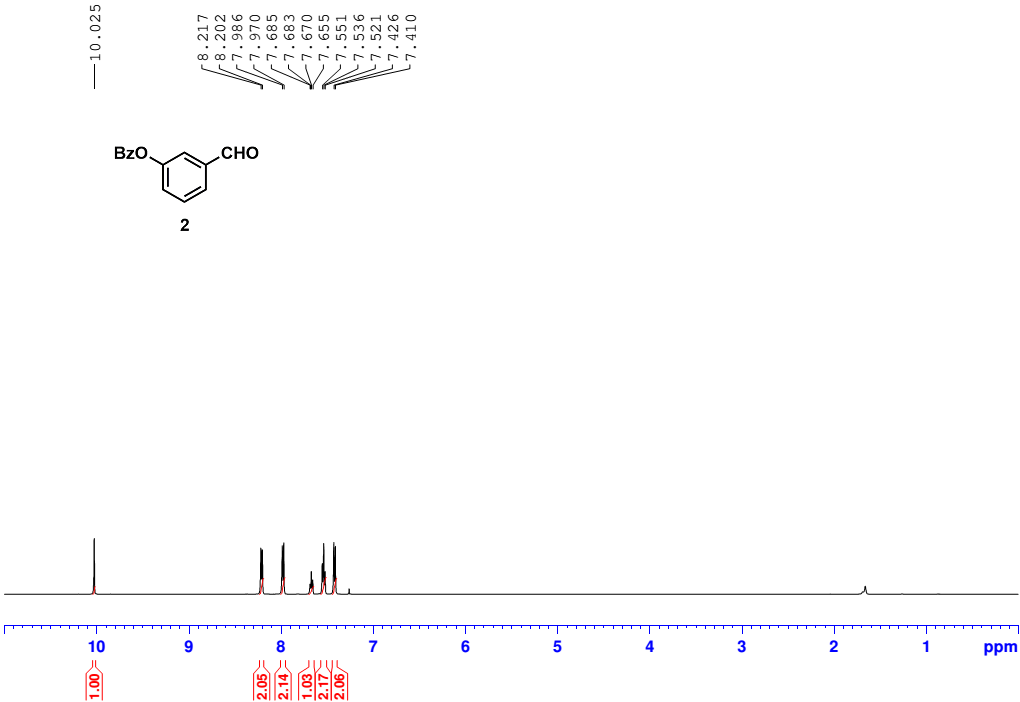


**Figure S1: ^1^H NMR spectrum of compound 2 (4-formylphenyl benzoate)**


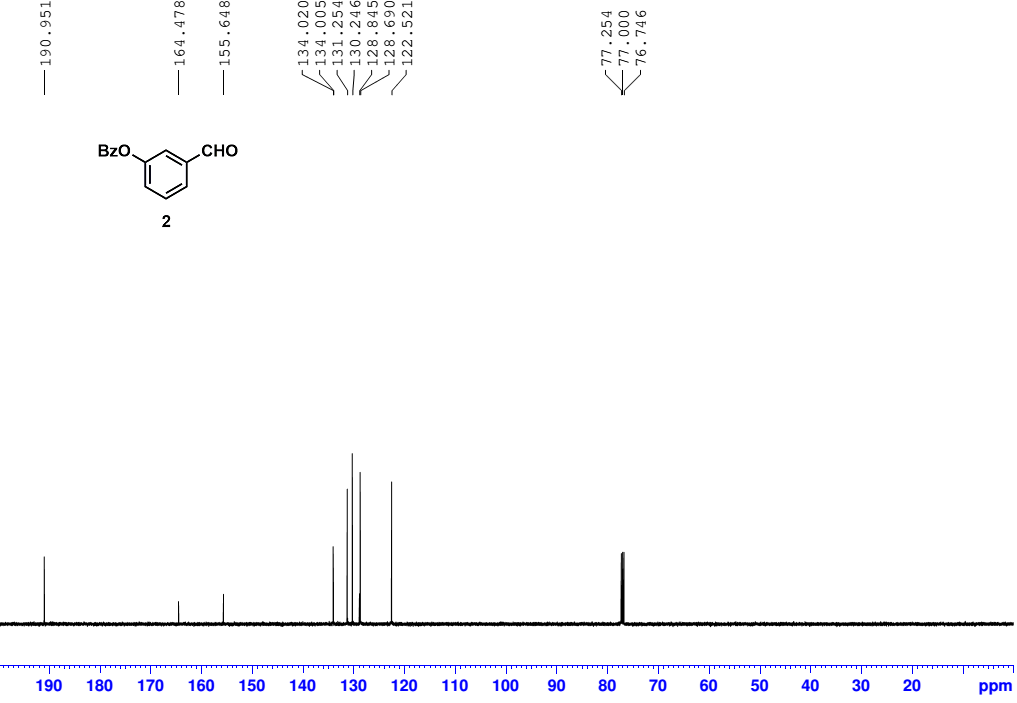


**Figure S2: ^13^C NMR spectrum of compound 2 (4-formylphenyl benzoate)**


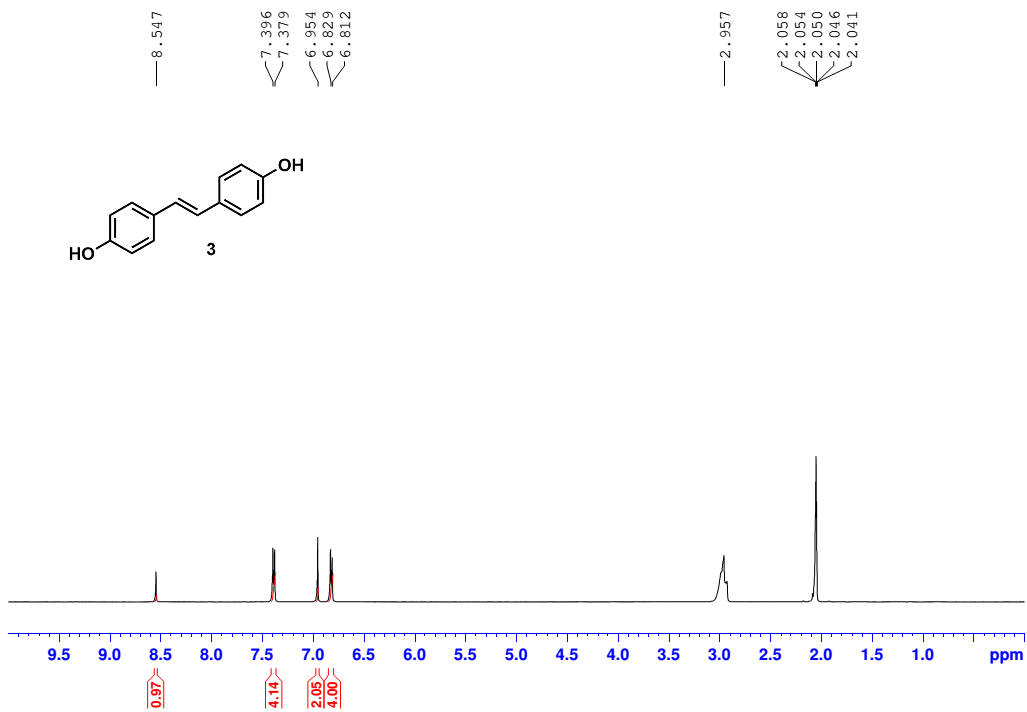


**Figure S3: ^1^H NMR spectrum of compound 3 (ST6, (E)-4,4'-(ethene-1,2-diyl)diphenol)**


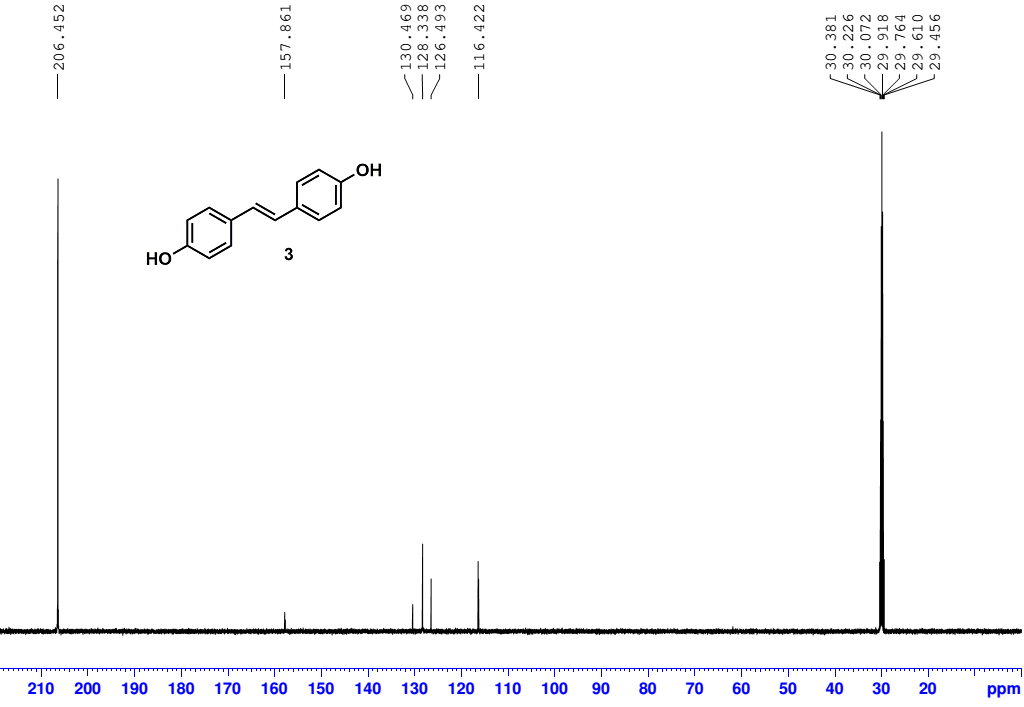


**Figure S4: ^13^C NMR spectrum of compound 3 (ST6, (E)-4,4'-(ethene-1,2-diyl)diphenol)**

**_
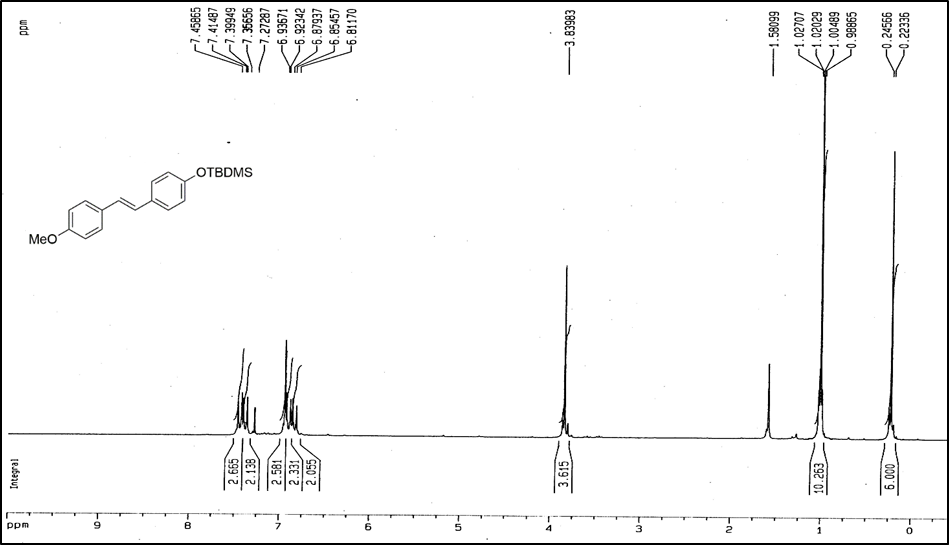
_**

**Figure S5: ^1^H NMR spectrum of ST15**

**
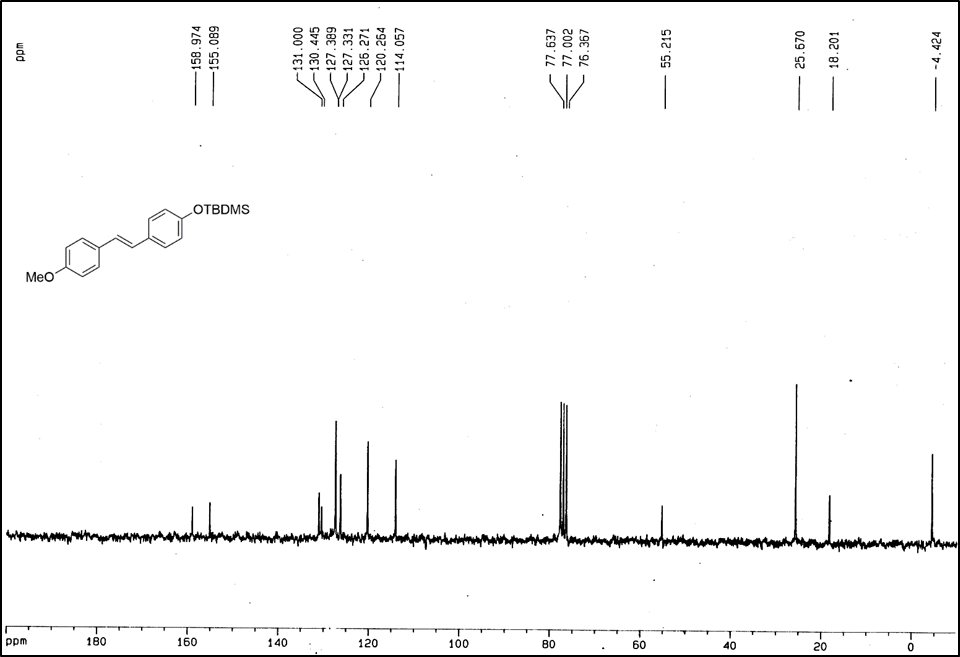
**

**Figure S6: ^13^C NMR spectrum of ST15**

**
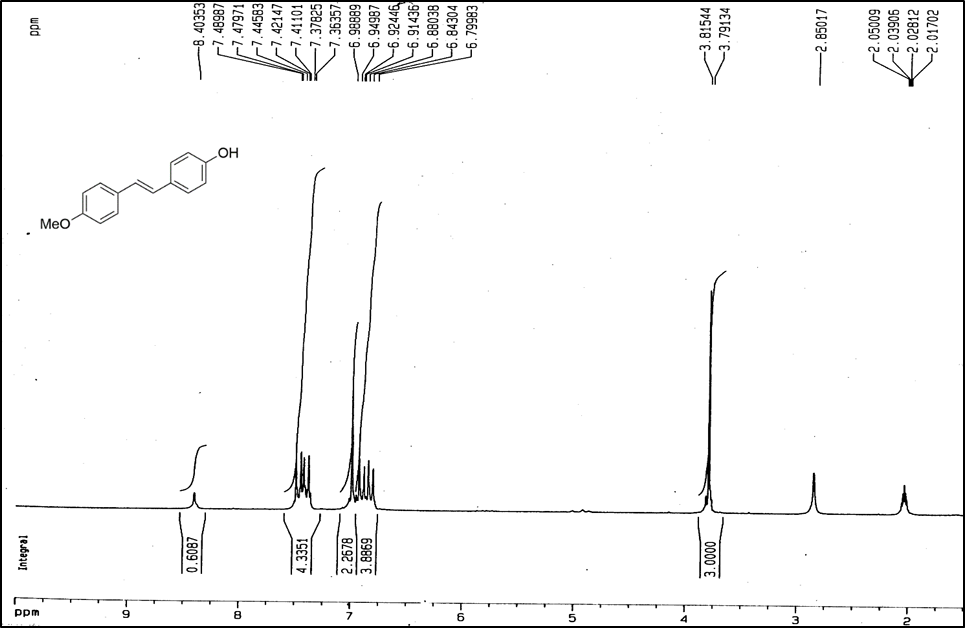
**

**Figure S7: ^1^H NMR spectrum of ST10**

**_
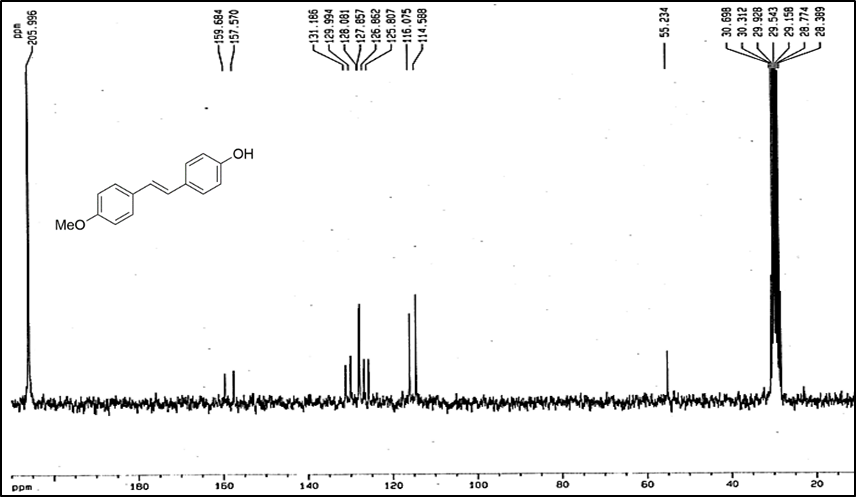
_**

**Figure S8: ^13^C NMR spectrum of ST10**


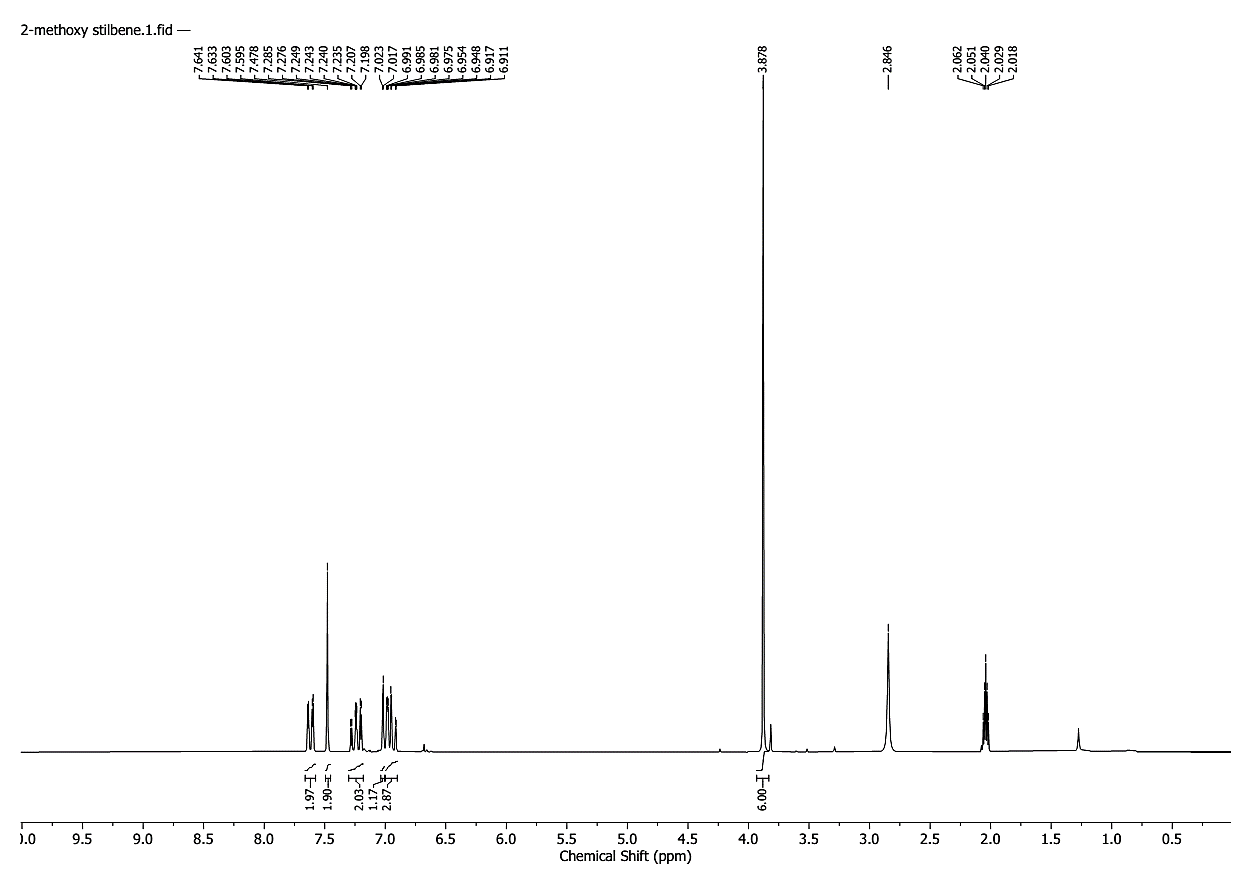


**Figure S9: ^1^H NMR spectrum of ST11**


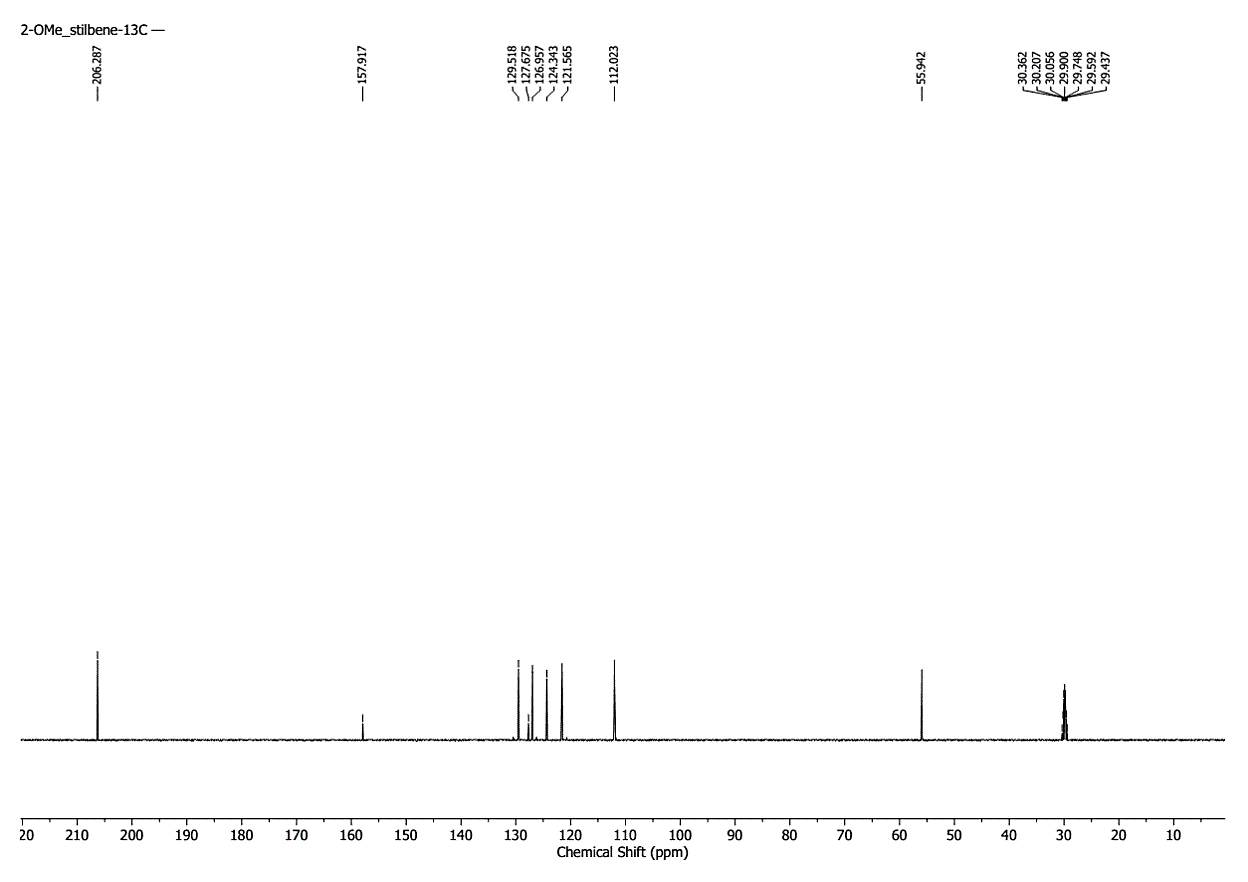


**Figure S10: ^13^C NMR spectrum of ST11**




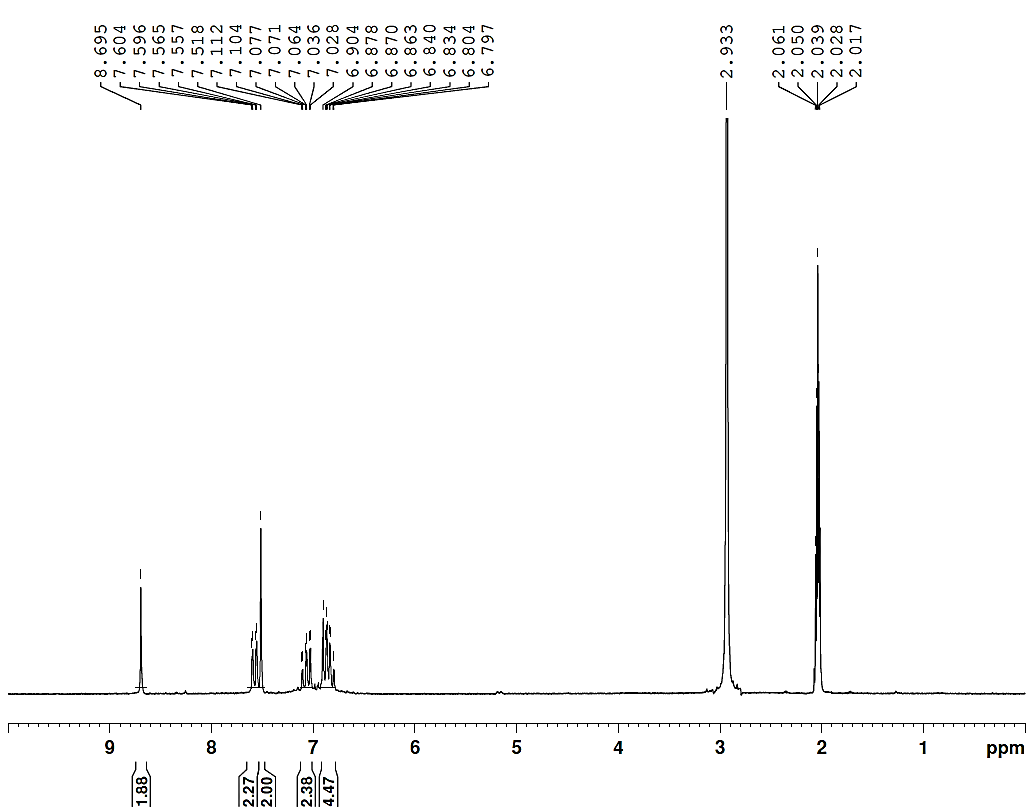


**Figure S11: ^1^H NMR spectrum of ST12**

**_
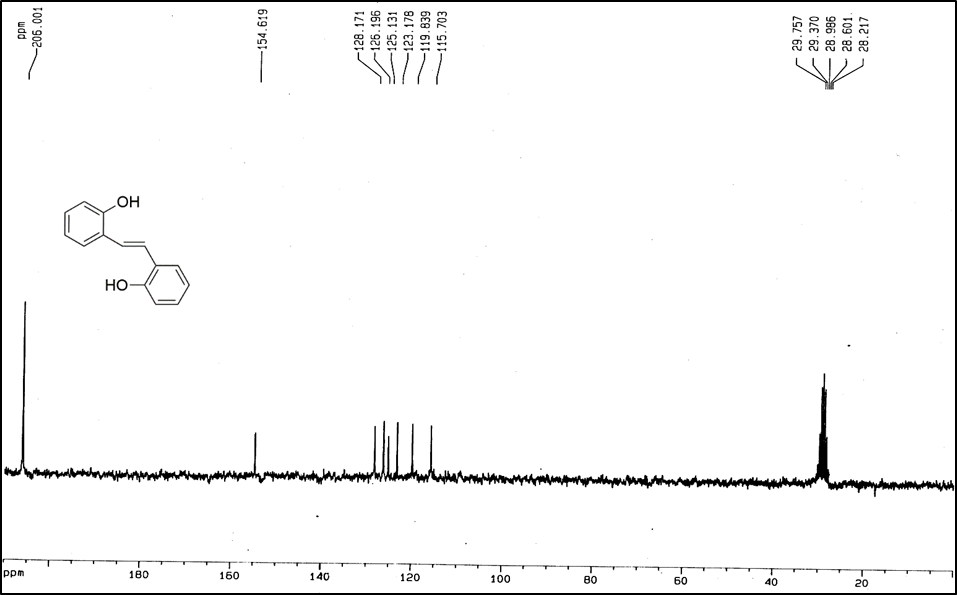
_**

**Figure S12: ^13^C NMR spectrum of ST12**


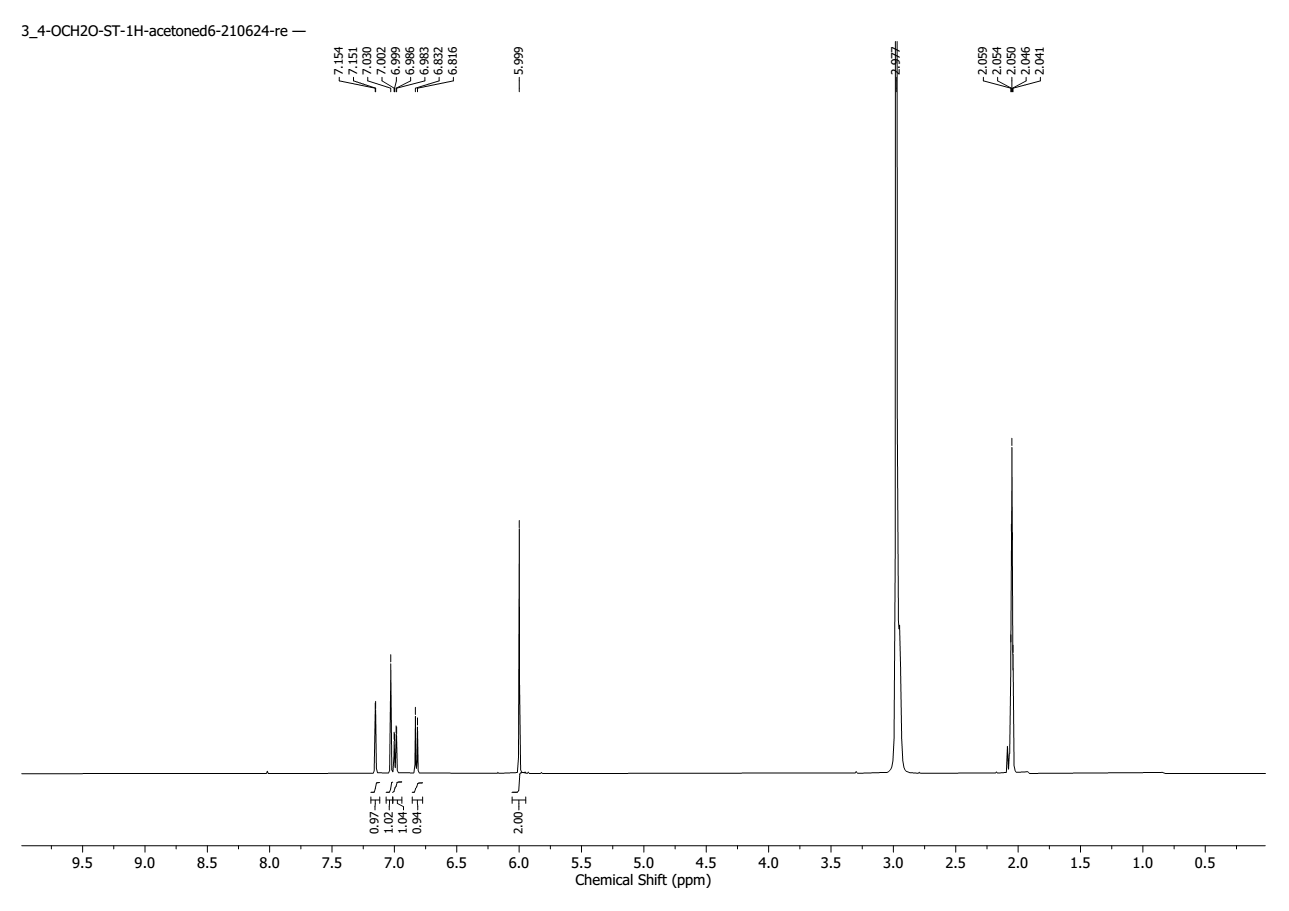


**Figure S13: ^1^H NMR spectrum of ST13**


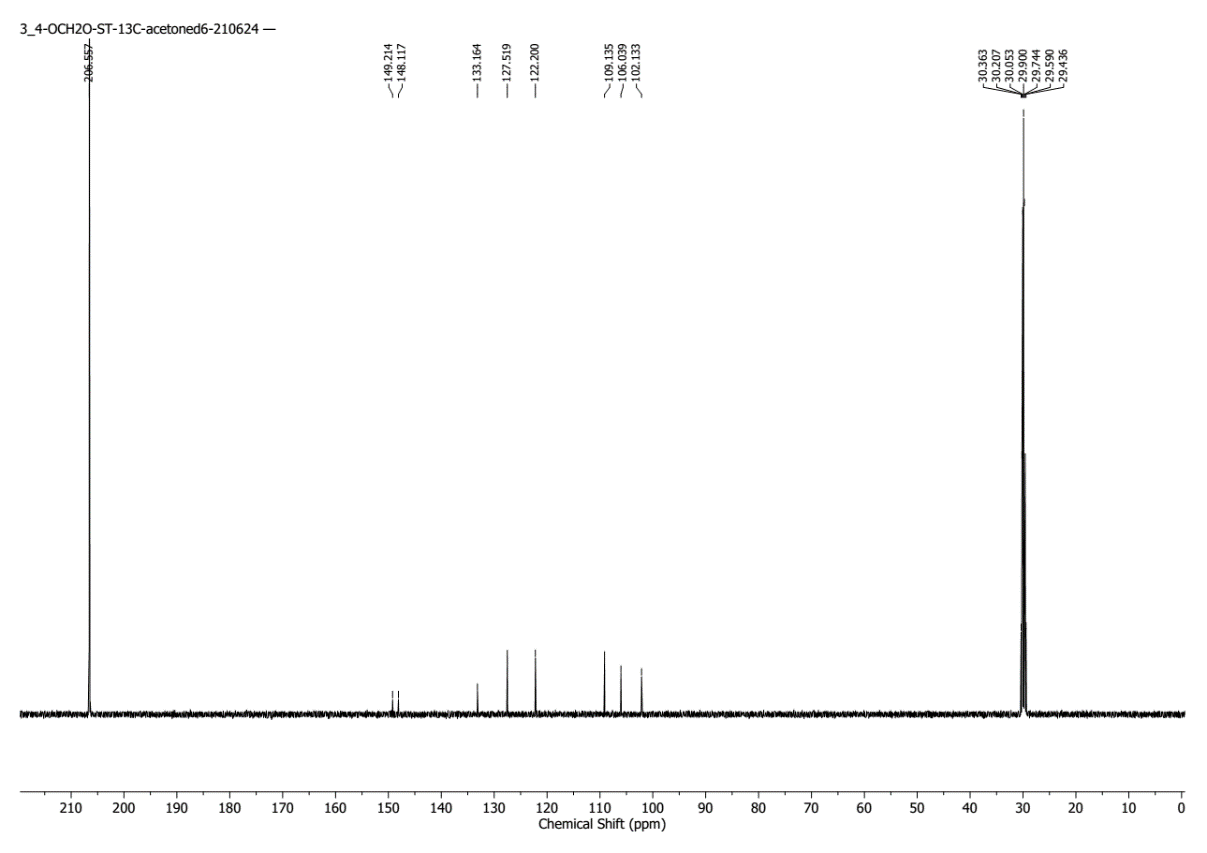


**Figure S14: ^13^C NMR spectrum of ST13**


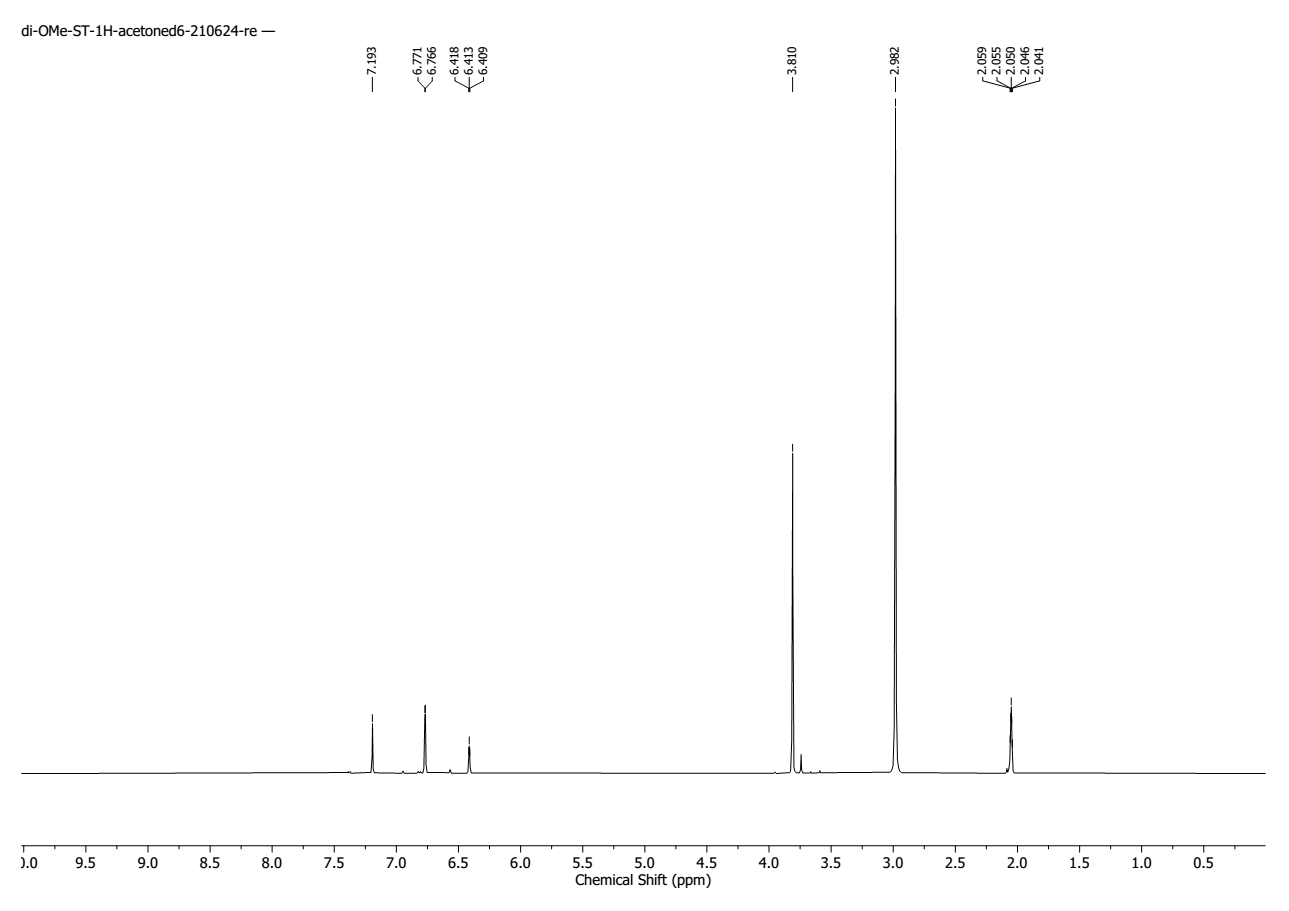


**Figure S15: ^1^H NMR spectrum of ST14**


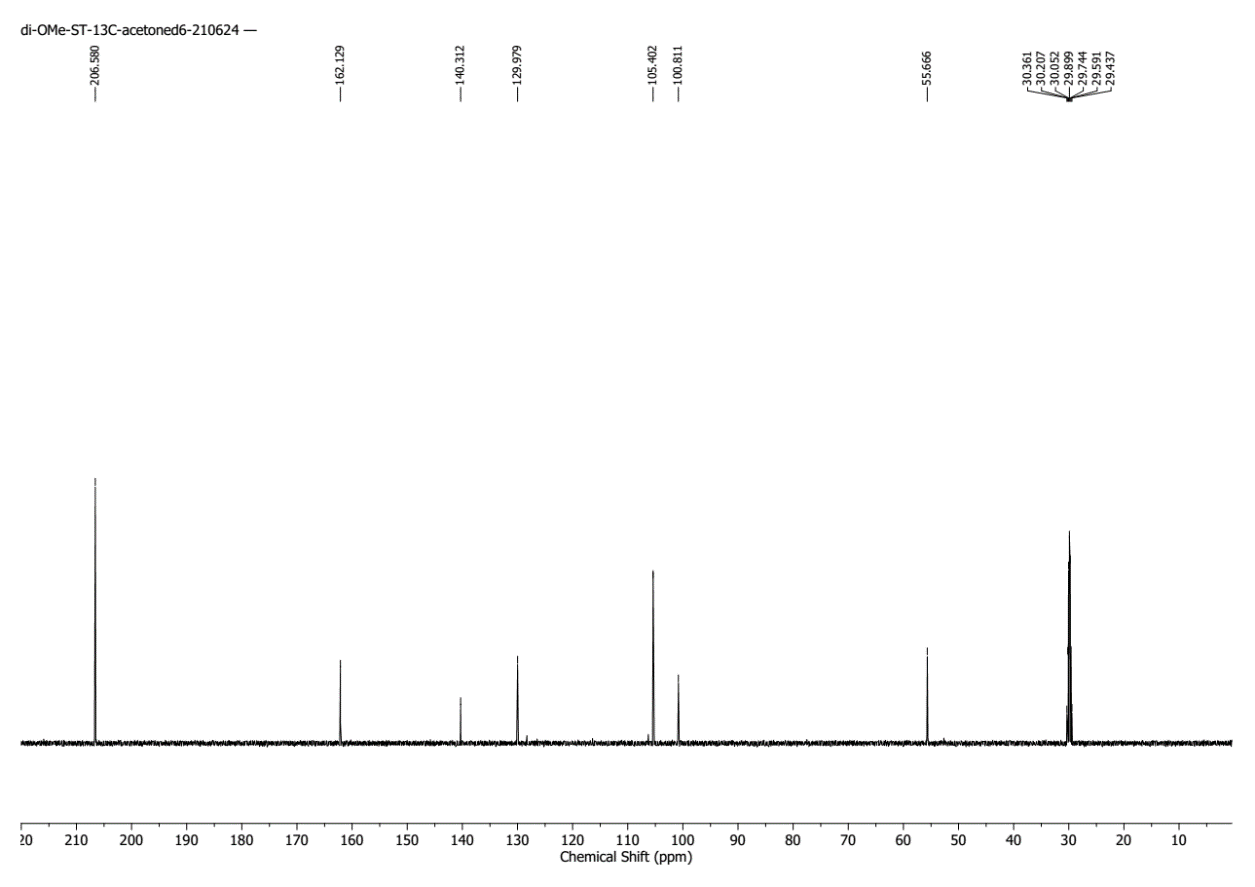


**Figure S16: ^13^C NMR spectrum of ST14**


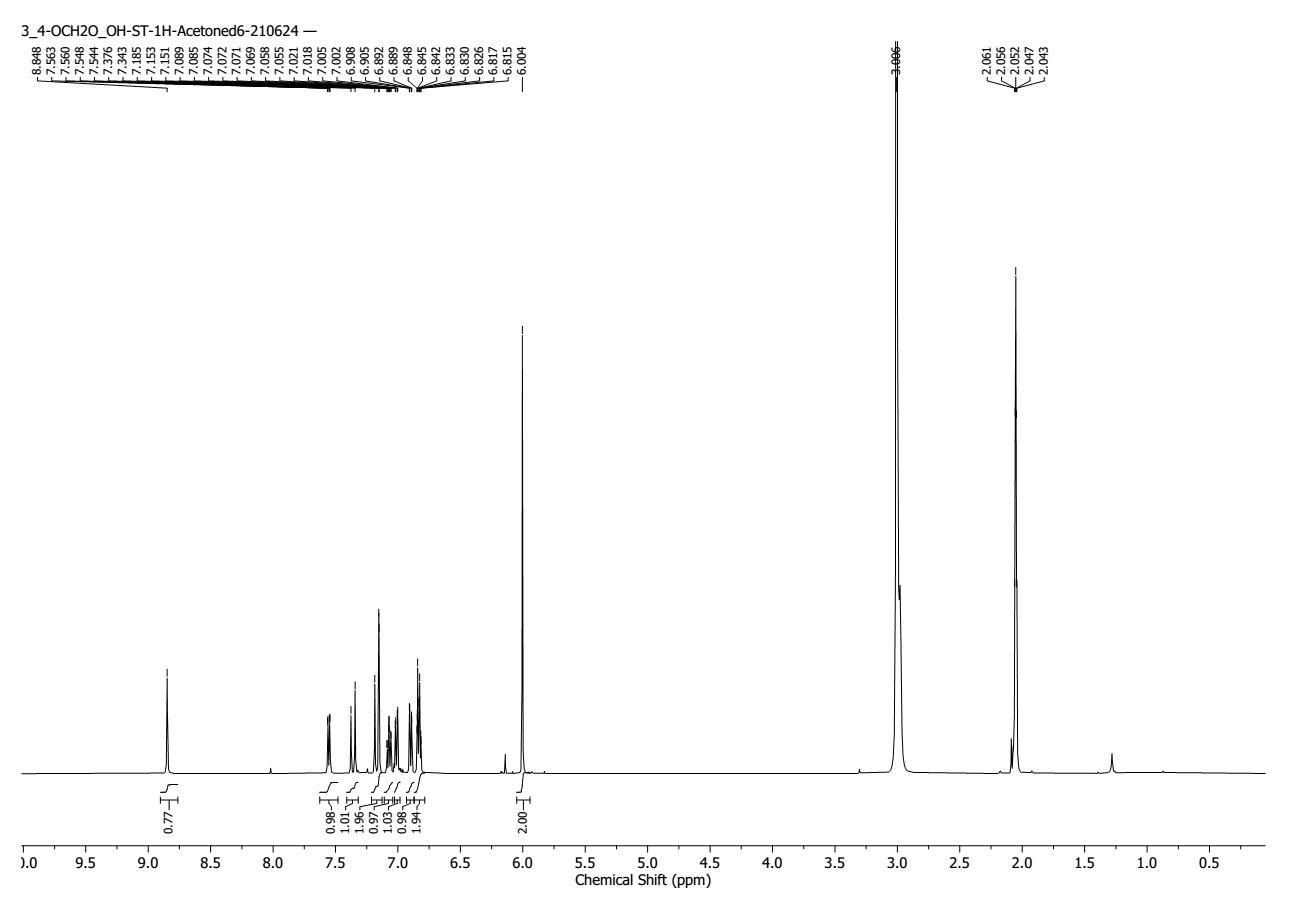


**Figure S17: ^1^H NMR spectrum of ST16**


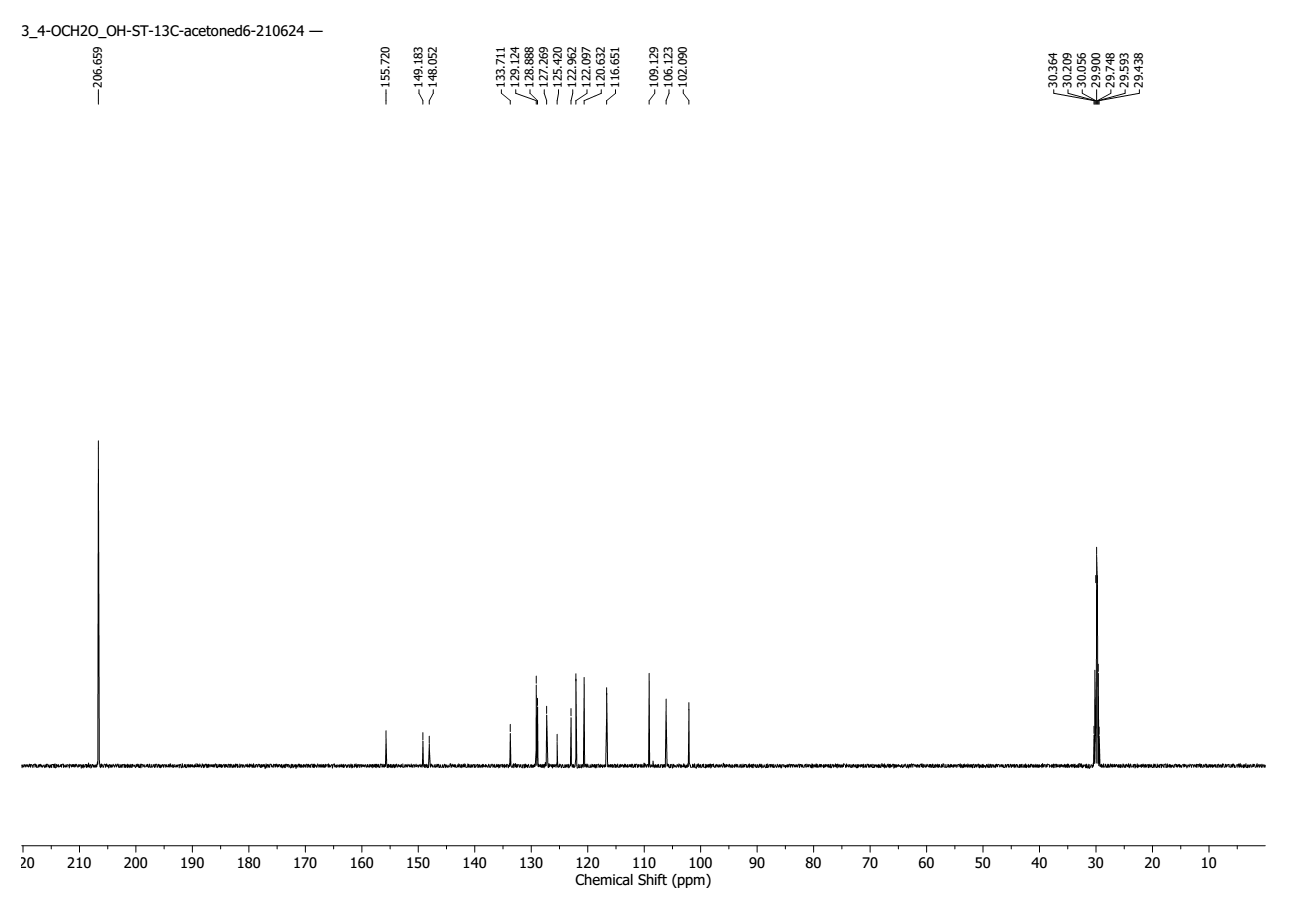


**Figure S18: ^13^C NMR spectrum of ST16**


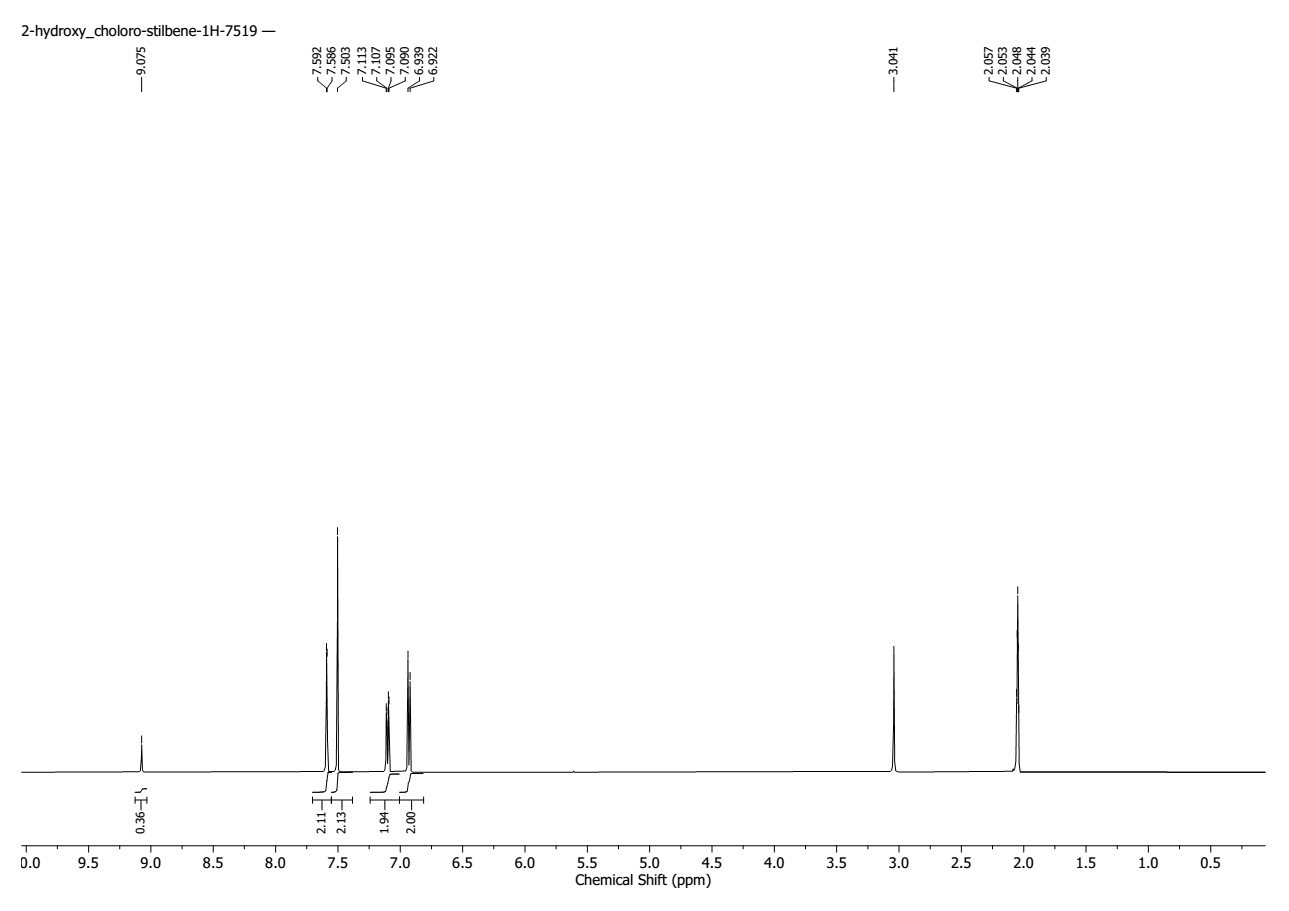


**Figure S19: ^1^H NMR spectrum of ST17**


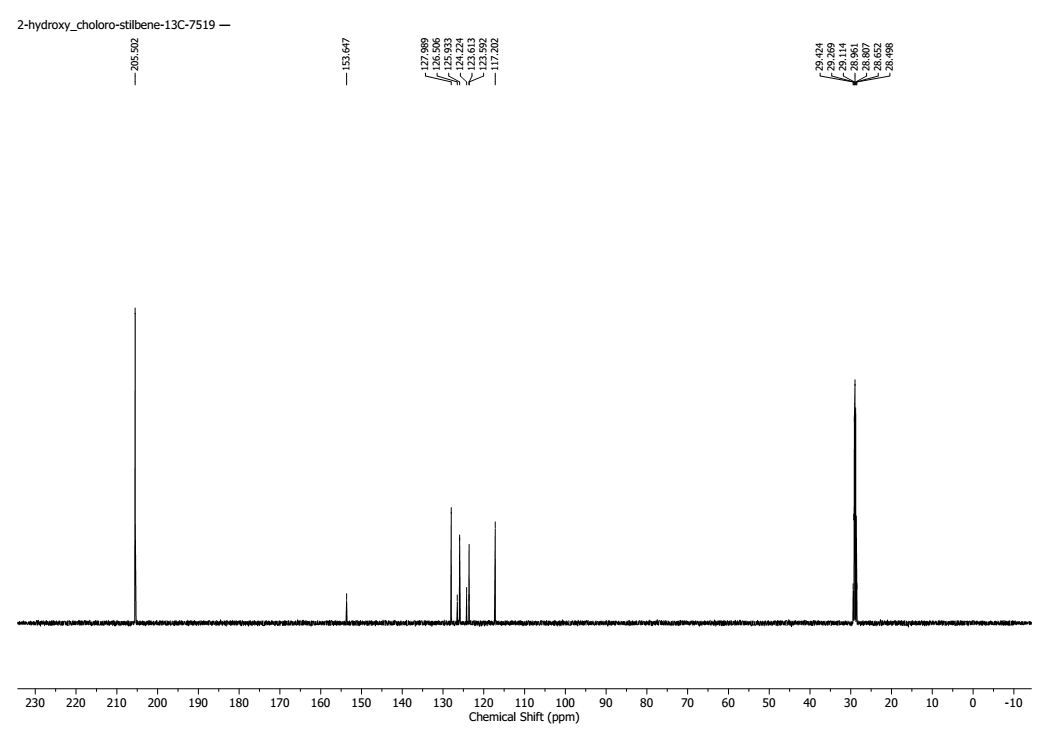


**Figure S20: ^1^H NMR spectrum of ST17**


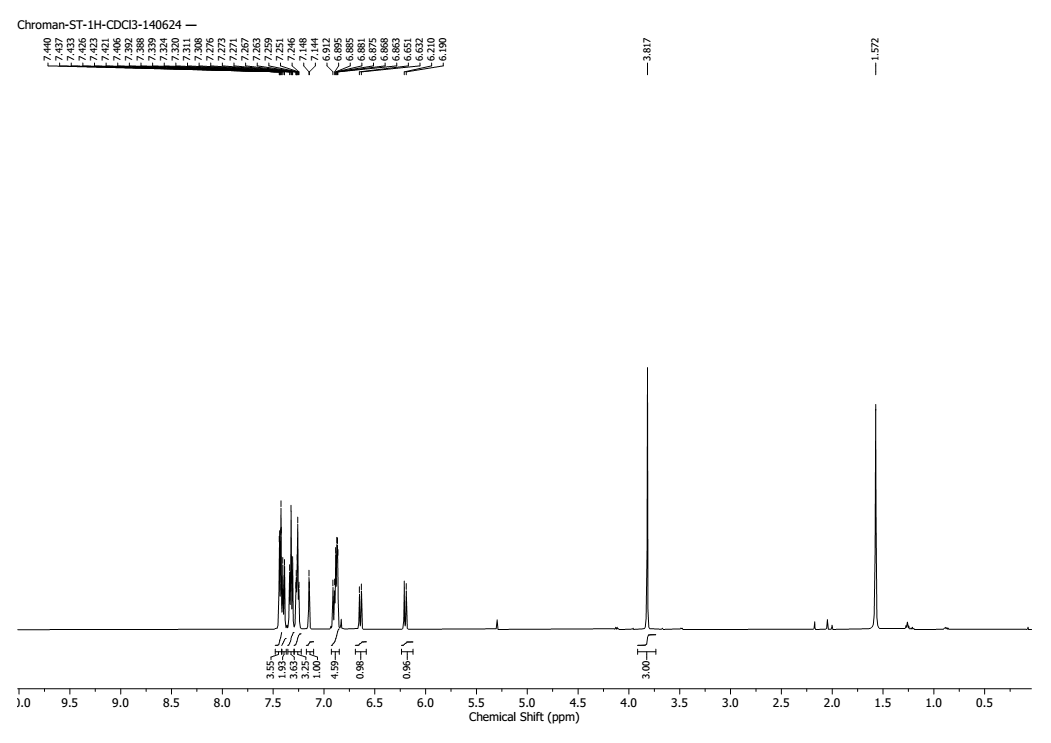


**Figure S21: ^1^H NMR spectrum of ST18**


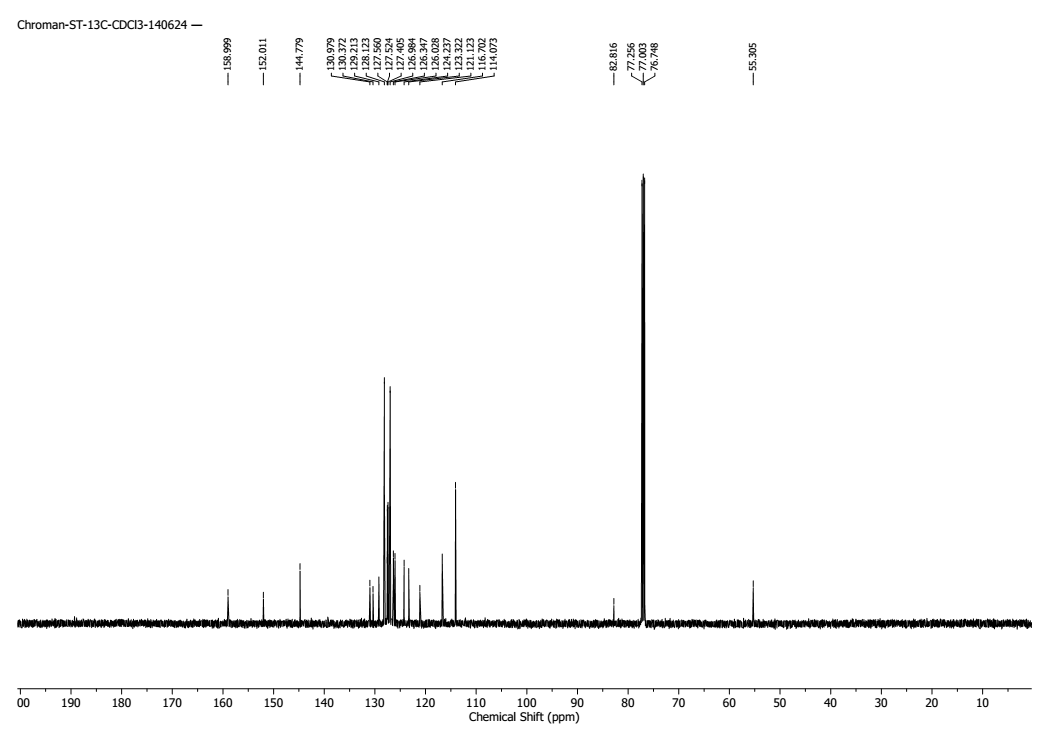


**Figure S22: ^13^C NMR spectrum of ST18**


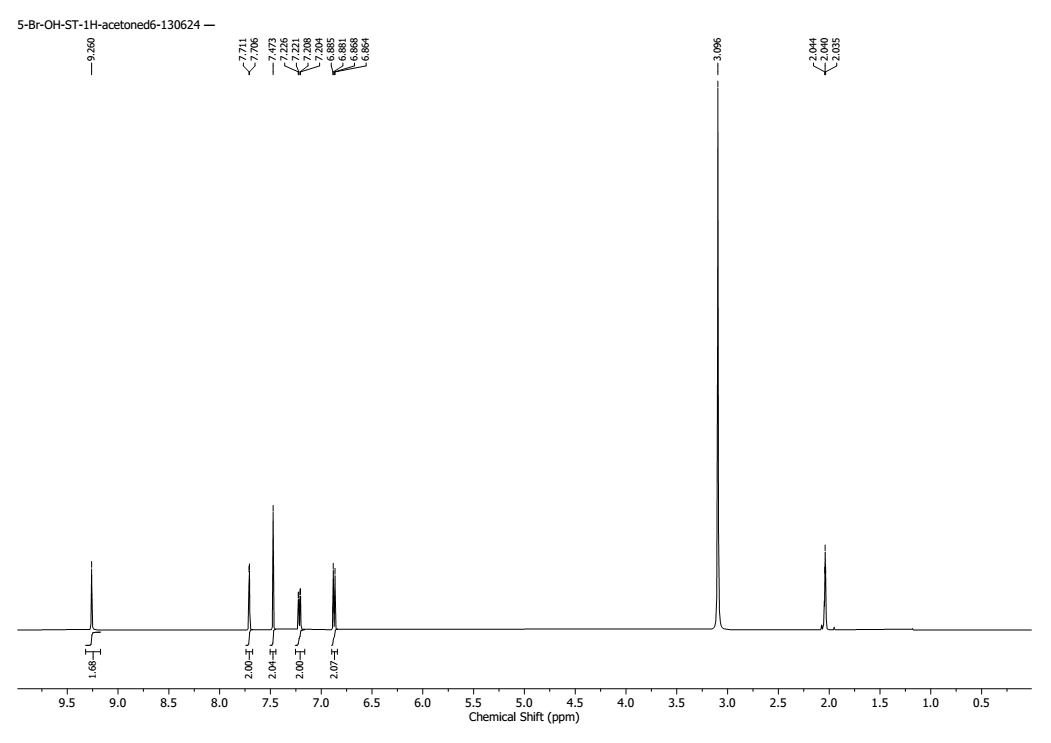


**Figure S23: ^1^H NMR spectrum of ST19**


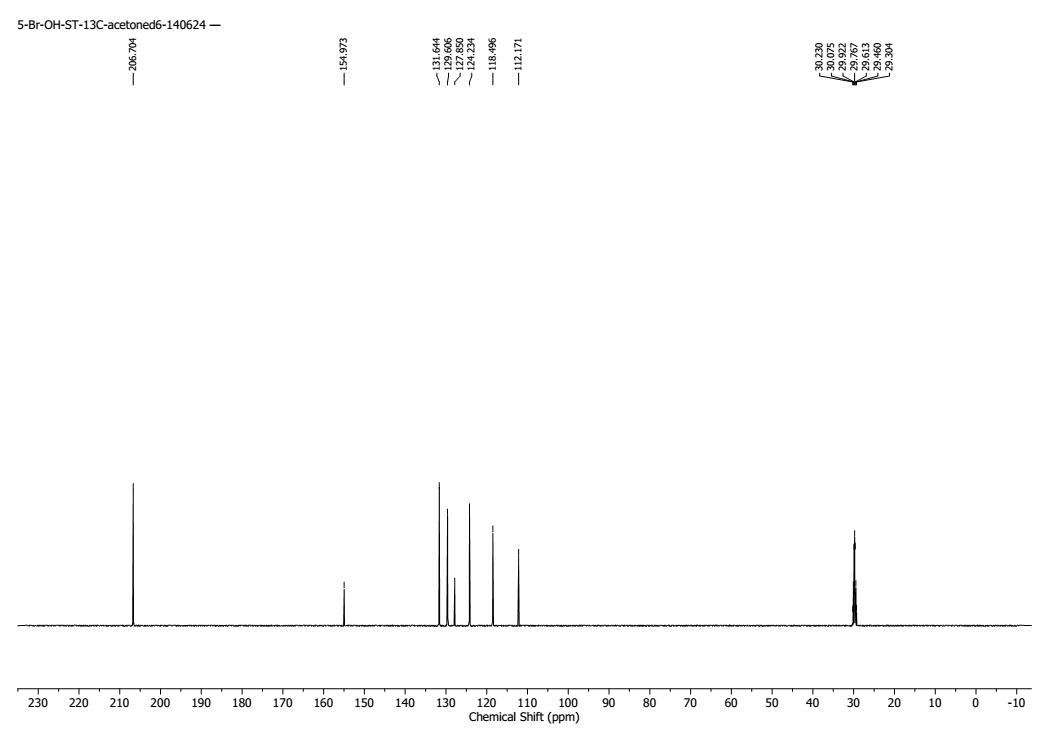


**Figure S24: ^13^C NMR spectrum of ST19**


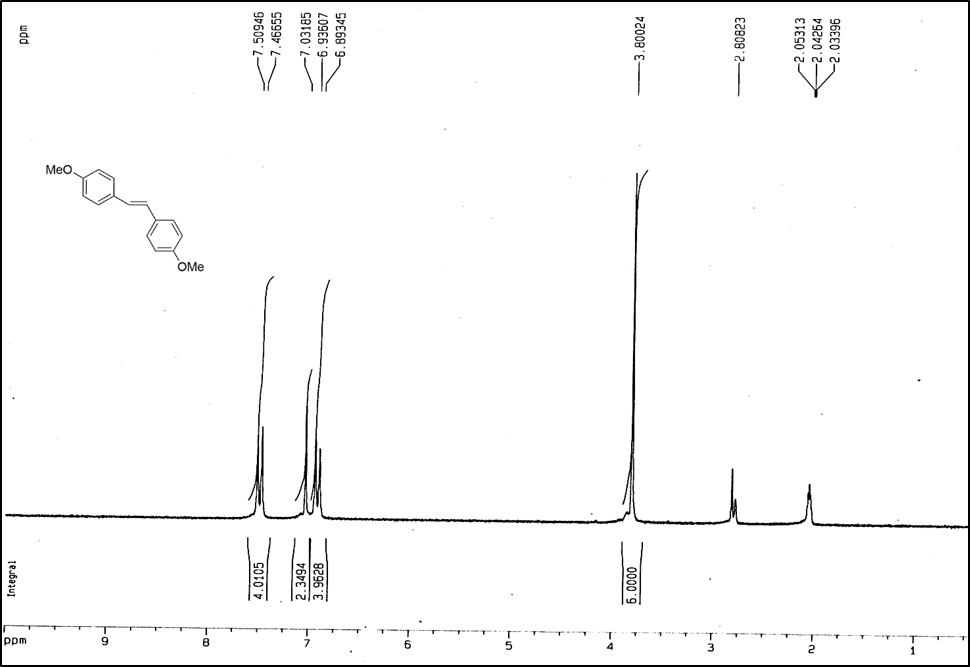


**Figure S25: ^1^H NMR spectrum of ST20**


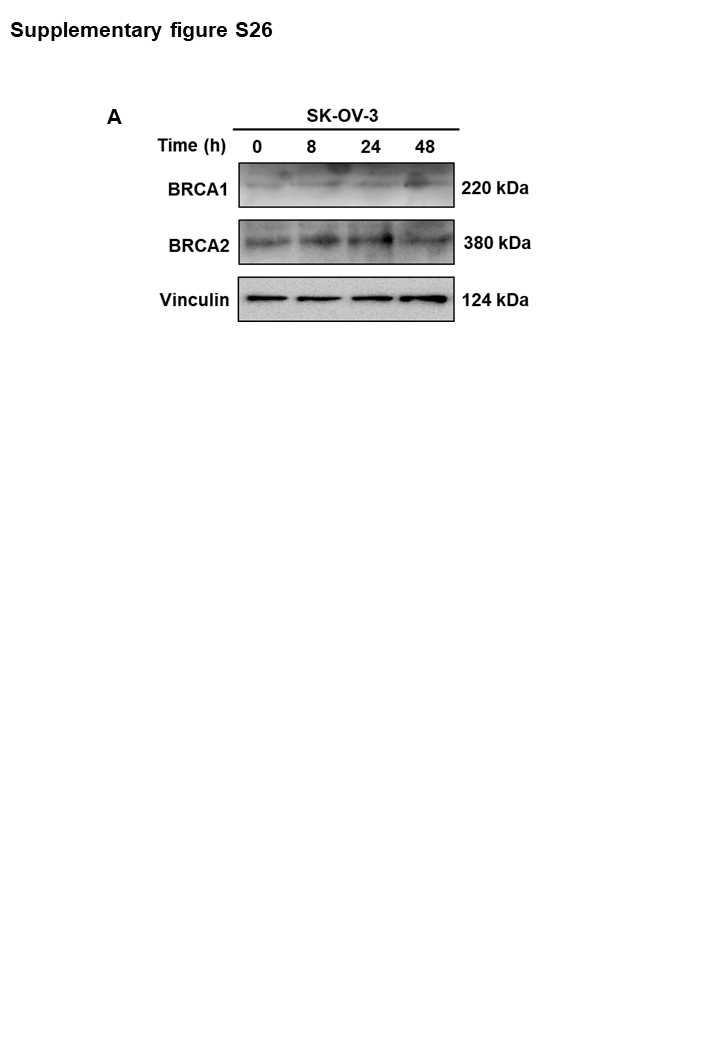


**Figure S26: Assessment of modulation of BRCA1 and BRCA2 proteins in the presence of DHS by western blotting.** SK-OV-3 cells were treated with DHS (10 µM) for 0-48 h and cell extract was prepared to analyse BRCA1 and BRCA2 protein level by western blotting. Representative blots are shown.


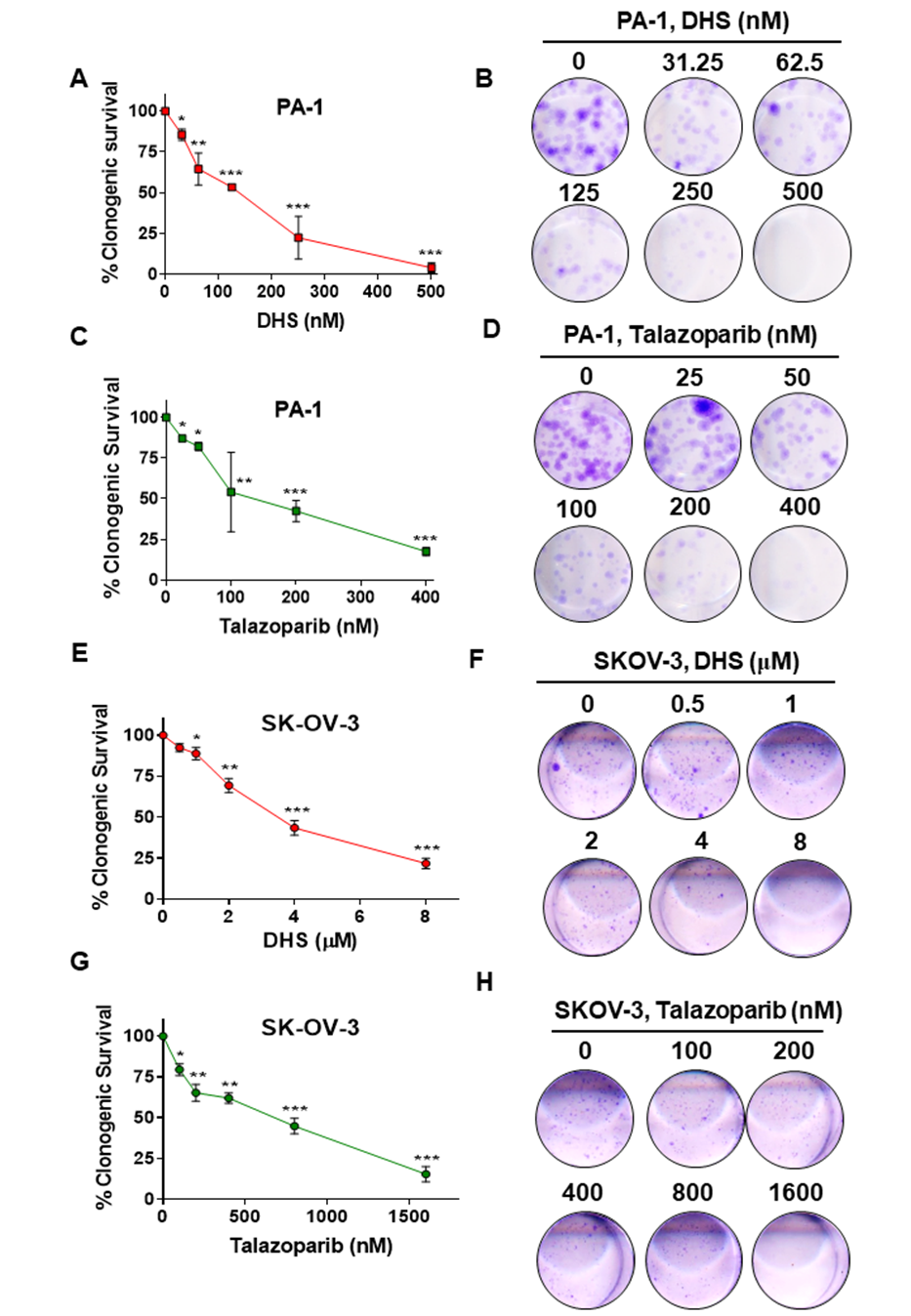


**Figure S27: Effect of DHS and talazoparib on colony formation in ovarian cancer cells** (A-D) PA-1 ovarian cancer cells were treated with vehicle control or increasing concentrations of DHS or talazoparib alone for 6-8 days and colony formation was assessed by clonogenic assay. Representative images of colonies in control, DHS, talazoparib treatments are shown in B and D. Respective quantification is shown in A and C. N=3 biological replicates. (E-H) SK-OV-3 ovarian cancer cells were treated with vehicle control or increasing concentrations of DHS or talazoparib alone for 7-9 days and colony formation was assessed by clonogenic assay. Representative images of colonies in control, DHS, talazoparib treatments are shown in F and H. Respective quantification is shown in E and G. N=3 biological replicates. Values indicated are Mean ± S.D. **p < 0.05, **p < 0.01 and ***p < 0.001* compared to the respective untreated group (ANOVA).


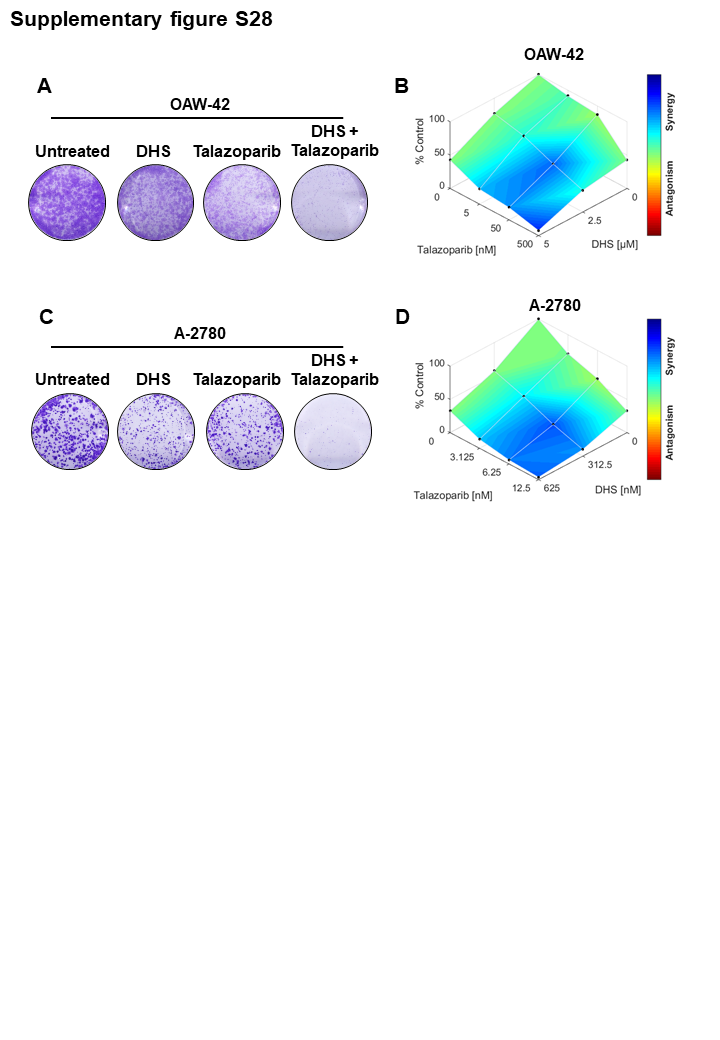


**Figure S28:** **Effect of the combination of DHS and talazoparib on the clonogenic survival of OAW42 and A2780 ovarian cancer cells** (A, B) OAW42 ovarian cancer cells were treated with vehicle control or DHS and talazoparib alone or their combination for 6 days and colony formation was assessed by clonogenic assay. Representative images of colonies in control, DHS (nM), talazoparib (nM) and their combination is shown. Quantification in the Combenefit-based plot for synergistic interaction between DHS and talazoparib is shown in B. N=2 biological replicates. (C, D) A2780 ovarian cancer cells were treated with vehicle control or DHS and talazoparib alone or their combination for 6 days and colony formation was assessed by clonogenic assay. Representative images of colonies in control, DHS (nM), talazoparib (nM) and their combination is shown. Quantification in the Combenefit-based plot for synergistic interaction between DHS and talazoparib is shown in D. N=2 biological replicates.


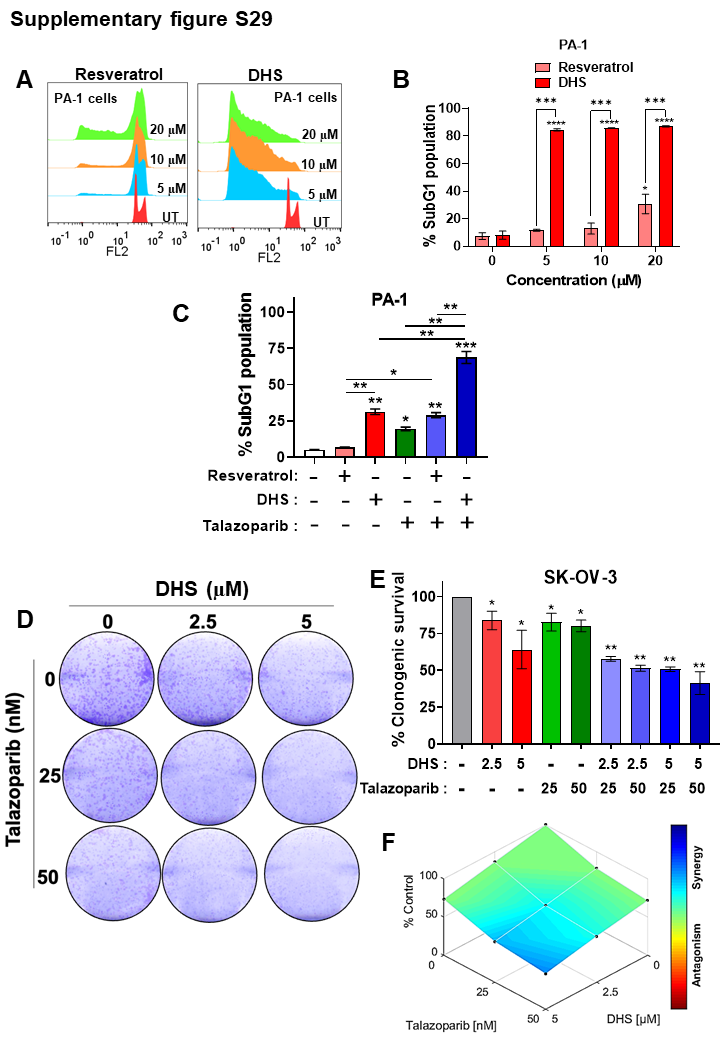


**Figure S29: Comparative effect of DHS and resveratrol and their synergy with talazoparib in inducing cell death.** (A-C) PA-1 ovarian cancer cells were treated with vehicle control or DHS, resveratrol alone or combination of talazoparib *plus* resveratrol or talazoparib *plus* DHS for 48 h and sub-G1 analysis were carried out by Flow cytometry. Quantifications of the sub-G1 analysis are shown in B and C. (D-F) Effect of DHS and talazoparib on clonogenic growth. SK-OV-3 cells were treated with vehicle control or DHS and talazoparib alone or their combination for 7-10 days, and colony formation was assessed by clonogenic assay. Quantifications of the clonogenic survival data are shown in E. Combenefit based plot for synergistic interaction between DHS and talazoparib is shown in F. N=3 biological replicates. Values indicated are Mean ± S.E.M. ns: non-significant, **p < 0.05, **p < 0.01 and ***p < 0.001* compared to the respective untreated group or between the groups being compared (ANOVA with Tukey post-hoc analysis). Inter-group comparisons are indicated.


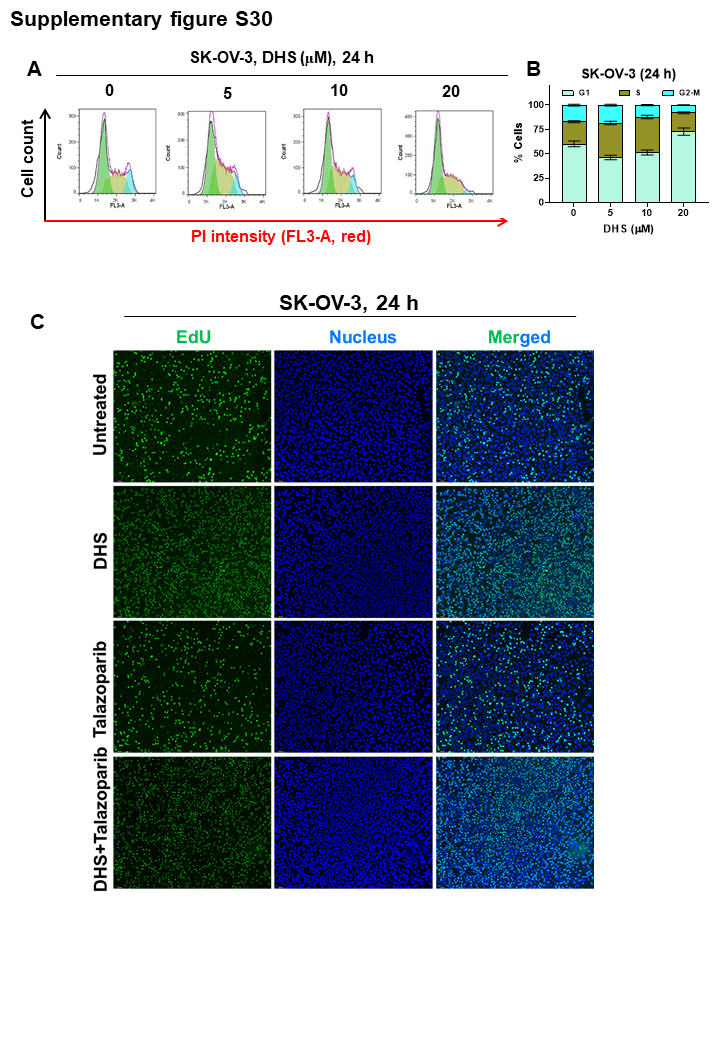


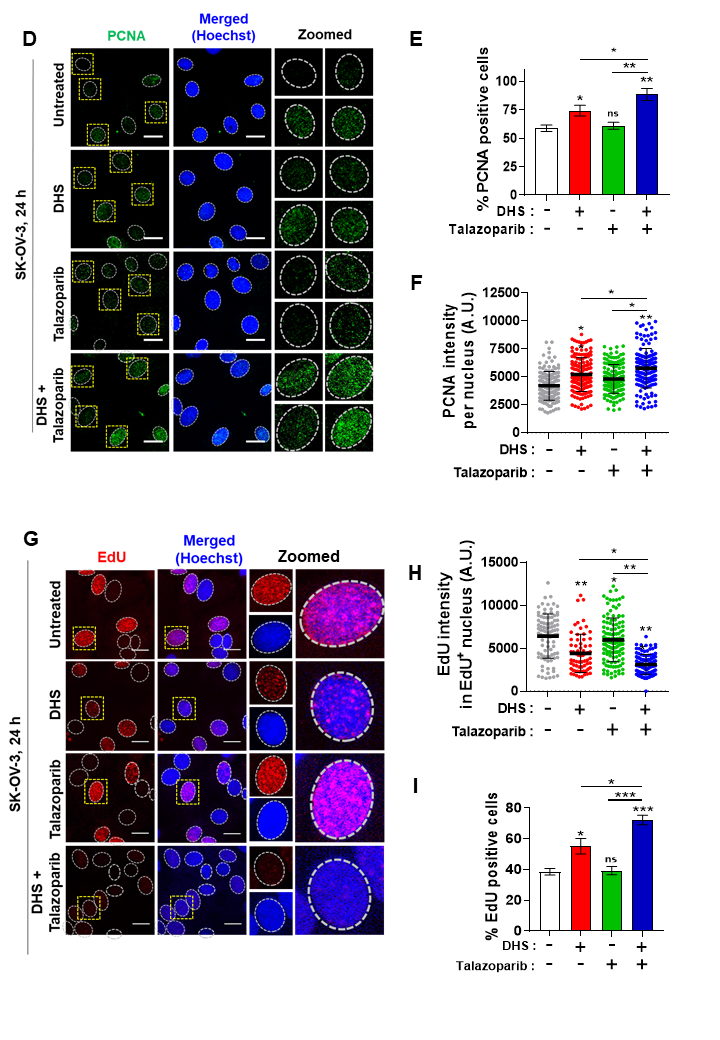
**Figure S30: The effects of DHS and talazoparib on cell cycle and replication foci.** (A, B) SK-OV-3 cells were treated with DHS (0-20 µM) for 24 h and cell cycle analysis was performed by flow cytometry. Distribution of cells in cell cycle was quantified by Flow-Jo software and shown in B. Only the profile of the gated population of cycling cells (G1, S or G2/M phase of the cell cycle) is shown in B. Sub-G1 cells are gated out from the analysis. Values indicated are Mean ± S.E.M. (C) SK-OV-3 cells were treated with vehicle or DHS (10 µM) and talazoparib (50 nM) alone or their combination for 24 h. EdU was added in the last 30 min, cells were fixed and stained for EdU using click-iT reaction kit as in Figure 4F, G. The images were captured at low magnification (1.6x objective) for acquiring a large number of cells and the images are shown. (D-F) SK-OV-3 cells were treated with DHS (5 µM), talazoparib (50 nM), or their combination for 24 h. PCNA foci formation was assessed by immunofluorescence microscopy. Four representative nuclei with PCNA nuclear foci in each condition were zoomed and shown in D. PCNA positive cells are indicated and the intensity of PCNA per nucleus is shown in E and F, respectively. Scale bar: 20 µm. (N=2 biological replicates) (G-I) SK-OV-3 cells were treated with vehicle or DHS (5 µM) and talazoparib (50 nM) alone or their combination for 24 h. EdU was added in the last 30 min, cells were fixed and stained for EdU (red) using click-iT reaction kit. Scale bar: 20 µm. Values indicated are Mean ± S.D. **p < 0.05, **p < 0.01 and ***p < 0.001* compared to the respective untreated group or between the groups being compared (ANOVA with Tukey post-hoc analysis). Inter-group comparisons are indicated.


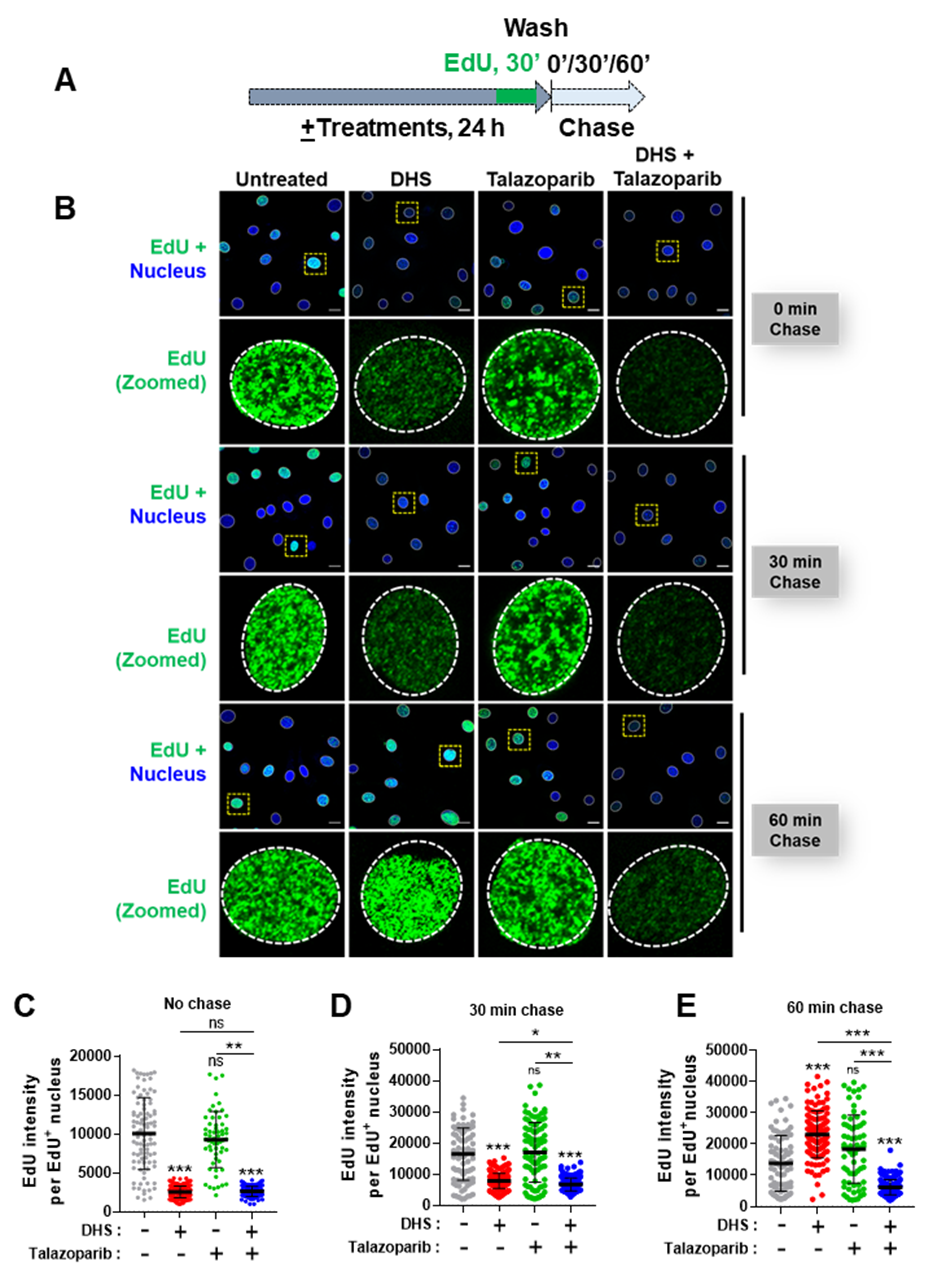


**Figure S31: The effect of DHS and talazoparib on replication foci during recovery.** (A) Scheme of the treatment and EdU incorporation. SK-OV-3 cells were treated with vehicle, DHS (10 µM) and talazoparib (50 nM) alone or their combination for 24 h. EdU was added in the last 30 min of the incubation duration and following wash off of treatments, a recovery/chase in the plain medium was given for 0-, 30- and 60-min. Cells were fixed and stained for EdU using click reaction kit. Quantifications of EdU intensity per replicating nucleus (EdU^+^) were shown in C, D and E, for no chase (0 min chase), 30 min chase and 60 min chase, respectively. Scale bar: 20 µm. N=3 biological replicates. Values indicated are Mean ± S.D. ns: non-significant, **p < 0.05, **p < 0.01, ***p < 0.001* compared to the respective untreated group or between the groups being compared (ANOVA with Tukey post-hoc analysis). Inter-group comparisons are indicated.


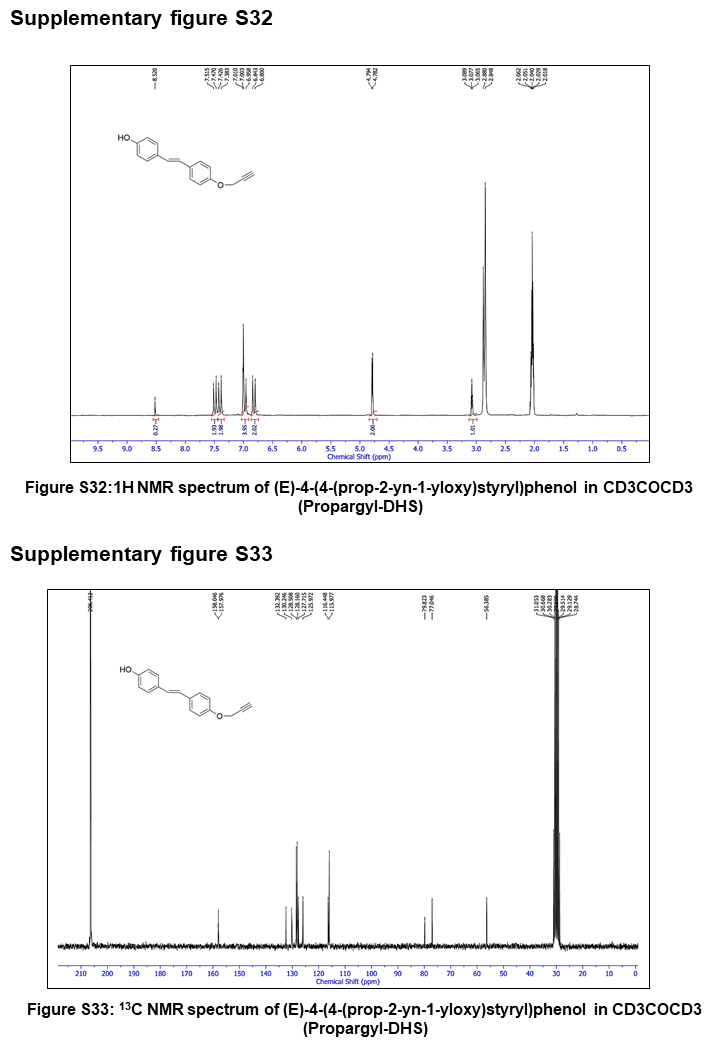


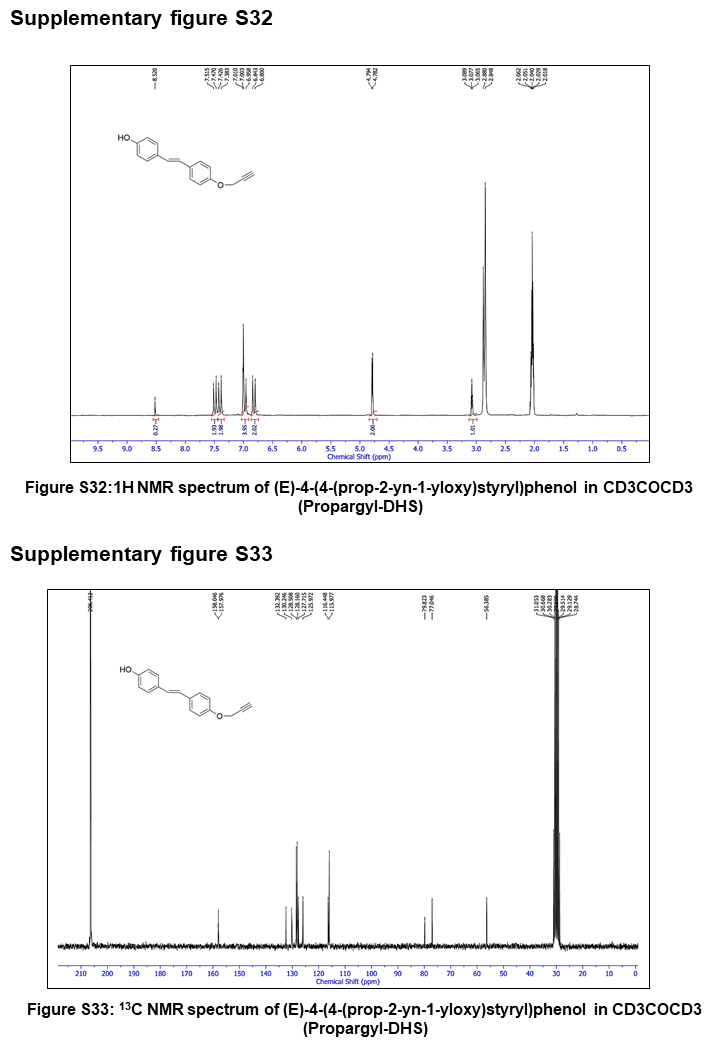
**Figure S32: 1H NMR spectrum of (E)-4-(4-(prop-2-yn-1-yloxy)styryl)phenol in CD3COCD3 (Propargyl-DHS)**

**Figure S33: ^13^C NMR spectrum of (E)-4-(4-(prop-2-yn-1-yloxy)styryl)phenol in CD3COCD3 (Propargyl-DHS)**


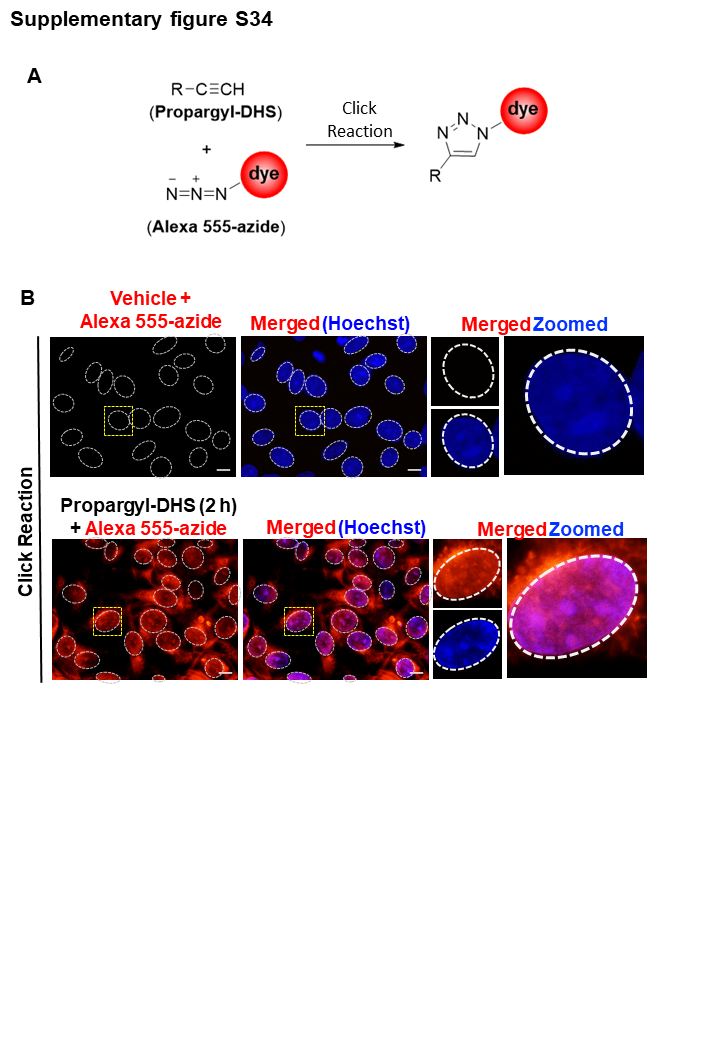


**Figure S34: Assessment of localization of propargyl-DHS in PA-1 cancer cells using click reaction.** (A) Schematic for click reaction of Propargyl-DHS and Alexa 555-azide**.** (B) SK-OV-3 cells were treated with vehicle propargyl-DHS (10 µM) for 2 h and subsequently fixed with methanol. Cells were permeabilized and detection of propargyl-DHS was assessed by click reaction, followed by microscopy.


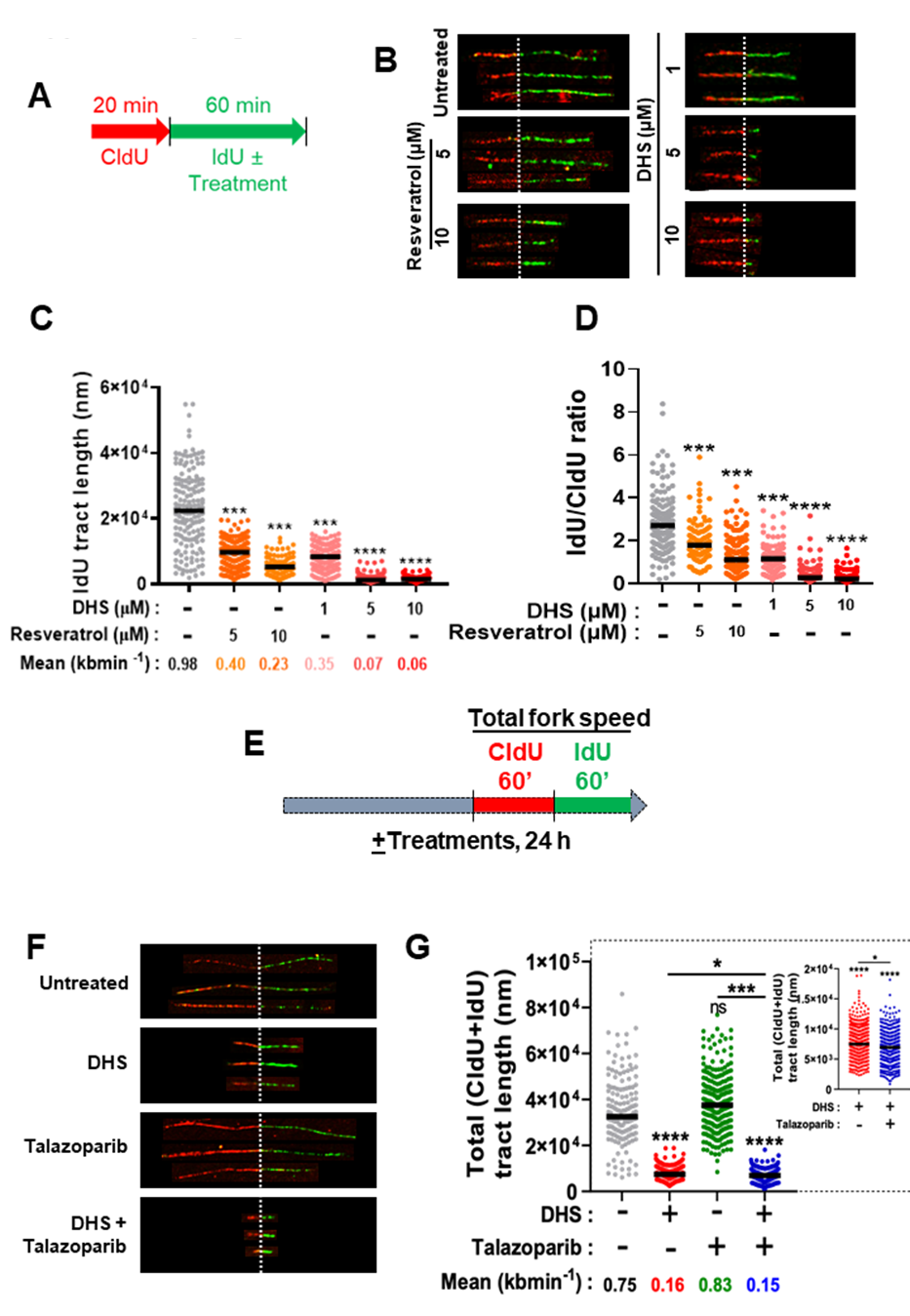


**Figure S35: The effect of DHS, resveratrol and talazoparib on replication fork progress at single molecule DNA fiber.** (A) Scheme of DNA Fiber assay: CldU and IdU incorporations in the absence/presence of DHS and resveratrol. SK-OV-3 cells were treated with CldU for 20 min, washed, treated with IdU for 60 min in the absence or presence of DHS and resveratrol. (B-E) Cells after treatments, as mentioned in A, were subjected to DNA fiber analysis. Representative images of DNA fibers of different treatments are shown in B. Quantification of IdU tract length, fork speed and ratio of IdU/CldU in fibers with both the labels (CldU+IdU) are shown in C and D. Bar represents median. N=2 biological replicates. Fibers scored: UT (n=153), Resveratrol (5 µM, n=224), Resveratrol (10 µM, n=99), DHS 1 µM (n=217), DHS 5 µM (n=487), DHS 10 µM (n=413). (F) Scheme of CldU and IdU incorporations in the absence/presence of DHS and talazoparib. SK-OV-3 cells were treated with DHS (10 µM), talazoparib (50 nM), or their combination for 24 h. For last 2 h of treatments, CldU was added for 60 min, cells were washed and treated with IdU for 60 min in the absence or presence of respective treatment. (G-H) Cells after treatments, as mentioned in F, were subjected to DNA fiber analysis. Representative images of DNA fibers of different treatments are shown in G. For inter comparison between DHS vs combination treatment, an expanded Y axis plot has been shown in the inset in G. Quantification of total tract length and fork speed in fibers with both the labels (CldU+IdU) are shown in H. N=2 biological replicates. Fibers scored: UT (n=147), DHS (n=910), Talazoparib (n=249), Combination (n=540). Bar represents median. ns: non-significant, **p < 0.05, ***p < 0.001 and ****p < 0.0001* compared to the respective untreated group or between the groups being compared (Kruskal-Wallis test with Dunn’s post-hoc analysis). Inter-group comparisons are indicated.


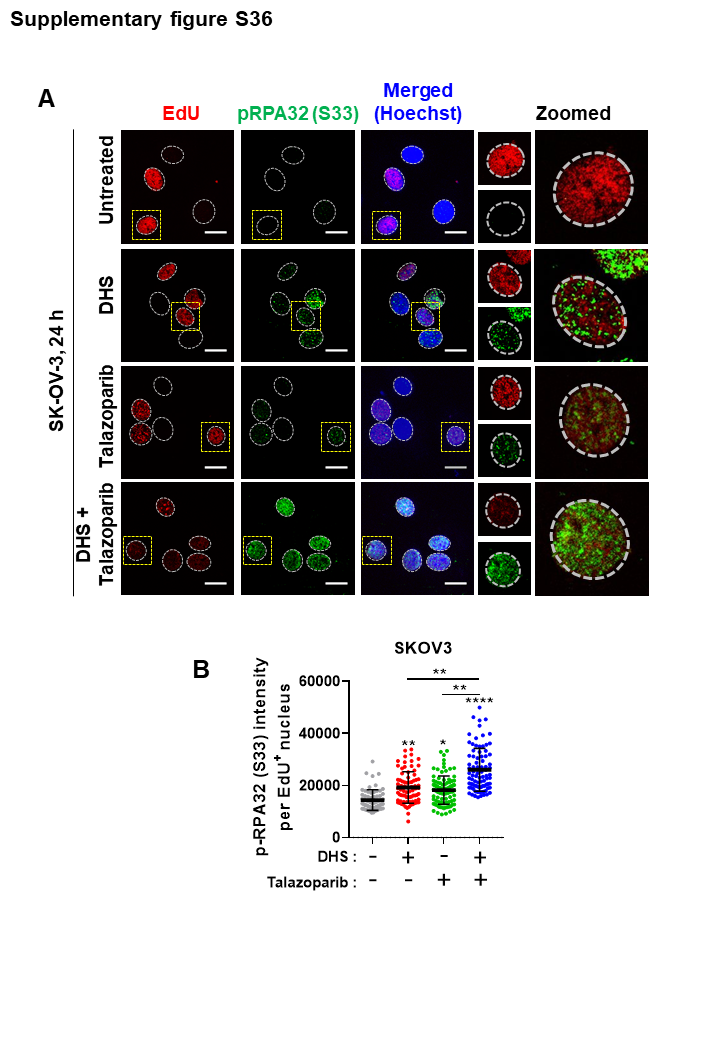


**Figure S36: The effect of lower concentration of DHS (5 µM) and talazoparib on ssDNA gaps in replicating cells as assessed by pRPA32 level in SK-OV-3 cells**

(A, B) Analysis for RPA32 phosphorylation. SK-OV-3 cells were treated with vehicle, DHS (5 µM) and talazoparib (50 nM) alone or their combination for 24 h. EdU was added for last 30 min of the treatment duration. RPA2 phosphorylation (S33) (immunofluorescence) and EdU (click reaction) were analysed and shown in A. Quantifications for intensity of RPA2 phosphorylation in replicating cells (EdU^+^ cells) is shown in B. Values indicated are Mean ± S.D. **p < 0.05, **p < 0.01, and ****p < 0.0001* compared to the respective untreated group or between the groups being compared (ANOVA with Tukey post-hoc analysis). Inter-group comparisons are indicated.


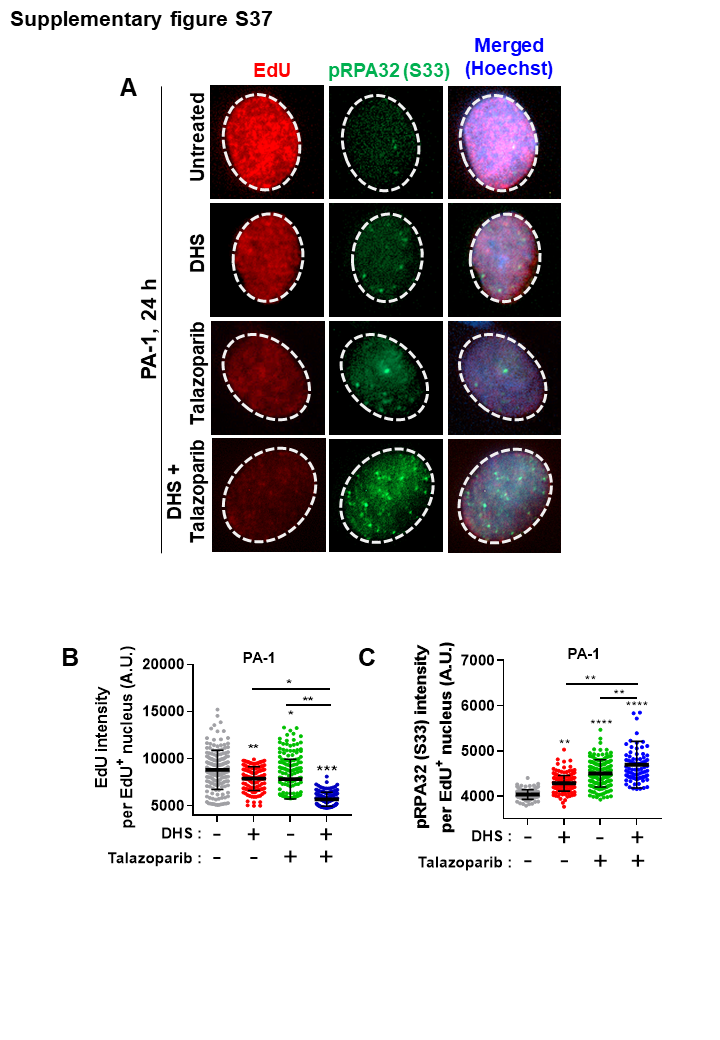


**Figure S37: The effect of DHS and talazoparib on ssDNA gaps in replicating cells as assessed by pRPA32 level in PA-1 cells.** (A, B) Analysis for RPA32 phosphorylation. PA-1 cells were treated with vehicle, DHS (100 nM) and talazoparib (50 nM) alone or their combination for 24 h. EdU was added for last 30 min of the treatment duration. RPA2 phosphorylation (S33) (immunofluorescence) and EdU (click reaction) were analysed and shown in A. Quantifications for EdU intensity and intensity of RPA2 phosphorylation in replicating cells (EdU^+^ cells) is shown in B and C respectively. Values indicated are Mean ± S.D. **p < 0.05, **p < 0.01, ***p < 0.001 and ****p < 0.0001* compared to the respective untreated group or between the groups being compared (ANOVA with Tukey post-hoc analysis). Inter-group comparisons are indicated.


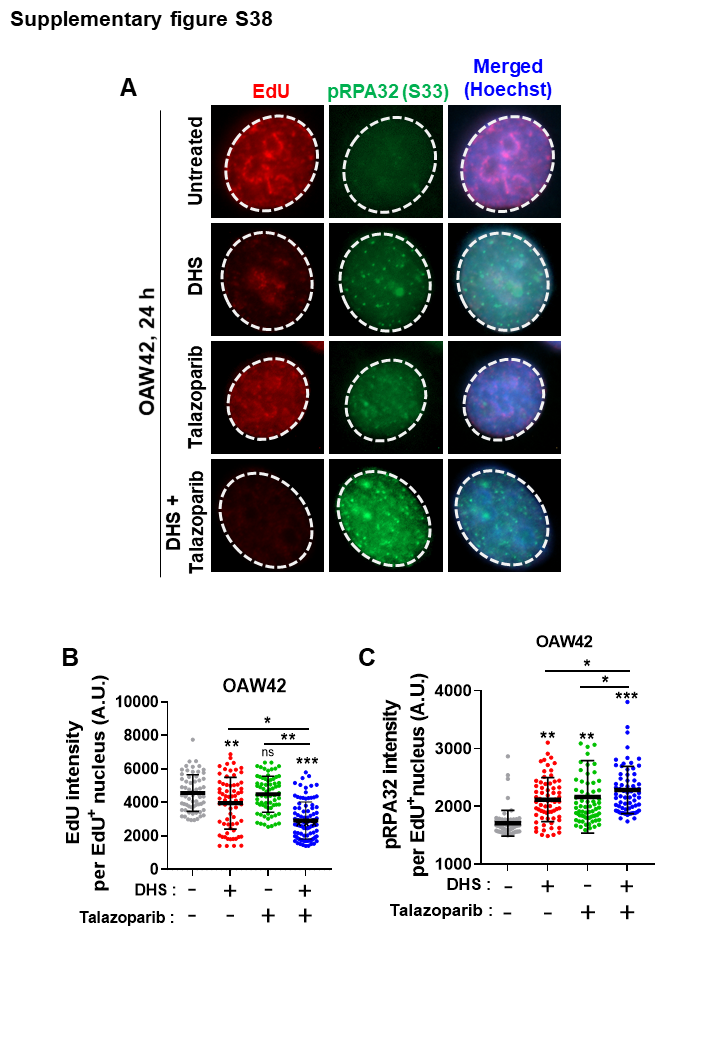


**Figure S38: The effect of DHS and talazoparib on ssDNA gaps in replicating cells as assessed by pRPA32 level in OAW42 cells**

(A, B) Analysis for RPA32 phosphorylation. OAW42 cells were treated with vehicle, DHS (5 µM) and talazoparib (50 nM) alone or their combination for 24 h. EdU was added for last 30 min of the treatment duration. RPA2 phosphorylation (S33) (immunofluorescence) and EdU (click reaction) were analysed and shown in A. Quantifications for EdU intensity and intensity of RPA2 phosphorylation in replicating cells (EdU^+^ cells) is shown in B and C respectively. Values indicated are Mean ± S.D. ns: non-significant, **p < 0.05, **p < 0.01, ***p < 0.001 and ****p < 0.0001* compared to the respective untreated group or between the groups being compared (ANOVA with Tukey post-hoc analysis). Inter-group comparisons are indicated.


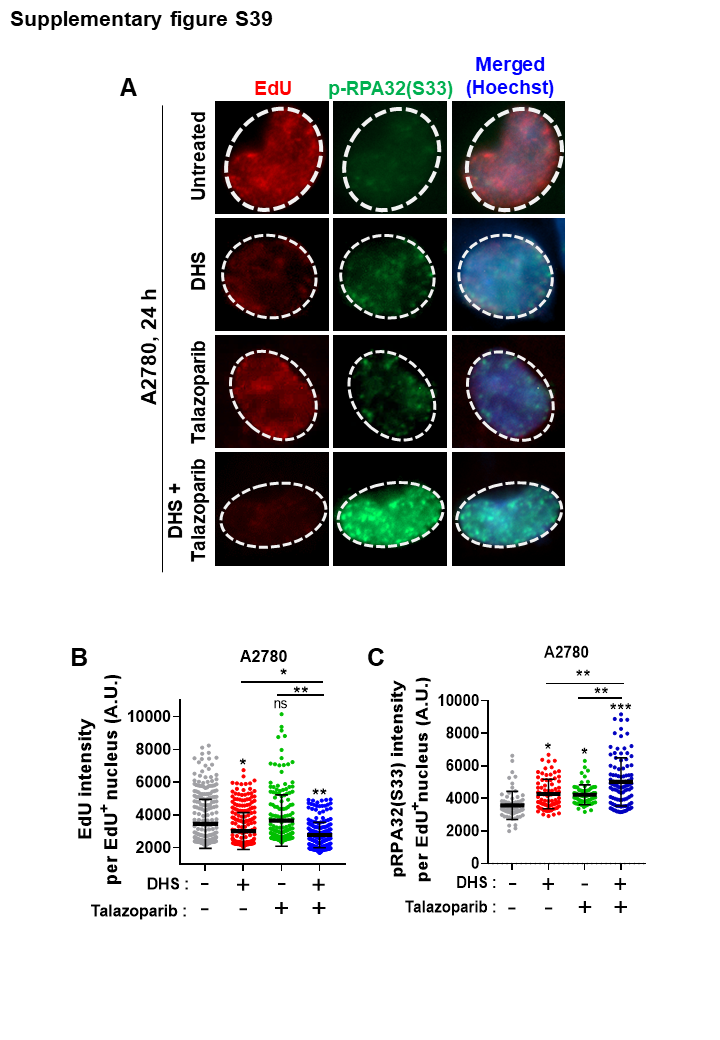


**Figure S39: The effect of DHS and talazoparib on ssDNA gaps in replicating cells as assessed by pRPA32 level in A2780 cells**

(A, B) Analysis for RPA32 phosphorylation. A2780 cells were treated with vehicle, DHS (500 nM) and talazoparib (25 nM) alone or their combination for 24 h. EdU was added for last 30 min of the treatment duration. RPA2 phosphorylation (S33) (immunofluorescence) and EdU (click reaction) were analysed and shown in A. Quantifications for EdU intensity and intensity of RPA2 phosphorylation in replicating cells (EdU^+^ cells) is shown in B and C respectively. Values indicated are Mean ± S.D. ns: non-significant, **p < 0.05, **p < 0.01, ***p < 0.001 and ****p < 0.0001* compared to the respective untreated group or between the groups being compared (ANOVA with Tukey post-hoc analysis). Inter-group comparisons are indicated.


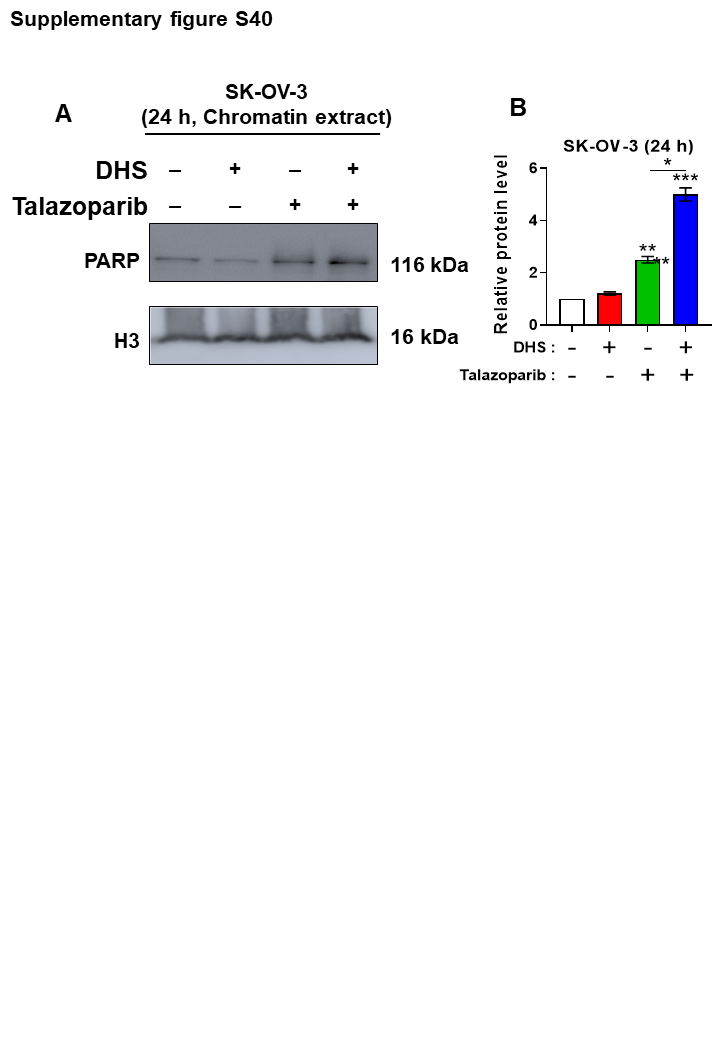


**Figure S40: Combination of DHS and talazoparib enhances PARP trapping on chromatin.** SK-OV-3 cells were treated with talazoparib (50 nM), DHS (10 µM) or their combination for the 24 h, chromatin lysate preparation was carried out after cell harvesting and the level of PARP protein was analysed by immunoblotting. H3 was used as loading control. Values indicated are mean ± S.D. **p<0.05, **p<0.01 and ***p<0.001* compared to the respective treatment in the control group or for intergroup comparisons (ANOVA with Tukey post-hoc analysis).

**
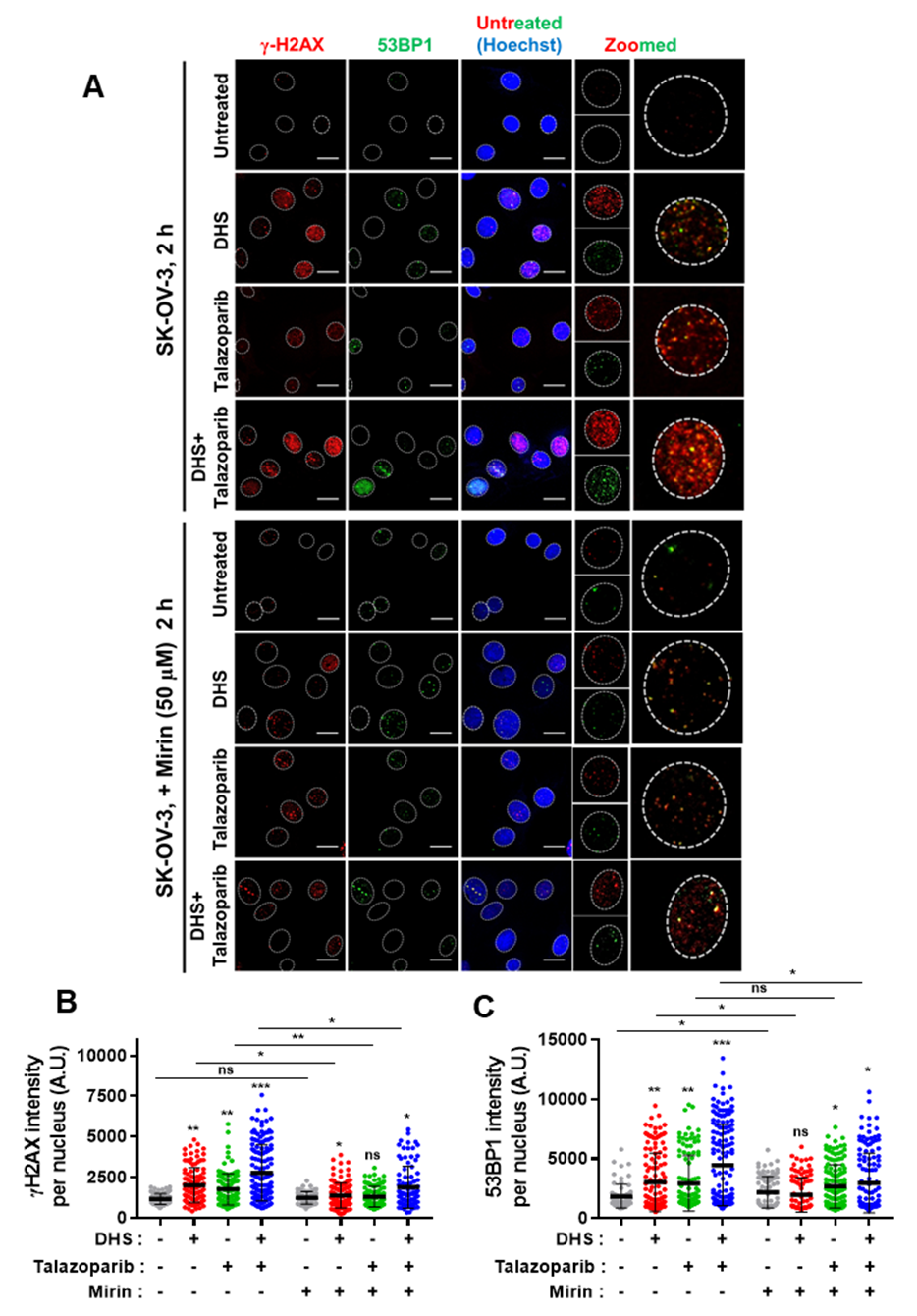
**

**Figure S41: Role of MRE11 in replication fork processing and generation of DSBs in response to DHS and talazoparib. (**A, B) Effect of MRE11 on γH2AX and 53BP1 formation. SK-OV-3 cells were treated with DHS (5 µM), talazoparib (50 nM), or their combination for 2 h in the absence or presence of mirin (50 μM) and with its pre-treatment as per the scheme in Figure 7H. Cells were fixed and analysed for γH2AX and 53BP1 signals by immunofluorescence microscopy. Intensities of γH2AX and 53BP1 per nucleus were measured and shown in A and B respectively. (N = 2 biological replicates). Values indicated are mean ± S.D. ns: non-significant, **p<0.05, **p<0.01, ***p<0.001,* *****p<0.0001* compared to the respective treatment in the control group or for intergroup comparisons (ANOVA with Tukey post-hoc analysis).


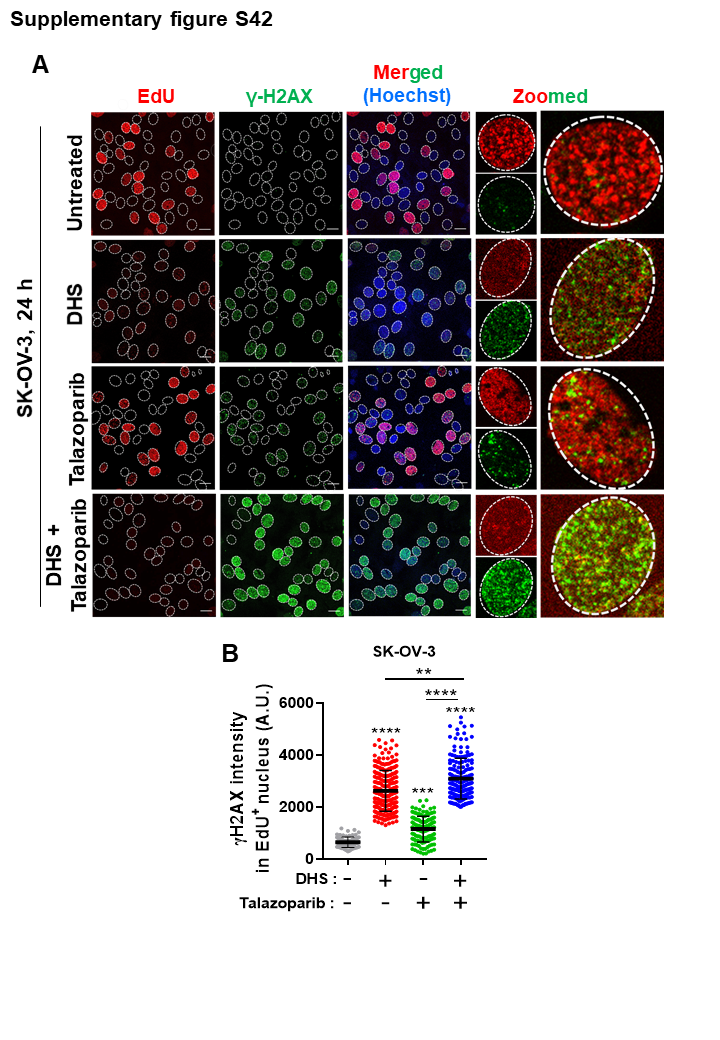


**Figure S42: The effects of DHS and talazoparib on nascent replication foci and** **γH2AX foci formation** (A-C) SK-OV-3 cells were treated with DHS (10 µM), talazoparib (50 nM), or their combination for 24 h. During last 30 min of above treatments, EdU was added to label the ongoing replication foci. Cells were washed, fixed and analysed for EdU (click reaction) and γH2AX (immunofluorescence) and representative images were shown in A. Intensities of γH2AX per replicating nucleus (EdU^+^) were measured and shown in B. Note that the Intensity of the zoomed image of EdU stained nucleus in E in DHS and combination treatment conditions have been enhanced to show colocalization more clearly. N=3 biological replicates. Values indicated are Mean ± S.D. ns: non-significant, **p < 0.05, ***p < 0.001 and ****p < 0.0001* compared to the respective untreated group or between the groups being compared (ANOVA with Tukey post-hoc analysis). Inter-group comparisons are indicated.


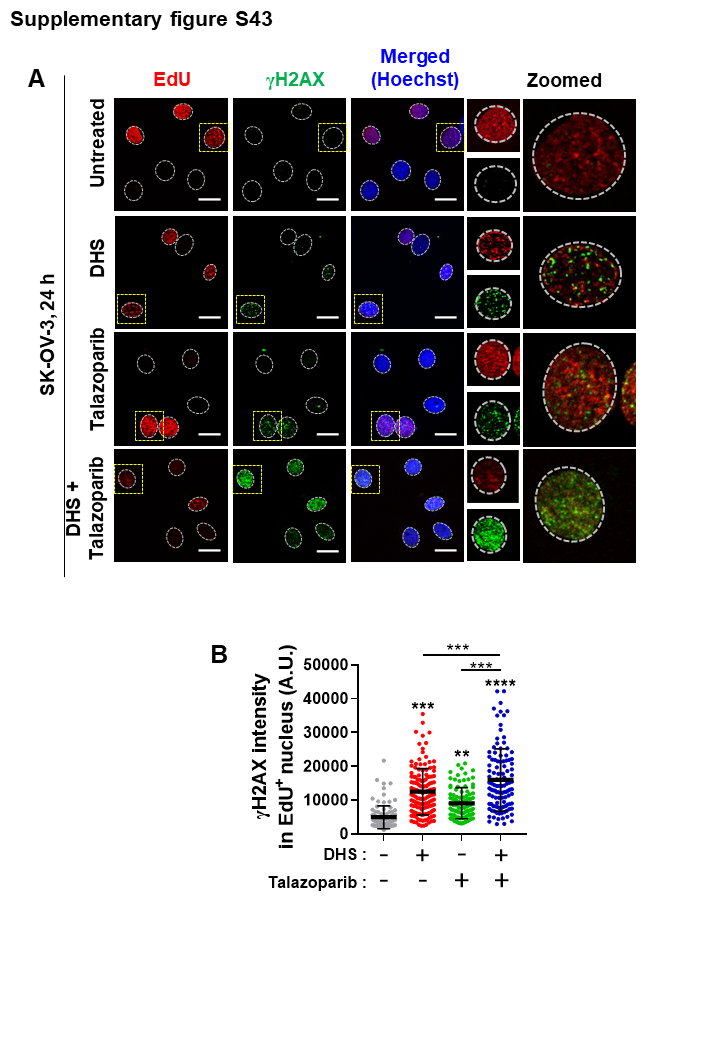
**Figure S43: The effects of DHS and talazoparib on nascent replication foci and γH2AX foci formation in SK-OV-3 at lower DHS concentration (**A-B) SK-OV-3 cells were treated with DHS (5 µM), talazoparib (50 nM), or their combination for 24 h. During last 30 min of above treatments, EdU was added to label the ongoing replication foci. Cells were washed, fixed and analysed for EdU (click reaction) and γH2AX (immunofluorescence) and representative images were shown in A. Intensities of γH2AX per replicating nucleus (EdU^+^) were measured and shown in B. N=2 biological replicates. Values indicated are Mean ± S.D. ***p < 0.01, ***p < 0.001 and ****p < 0.0001* compared to the respective untreated group or between the groups being compared (ANOVA with Tukey post-hoc analysis). Inter-group comparisons are indicated.


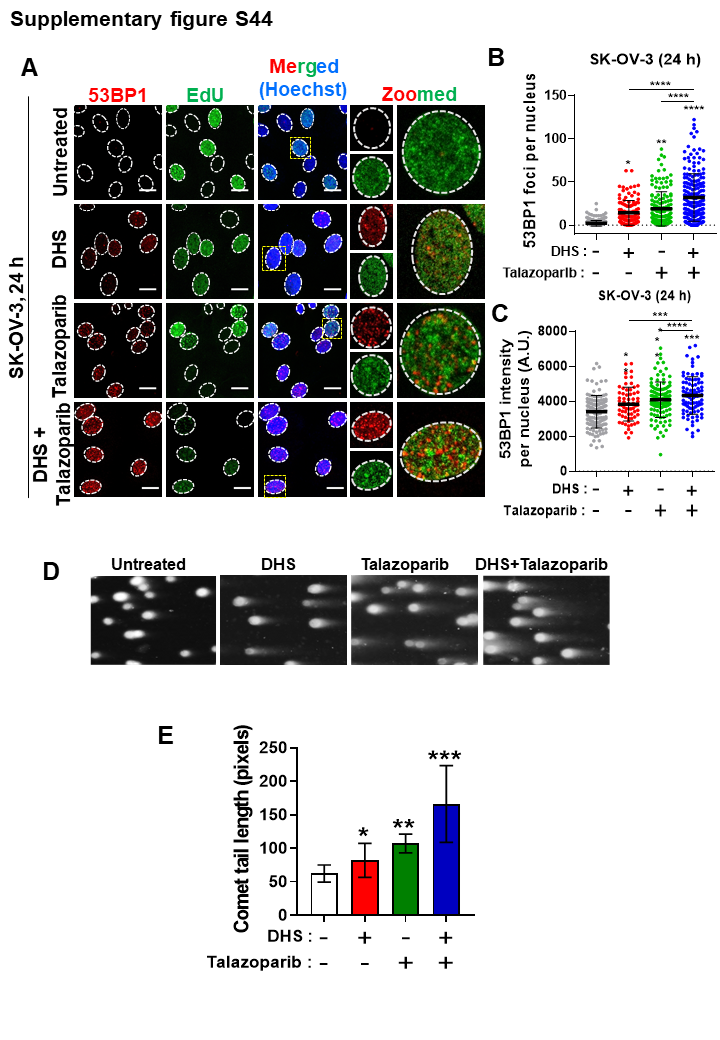
**Figure S44: The effects of DHS and talazoparib on forks collapse and generation of DSBs** (A-C) SK-OV-3 cells were treated with DHS (10 µM), talazoparib (50 nM), or their combination for 24 h. During last 30 min of above treatments, EdU was added to label the ongoing replication foci. Cells were washed, fixed and analysed for EdU (click reaction) and 53BP1 (immunofluorescence) and representative images were shown. Number of 53BP1 foci and intensities of 53BP1 foci per replicating nucleus were measured and shown in B and C, respectively. (D. E) Neutral comet assay: SK-OV-3 cells were treated with DHS (10 µM), talazoparib (50 nM), or their combination for 24 h. Neutral comet assay was carried out to assess DSBs. Representative comet images were shown in D and quantification of comet tail length were shown in E. N=3 biological replicates. Values indicated are Mean ± S.D. **p < 0.05, **p < 0.01, ***p < 0.001 and ****p < 0.0001* compared to the respective untreated group or between the groups being compared (ANOVA with Tukey post-hoc analysis). Inter-group comparisons are indicated.


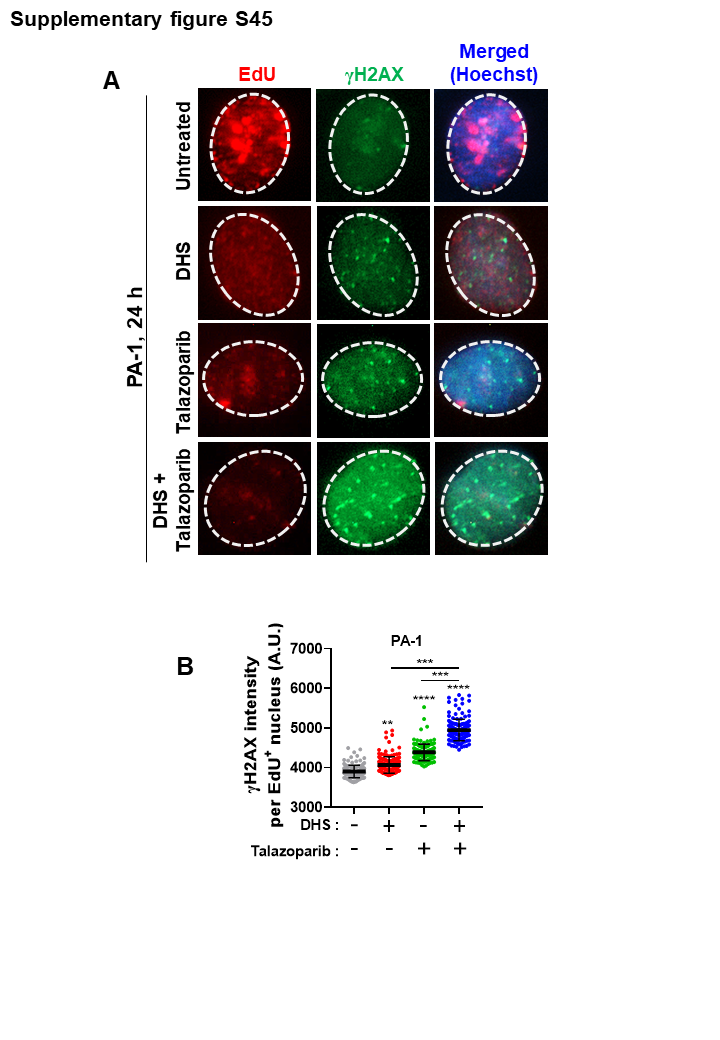
**Figure S45: The effects of DHS and talazoparib on nascent replication foci and generation of γH2AX foci in PA-1 cells.** (A-B) PA-1 cells were treated with DHS (100 nM), talazoparib (50 nM), or their combination for 24 h. During last 30 min of above treatments, EdU was added to label the ongoing replication foci. Cells were washed, fixed and analysed for EdU (click reaction) and γH2AX (immunofluorescence) and representative images were shown in A. Intensities of γH2AX per EdU^+^ nucleus was measured and shown in B. N=2 biological replicates. Values indicated are Mean ± S.D., ***p < 0.01, ***p < 0.001 and ****p < 0.0001* compared to the respective untreated group or between the groups being compared (ANOVA with Tukey post-hoc analysis). Inter-group comparisons are indicated.


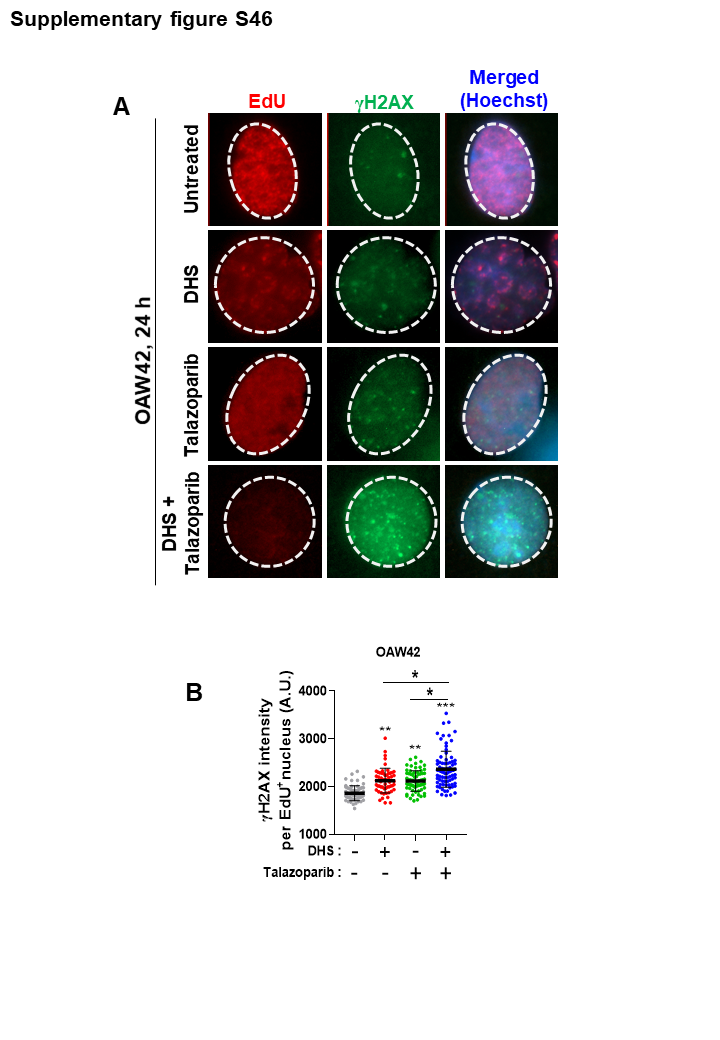


**Figure S46: The effects of DHS and talazoparib on nascent replication foci and generation of γH2AX foci in OAW42 cells.** (A-B) OAW-42 cells were treated with DHS (5 µM), talazoparib (50 nM), or their combination for 24 h. During last 30 min of above treatments, EdU was added to label the ongoing replication foci. Cells were washed, fixed and analysed for EdU (click reaction) and γH2AX (immunofluorescence) and representative images were shown in A. Intensities of γH2AX per EdU^+^ nucleus was measured and shown in B. N=2 biological replicates. Values indicated are Mean ± S.D., **p < 0.05,* ***p < 0.01 and, ***p < 0.001* compared to the respective untreated group or between the groups being compared (ANOVA with Tukey post-hoc analysis). Inter-group comparisons are indicated.


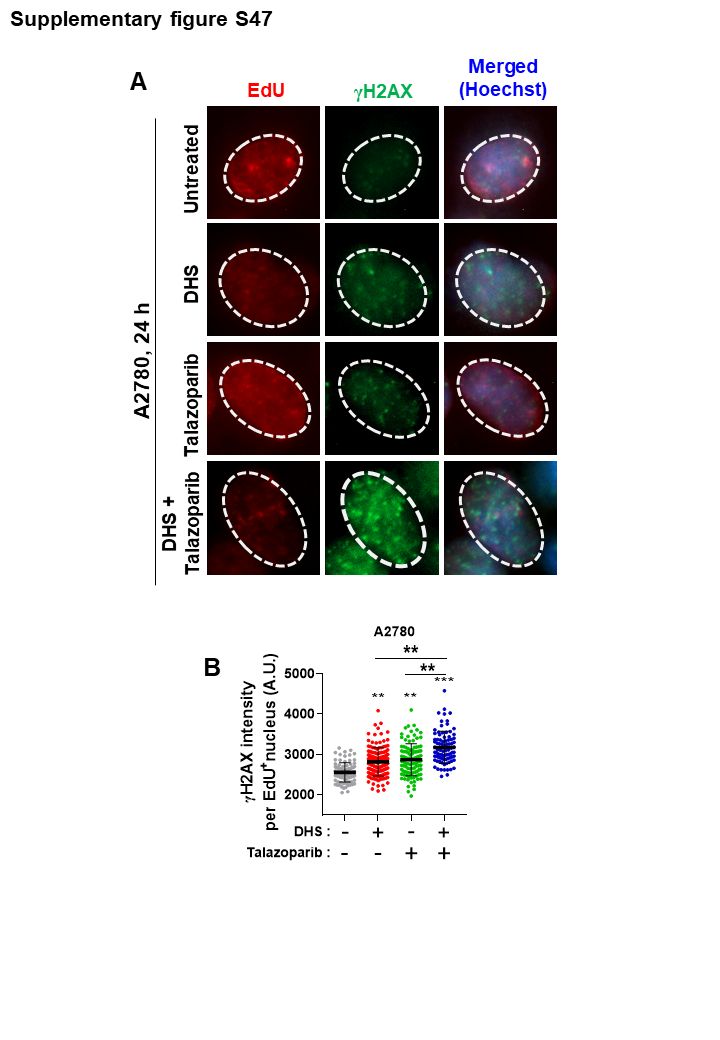
**Figure S47: The effects of DHS and talazoparib on nascent replication foci and generation of γH2AX foci in A2780 cells.** (A-B) A2780 cells were treated with DHS (500 nM), talazoparib (25 nM), or their combination for 24 h. During last 30 min of above treatments, EdU was added to label the ongoing replication foci. Cells were washed, fixed and analysed for EdU (click reaction) and γH2AX (immunofluorescence) and representative images were shown in A. Intensities of γH2AX per EdU^+^ nucleus was measured and shown in B. N=2 biological replicates. Values indicated are Mean ± S.D., ***p < 0.01 and ***p < 0.001* compared to the respective untreated group or between the groups being compared (ANOVA with Tukey post-hoc analysis). Inter-group comparisons are indicated.


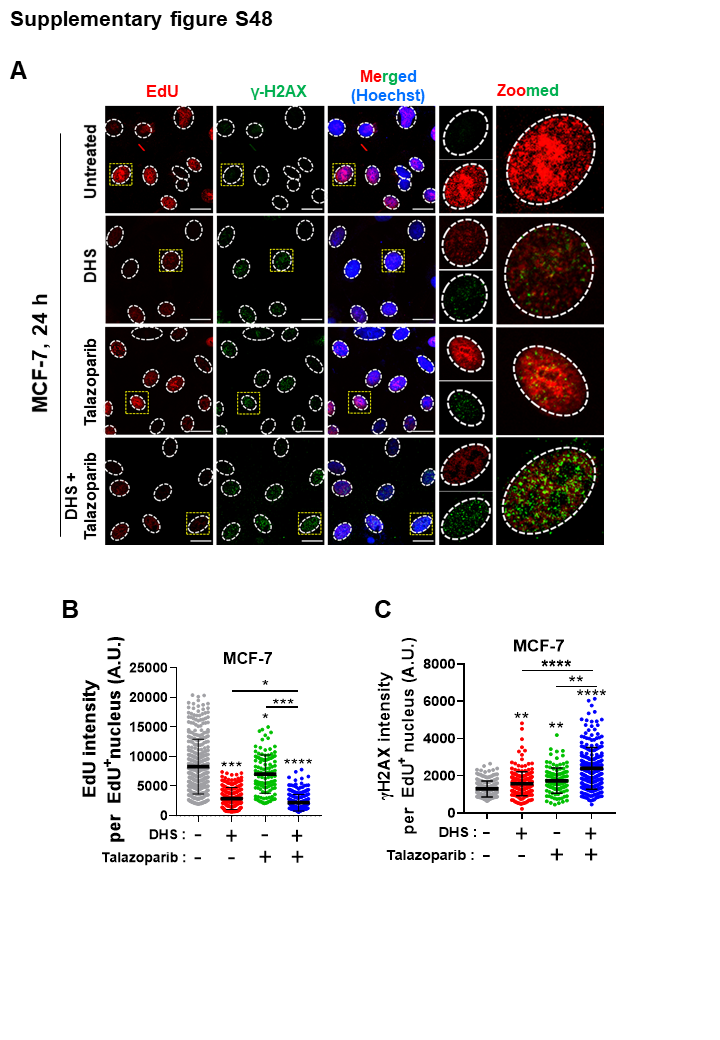


**Figure S48: The effects of DHS and talazoparib on nascent replications foci and generation of γH2AX foci in breast carcinoma cells** (A-C) MCF-7 cells were treated with DHS (10 µM), talazoparib (50 nM), or their combination for 24 h. During last 30 min of above treatments, EdU was added to label the ongoing replication foci. Cells were washed, fixed and analysed for EdU (click reaction) and γH2AX (immunofluorescence) and representative images were shown in A. Intensities of γH2AX and EdU per replicating nucleus were measured and shown in B and C, respectively. Values indicated are Mean ± S.D., **p < 0.05,* ***p < 0.01, ***p < 0.001 and ****p < 0.0001* compared to the respective untreated group or between the groups being compared (ANOVA with Tukey post-hoc analysis). Inter-group comparisons are indicated.


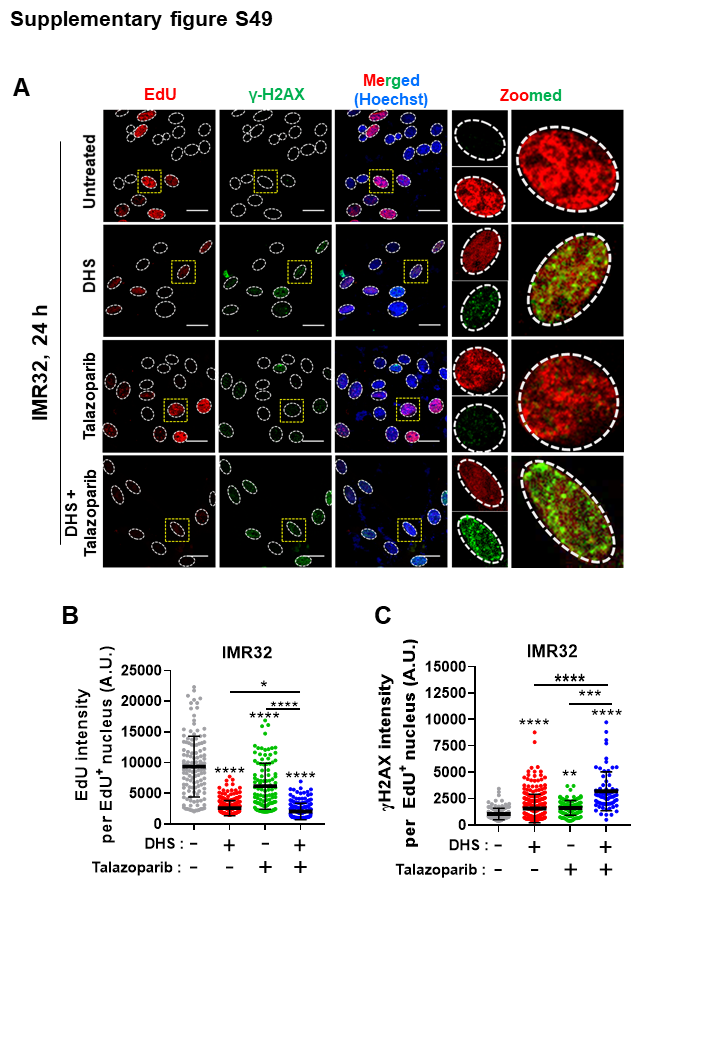


**Figure S49: The effects of DHS and talazoparib on nascent replication foci and generation of γH2AX foci in neuroblastoma cells** (A-C) IMR32 cells were treated with DHS (10 µM), talazoparib (50 nM), or their combination for 24 h. During last 30 min of above treatments, EdU was added to label the ongoing replication foci. Cells were washed, fixed and analysed for EdU (click reaction) and γH2AX (immunofluorescence) and representative images were shown in A. Intensities of γH2AX and EdU per replicating nucleus were measured and shown in B and C, respectively. Values indicated are Mean ± S.D., **p < 0.05,* ***p < 0.01, ***p < 0.001 and ****p < 0.0001* compared to the respective untreated group or between the groups being compared (ANOVA with Tukey post-hoc analysis). Inter-group comparisons are indicated.


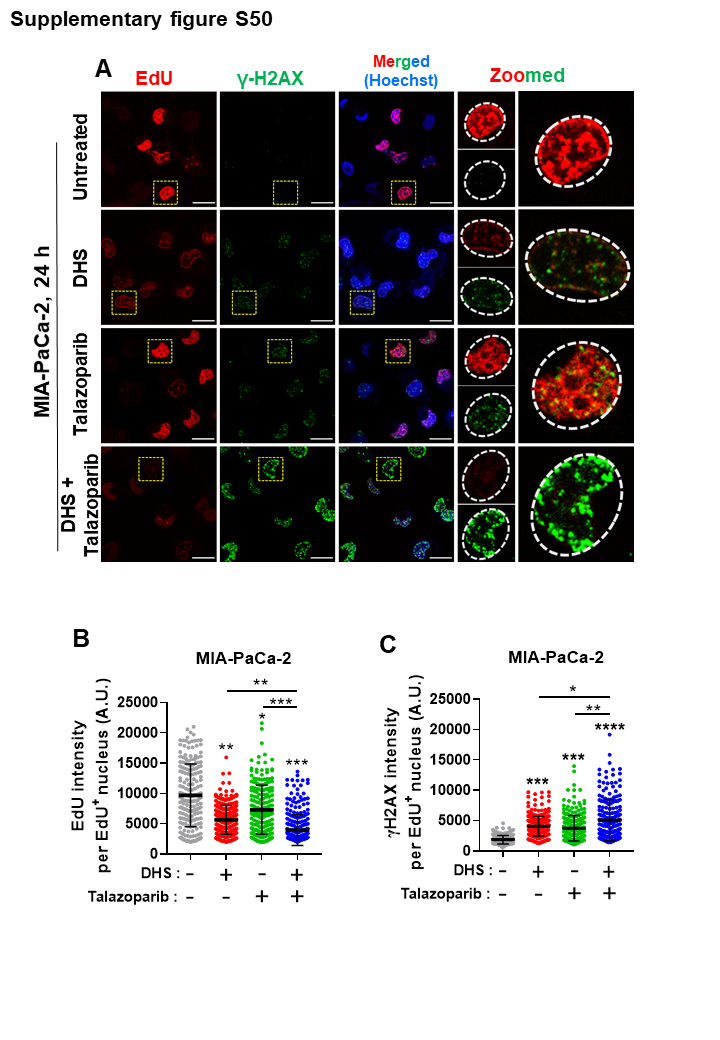
**Figure S50: The effects of DHS and talazoparib on nascent replication foci and generation of γH2AX foci in pancreatic ductal carcinoma cells** (A-C) MIA-PaCa2 cells were treated with DHS (10 µM), talazoparib (50 nM), or their combination for 24 h. During last 30 min of above treatments, EdU was added to label the ongoing replication foci. Cells were washed, fixed and analysed for EdU (click reaction) and γH2AX (immunofluorescence) and representative images were shown in A. Intensities of γH2AX and EdU per replicating nucleus were measured and shown in B and C, respectively. Values indicated are Mean ± S.D., **p < 0.05,* ***p < 0.01, ***p < 0.001 and ****p < 0.0001* compared to the respective untreated group or between the groups being compared (ANOVA with Tukey post-hoc analysis). Inter-group comparisons are indicated.
